# mMass as a Software Tool for the Annotation of Cyclic Peptide Tandem Mass Spectra

**DOI:** 10.1371/journal.pone.0044913

**Published:** 2012-09-13

**Authors:** Timo H. J. Niedermeyer, Martin Strohalm

**Affiliations:** 1 Institute of Pharmacy, Ernst-Moritz-Arndt University, Greifswald, Germany; 2 Cyano Biotech GmbH, Berlin, Germany; 3 Institute of Microbiology, v.v.i., Academy of Sciences of the Czech Republic, Czech Republic; Moffitt Cancer Center, United States of America

## Abstract

Natural or synthetic cyclic peptides often possess pronounced bioactivity. Their mass spectrometric characterization is difficult due to the predominant occurrence of non-proteinogenic monomers and the complex fragmentation patterns observed. Even though several software tools for cyclic peptide tandem mass spectra annotation have been published, these tools are still unable to annotate a majority of the signals observed in experimentally obtained mass spectra. They are thus not suitable for extensive mass spectrometric characterization of these compounds. This lack of advanced and user-friendly software tools has motivated us to extend the fragmentation module of a freely available open-source software, mMass (http://www.mmass.org), to allow for cyclic peptide tandem mass spectra annotation and interpretation. The resulting software has been tested on several cyanobacterial and other naturally occurring peptides. It has been found to be superior to other currently available tools concerning both usability and annotation extensiveness. Thus it is highly useful for accelerating the structure confirmation and elucidation of cyclic as well as linear peptides and depsipeptides.

## Introduction

Cyclic peptides are a group of natural products that attracts the interest of a large number of researchers due to their intriguing structures and powerful and diverse bioactivities. Cyclic peptides are found in many bacterial and fungal strains [1], [2]. Especially cyanobacteria have long been known for the wealth of cyclic peptides and cyclic depsipeptides they produce [3]–[6]. These compounds can be of ribosomal origin (e.g. the microviridins found in *Microcystis* strains) [7], [8], but predominantly they are synthesized by non-ribosomal peptide synthetases (NRPS) [9]–[12]. Non-ribosomal peptides are fascinating for the natural product chemist, as they are not only composed of proteinogenic amino acids but also contain unusual and highly modified amino acids or monomers constructed by polyketide synthases (PKS). These compounds often derive from a mixed PKS/NRPS biosynthetic pathway as described in great detail e.g. for the cyanobacterial microcystins [13]–[15]. Opposed to 20 proteinogenic amino acids, over 500 different monomers can be compiled from known non-ribosomal peptides and are publicly available in the NORINE database [2], [16]. Still, additional monomers are being described in the literature.

Among the most prominent examples of pharmaceutically exploited non-ribosomal cyclic peptides are cyclosporine, an immunosuppressant from *Tolypocladium inflatum* [17], [18], and vancomycin, an antibiotic from *Amycolatopsis orientalis* [19], [20]. Several other compounds or compound mixtures are either used directly (e.g. tyrotricin, daptomycin and actinomycin D) or serve as lead structures for current drug development (e.g. the cryptophycins) [21].

The structural characterization of newly isolated or synthesized bioactive cyclic peptides is the basis for their further exploitation. Mass spectrometry often serves as a valuable tool for the first steps in dereplication, structure confirmation and elucidation of cyclic peptides, when limited amount of material complicates direct characterization by NMR spectroscopy.

To validate proposed structures, theoretical *in-silico* fragmentation is commonly used for comparison with experimental data. Surveying freely available software that can be used for *in-silico* fragmentation of cyclic peptides and the annotation of their tandem mass spectra, we have found that despite the general interest in these compounds, currently available bioinformatic tools do not allow convenient and effective assignment of cyclic peptide tandem mass spectra. Driven by the needs of proteomics scientists, many software tools for *in-silico* fragmentation and annotation of linear peptides have been developed based on the observed gas phase fragmentation behavior of peptides [22]–[26] and theoretical aspects of fragmentation calculation [27]–[31]. These tools, however, are not necessarily suited for the natural product or synthetic chemist working with cyclic peptides.

Most of the available tools with a graphical user interface (GUI; e.g. ProteinProspector [32], InSilicoSpectro [33], [34], Proteomics Toolkit [35], PeptideART [36], mMass [37]) are suited for the *in-silico* fragmentation and annotation of linear peptides but do not offer the same functionality for cyclic peptides. In addition, mainly the proteinogenic amino acids can be used to assemble a peptide sequence, and support for unusual monomers is very limited. Although some modifications can be applied to the selectable amino acids, this feature is typically restricted to commonly found modifications and does not reflect the wealth of the non-proteinogenic monomers encountered in non-ribosomal peptides. Last but not least, several of the programs mentioned above are web-based tools, showing innate disadvantages as discussed below.

A wealth of knowledge has been accumulated on fragmentation pathways of linear peptides [22]–[26]. Although not equally well understood, the fragmentation of cyclic peptides has also been reasonably well studied and will be described in more detail below. Translation of cyclic peptide fragmentation pathways into a software algorithm should therefore be feasible. To the best of our knowledge, only three software tools capable of *in-silico* fragmentation of cyclic peptides are available for free today.

massXpert2 is a powerful software for polymer processing [38], and suitable for the *in-silico* fragmentation of cyclic peptides, as well. However, its feature-rich user interface can be discouraging for a novice user. In addition, the software requires deep understanding of the fragmentation mechanisms, since the user needs to manually define possible fragmentation pathways.

PFIA was the first software specifically designed for *in-silico* fragmentation of cyclic peptides [39]. It is an open-source software that can be used as an off-line tool. The software is easy to install and the user interface is plain and intuitive. Peptide sequences can be entered and saved. Amino acids can be modified with a restricted set of predefined modifications. A major drawback is that – in addition to only 12 predefined non-proteinogenic amino acids – at most four custom amino acids can be added by the user. This restricts the usability for non-ribosomal peptide analysis, as in many cyclic peptides of this type, more than four unusual amino acids are found. Another disadvantage of the software is that only a-series, b-series and immonium ions can be calculated, while neutral losses and other fragmentation series are not taken into account. Lastly, experimental data cannot be imported, so that automatic matching of calculated fragments to acquired data is not possible.

The most recent software tool, MS-CPA [40], has been refined since its initial publication in 2009, and been renamed to NRP-Annotation (P.A. Pevzner, personnel communication on Sept. 07, 2011). For this program no local installation is possible but it is an exclusively web-based tool, which poses several disadvantages for the user. The program accessibility cannot be guaranteed at all times, as it requires an active Internet connection and a working server hosting the program. The user cannot rely on reproducibility as the software version can be changed by the maintainer without any notice, and previous versions of the program are lost for the user (e.g. the published version of MS-CPA is not available anymore). Raw data of the experiments have to be uploaded to the server, which may hinder scientists to analyze more sensitive data they do not wish to upload to a third party. NRP-Annotation allows for free input of monomer masses to compose a sequence, which enables all imaginable monomers to be entered. However, as the program is a web-based tool, the monomer data has to be entered for each and every use anew. It is not possible to save and reuse entered monomers or sequences for future sessions. This is most inconvenient if e.g. fragmentation patterns for structurally related compounds need to be calculated and all monomer masses of all structures have to be entered individually and repeatedly. The program is not open-source, thus it cannot be altered to suit individual needs. A mass spectrum must be submitted for the calculation, however, only files in the.dta format are accepted, whereas the previous version, MS-CPA, accepted the.mzXML format as well. Moreover, processing of uploaded mass spectra cannot be influenced by the user. After initial processing, calculated fragments are matched to the peaks observed in the spectrum. This results in some statistical data, an annotated spectrum, a list of peaks and matches, and an error plot as output. a-Series and b-series as well as sequence scrambling reactions, neutral losses of water and ammonia, and carbon monoxide adducts are taken into account. It might be confusing that in all reports provided by the software, the mass values of uncharged fragments (i.e. without the mass of any additional charging protons) are used instead of the measured m/z values.

Thus all available software solutions had significant drawbacks and could not be used efficiently for our purpose. The following features have been established to be necessary for the desired annotation tool:

- off-line tool running on multiple operating systems,
- spectrum manipulation and processing abilities (e.g. support for various data formats, baseline correction, peak picking etc.),
- ability to save spectra, sequences and interpretation results for future use,
- monomer database and editor for easy monomer and sequence management,
- inclusion of as many of the known cyclic peptide fragmentation pathways as possible,
- customizable neutral losses allowing for inclusion of side-chain losses,
- allowance of multiple neutral losses from one fragment,
- matching to and annotation of experimental data.

## Materials and Methods

### Compounds and Sample Preparation

**Figure 1:**
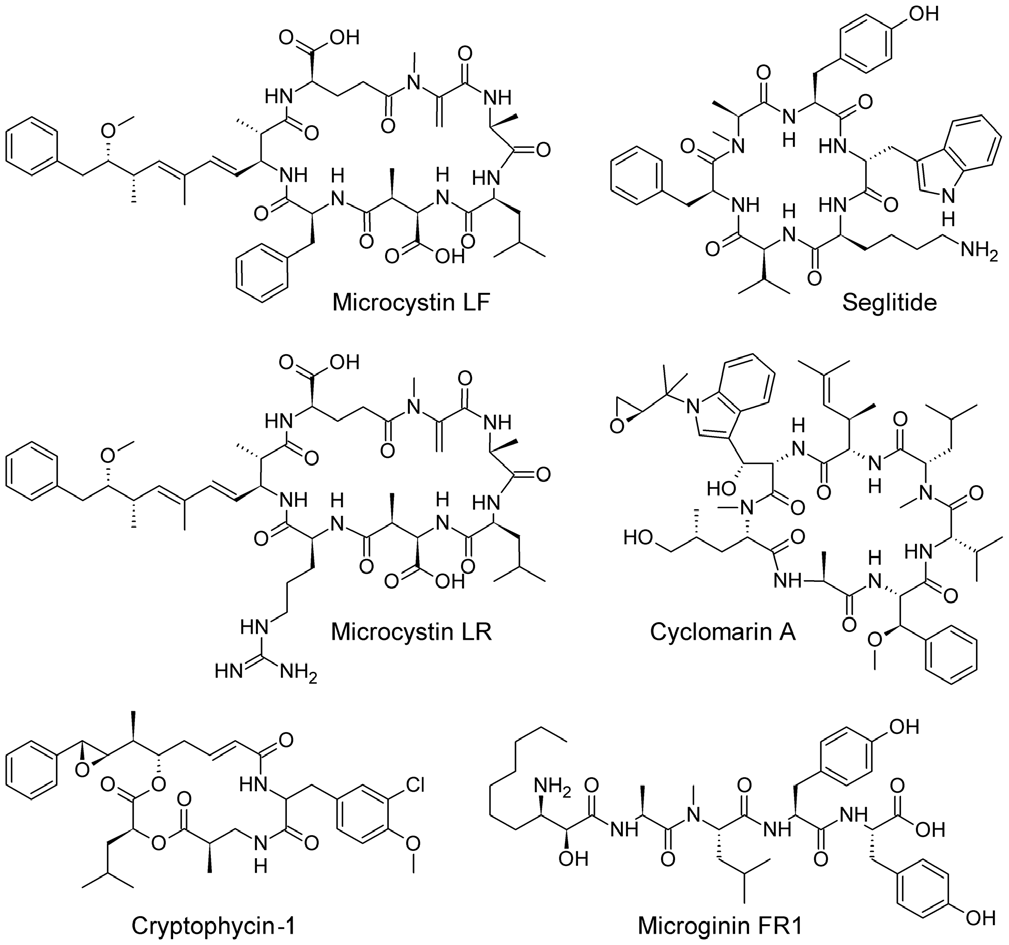
Compounds discussed in this paper.

Microcystin LF, microcystin LR, cryptophycin-1, and microginin FR1, compounds isolated from cyanobacteria, were received from the pure natural products library of Cyano Biotech GmbH, Berlin, Germany. The compounds were dissolved to a concentration of 5 µg/ml in 50∶50 methanol:water. Experimental data for seglitide and cyclomarin A were downloaded from the NRP webpage (http://bix.ucsd.edu/nrp/; IT (auto) data for seglitide, TOF data for cyclomarin A) on September 15, 2011. The structures of the compounds are shown in figure 1.

### Liquid Chromatography – Mass Spectrometry

MS data have been acquired using an HPLC coupled to an IT-TOF mass spectrometer (Shimadzu Europe GmbH, Duisburg, Germany) using electrospray ionization in positive mode and were evaluated using the vendor’s software LCMSSolution version 3.60.361 with Formula Predictor version 1.13. The compounds were separated on a Kinetex C_18_ column (2.6 µm, 100×3 mm, phenomenex, Torrance, USA) using a gradient ranging from 5 to 80% acetonitrile in water over 25 min (0.1% formic acid added as modifier). Precursor ions corresponding to [M+H]^+^ were isolated in the ion trap, fragmented by collision induced dissociation (CID) using argon as collision gas (collision energy set to 150%, collision gas to 100%, and q(Frequency) to 45.0 kHz), and separated in the TOF analyzer. MS/MS scans were averaged and converted to the mzXML format using the vendor’s software. For the calculation of sum formulae, the monoisotopic mass averaged from at least three scans has been used. The Δ values indicated in the text are the deviations of the theoretical masses of the discussed fragments and the experimentally determined masses in ppm.

### Comparison with NRP-Annotation

MS data were imported into mMass and peaks were manually picked. The resulting peak list was saved in the.dta file format and uploaded to the NRP-Annotation server. NRP-Annotation filters peaks according to a non-described algorithm. To ensure a fair comparison between the two software tools, the peak list in mMass was manually reduced to only contain the peaks that were also evaluated by NRP-Annotation.

### Design of the Software and Implemented Fragmentation Pathways

#### Extension of an existing mass spectrometry program

On a search for a software tool satisfying as many as possible of the criteria discussed above, one of the authors (TN) identified the software mMass to be the most suitable basis for further development. mMass is a portable and cross-platform open-source software for mass spectra processing, written mainly in Python [37], which already offered a GUI, spectrum manipulation capabilities, the possibility to enter and save peptide sequences and do *in-silico* fragmentation and spectrum annotation. However, entering and fragmentation of peptides was limited to linear sequences and proteinogenic amino acids, and a user-editable monomer database as well as several fragmentation options important for cyclic peptides were missing. Since mMass is open-source, the software could be modified to meet all of the criteria described above.

First of all, a monomer library editor has been designed, giving the user the possibility to conveniently enter, edit and save monomers needed for the composition of peptide sequences. The library has been filled with all monomers compiled from the NORINE database [16], facilitating its use for new users and allowing for easy addition of own variants. Furthermore, the monomer editor allows for the definition of possible neutral losses from individual monomers.

Secondly, the sequence organization and handling has been extended to enable the composition of peptides containing non-proteinogenic amino acids. For this purpose, an additional sequence editor has been designed that allows the convenient composition of peptides by typing or dragging and dropping monomers from the monomer library right into the editor. In addition, a peptide can be set as being linear or cyclic.

Finally, the fragmentation module has been improved to handle all possible known fragmentation pathways of cyclic peptides, to allow for the loss of custom neutrals, and to allow for multiple neutral losses from one fragment.

To assess the capabilities of the resulting software package, several cyanobacterial natural product MS^2^ spectra have been annotated, and the annotations have been compared to those made by NRP-Annotation.

#### Backbone chain fragmentation pathways

**Figure 2:**
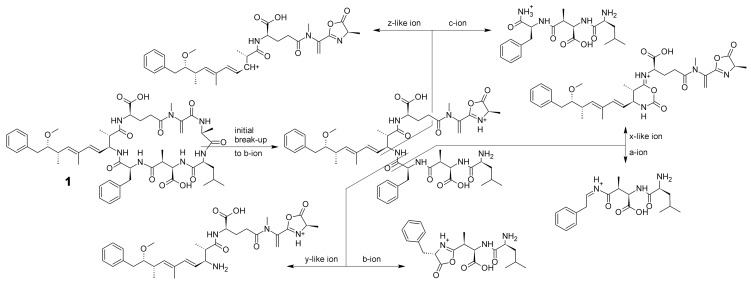
Exemplary backbone fragmentation pathways for a cyclic peptide. After initial cleavage of a random amide bond of Microcystin LF (1), the resulting b-ion can undergo further fragmentation, resulting in a/x-like, b/y-like or c/z-like ions.

Cyclic peptides are known for initial cleavage of the amide bonds, leading to the formation of all possible b-ion linear sequences having a free N-terminus and, as is known for b-ions, an oxazolone “C-terminus” [25], [41], [42]. As all initially formed b-ions have an oxazolone “C-terminus”, x-, y-, and z-ions possibly resulting from further fragmentation of these precursors do not have the free carboxylic acid C-terminus normally found in these ion types. Therefore in this publication, they are called y-like, x-like and z-like ions. A summary of possible backbone fragmentation pathways is depicted in figure 2, taking Microcystin LF as an example.

#### b- and y-like fragment ions

The linear b-ions resulting from the initial cleavage can – as is well known for linear b-ions – undergo further fragmentation via the b_x_-b_x–1_ pathway to form other ions of the respective b-series. Fragmentation can also continue via the b_x_-y_z_ pathway, resulting in the respective y-series [26]. However, as the source fragment of the y-series, like b-ions, has an oxazolone “C-terminus” and not a free carboxylic acid, the y-series ions are not distinguishable from identical b-ions originating from a b_x_-b_x–1_ fragmentation of another linear precursor of the same cyclic peptide. For the sake of completeness, the calculation of y-like ions has been included in mMass, although for every y-like ion there is a structurally corresponding b-ion.

#### a- and x-like fragment ions

b-Ions are known to lose CO to form the corresponding a-ions via the b_x_-a_x_ pathway [26], which is also often described for cyclic peptides [42]–[45]. Formation of x-like ions is mechanistically possible, therefore this ion series has also been included in the software, although to the best of our knowledge these ions have not yet been described for cyclic peptides.

#### c- and z-like fragment ions

c-Type fragmentation of the precursor b-ions is mechanistically possible and has been described for microcystins [46], [47]. The same holds true for z-like fragments [47], which in the case of cyclic peptides are equal to y-like-NH_3_ or b-NH_3_ ions with respect to their sum formula. However, they have been included in the software because they should be brought to the attention of the user also if the options to calculate y-like ions or NH_3_-losses from b-ions are not chosen, and because the NH_3_ loss from y-like or b-ions is not restricted to the N-terminal amino group, whereas in the case of z-like ions, the terminal amino group is always lost.

#### Alternative initial ring opening

**Figure 3:**
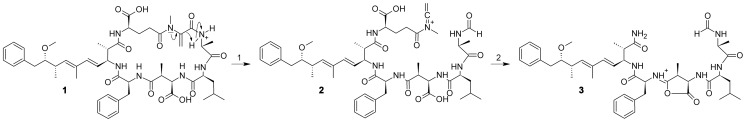
Proposed pathway for the formation of an observed N-formyl fragment ion. Initial D-alanine protonation of microcystin LF (1) and subsequent reactions 1 and 2 result in the formation of the observed N-formyl fragment ion (3). 1) Break-up to N-formyl ion (2). 2) c-like fragmentation and water loss.

b+CO- and c+CO-ions have frequently been described [40], [45], [48], [49]. Although the mechanism of their formation has not yet been extensively studied, Eckart [42] has proposed the following pathway: In addition to the initial formation of b-ions, the cleavage of the C_α_-C_carbonyl_ bond in an a/x-like manner is also possible, resulting in the corresponding N-formyl immonium ions. Subsequent loss of amine fragments leads to ions that formally can be described as “CO adducts”. The proposed mechanism of the formation of a +CO ion observed within this study is shown in figure 3. The formyl group is attached to the N-terminus, thus only a-, b-, and c-type ions can show this formal addition of CO. Addition of CO to an a-type ion leads to an ion with the same sum formula as the corresponding b-ion and can thus not be discriminated in MS^2^ experiments.

#### Sequence scrambling

Sequence scrambling of a- and b-ions is a phenomenon that has recently been described and studied in more detail [50]–[56]. It has also been observed in the case of cyclic peptides [40], [45], [57]. The mechanism of such sequence scrambling according to the cited publications would be as follows. 1) Initial break-up of the cyclic peptide to a b-ion as described above. 2) Fragmentation of the resulting linear peptide. 3) Recyclization of the resulting b- or a-ion fragment by nucleophilic attack of the N-terminal nitrogen on a C-terminal carbonyl carbon in the case of b-ions or on the carbon center of the C-terminal immonium ion in the case of a-ions. 4) Reopening of the resulting cyclic peptide at another peptide bond. 5) Fragmentation of the resulting linear peptide. Sequence scrambling can result in a lot of identical sequences, e.g. for a cyclic peptide with seven monomers the formation of 14 identical b_2_-ions is possible over different pathways. For the sake of clarity, a filtering algorithm has been implemented that removes all ions with identical sequence but the ion formed over the simplest pathway. As is shown in Example S1, the *in-silico* sequence scrambling of YAGFL-NH_2_ using our algorithm fits to the experimental fragmentation described in the literature [51], [58]. The software does not take into account possible sequence scrambling reactions via the side chains [59].

#### Neutral Losses

Loss of H_2_O or NH_3_ is a common phenomenon found for cyclic peptides, even if no hydroxyl groups are present in the molecule [40], [44], [46], [48], [60]–[64]. Loss of H_2_O and NH_3_ at the same time is possible, and also side chain neutral losses in conjunction with H_2_O or NH_3_ losses have been observed [27], [45], [47]. Side chain neutral losses are commonly observed upon Electron Capture Dissociation (ECD), Electron-Transfer Dissociation (ETD) and high-energy Collision Induced Dissociation (CID) [65]–[68]. They are less frequently observed for low-energy CID [23], [69], [70], but several side chain neutral losses have also been described for this dissociation technique, e.g. neutral losses from arginine [47], [71]–[73], the non-proteinogenic amino acid Adda ((2S,3S,8S,9S)-3-amino-9-methoxy-2,6,8-trimethyl-10-phenyl-deca-4,6-dienoic acid) [46], [47] or other modified amino acids [74]. Due to the possible occurrence of these losses, mMass now gives the user the possibility to freely define side chain neutral losses. However, as the authors do not use ECD/ETD, losses typical for these techniques have not been included in the monomer database yet. Users are invited to mine the mentioned references and enter the losses using the build-in monomer editor.

#### Gains

**Figure 4:**
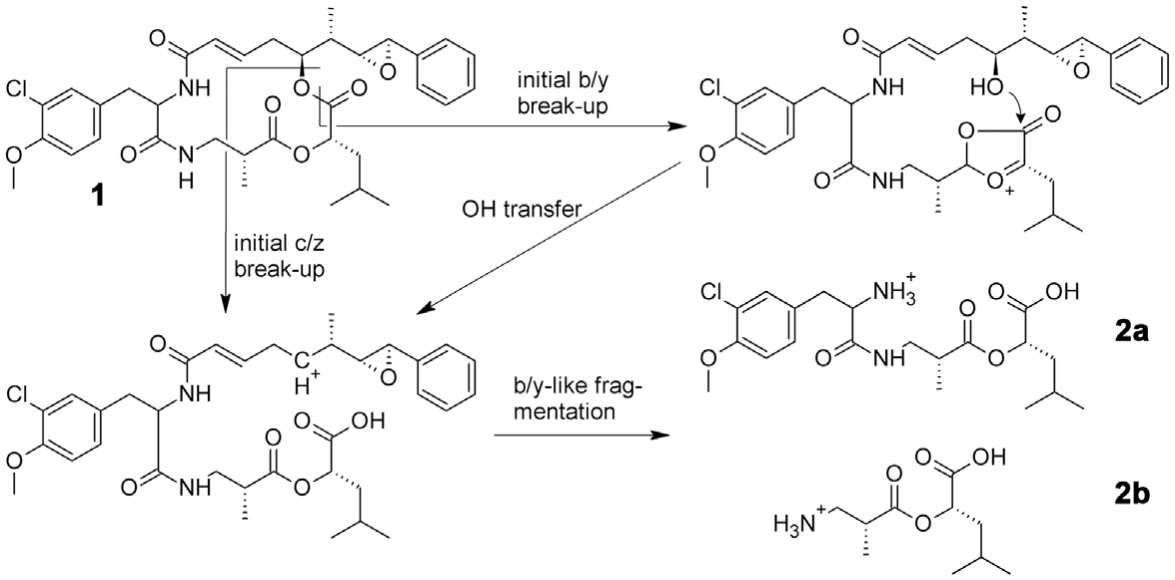
Proposed mechanism for the formation of observed b+H_2_O ions. b+H_2_O fragment ions (2a) and (2b) of cryptophycin-1 (1) can result from two ring opening pathways: direct c/z-like cleavage of the cyclic peptide or b/y-like cleavage followed by OH transfer.

The formal addition of H_2_O to b-ions has been described and mechanistically discussed [57], [73], [75]. Possible pathways for the formation of these ions in the case of cryptophycin-1 are discussed below and shown in Figure 4.

#### Nomenclature

The nomenclature for cyclic peptide fragments proposed by Ngoka and Gross [76], based on Biemanns modification [77] of Roepstorffs nomenclature [78], is suitable for cyclic peptide fragments containing mainly proteinogenic amino acids. However, if a peptide is mainly composed of non-proteinogenic amino acids, the labeling becomes confusing due to multi-letter monomer abbreviations. Thus this software indicates the initial cleavage site using numbers, similar to the approach proposed by Jegorov et al. [79]. Because the software already used the symbol “–” in a different context, we chose to use the symbol “|” instead, optically indicating a bond cleavage between two monomers of the cyclic peptide. Thus e.g. the 6 monomers containing b-ion of a cyclic peptide initially cleaved between the first and second monomer is labeled b_6[1|2]_ _[1]_–_[6]_, 1 being the novel C-terminal oxazolone and 2 the novel N-terminus. Sequence scrambling complicates the nomenclature, as two ring openings and an interjacent fragmentation step must be described. If e.g. the b_6[2|1]_ _[1]_–_[6]_ fragment recyclizes, reopens, is subsequently cleaved after the new position 2 and loses one further amino acid, the fragment is labeled b_5[1|2]_ _[1]_–_[6]__[2|3]_ _[1]_–_[5]_. Thus our fragment ion index nomenclature intuitively summarizes the complete fragment formation history. In the user interface, the complete fragment sequences are given in the fragment table using the respective monomer abbreviations as well.

## Results and Discussion: Evaluation of the Software

### Experimental and *in-silico* Fragmentation of Peptides

**Figure 5:**
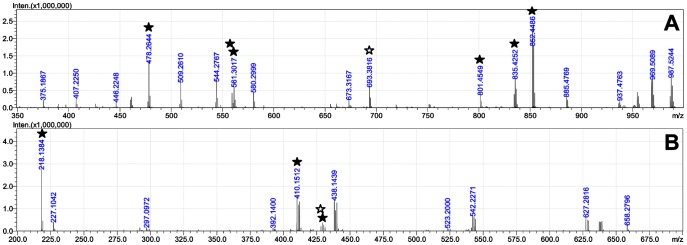
Tandem mass spectra of microcystin LF and cryptophycin-1. (A) microcystin LF, (B) cryptophycin-1. Signals discussed in the text are indicated with a star (filled star: not annotated by NRP-Annotation).

Several compounds were subjected to MS^n^ analyses as described above, and the resulting tandem mass spectra were annotated using both NRP-Annotation and the novel version of mMass. The results of this comparison are described in the following and summarized in table 1. The tandem mass spectra are shown in figure 5. Raw data (Data S1) as well as annotation analysis reports of the compounds discussed in the following (Example S2) can be found in the Supporting Information.

**Table 1:** Comparison of fragment ion annotation using NRP-Annotation or mMass.

	MC-LF^1^	MC-LR^1^	Seglitide^2^	CM A^3^	CRY-1^1^	MG FR1^1^
**NRP-An.**	23%, 13 of 57	52%, 22 of 66	74%, 38 of 53	73%, 36 of 99	32%, 10 of 36	–-
**mMass** *	23%, 13 of 57	52%, 22 of 66	74%, 38 of 53	73%, 36 of 99	32%, 10 of 36	–-
**mMass** **	96%, 51 of 57	79%, 55 of 66	98%, 51 of 53	100%, 90 of 99	80%, 21 of 36***	99%, 12 of 14

Indicated are the percentage of the ion intensity annotated as well as the number of ions matched by the software vs. the total ion number.

MC  =  Microcystin, CM  =  Cyclomarin, CRY  =  Cryptophycin, MG  =  Microginin.

* options chosen that reflect the fragments NRP-Annotation can calculate (a- and b-ions, -NH_3_ and -H_2_O, Allow Scrambling, including b_1_ ions).

** all available options chosen.

*** if ^37^Cl isotope peaks are manually deleted: 88%, 21 of 32.

m/z tolerances used for matching calculated to experimentally observed fragments: ^1^0.015 Da, ^2^0.2 Da, ^3^0.1 Da.

### The Cyclic Peptide Microcystin LF

The fragmentation behavior of microcystins, cyclic heptapeptides found in several cyanobacterial genera, has been well studied [46], [47], [60], [80], [81]. Thus microcystin LF has been used to examine the accuracy and comprehensiveness of our fragmentation algorithms. To our surprise, NRP-Annotation was filtering several significant and important peaks out of the uploaded raw data. Furthermore, only relatively few peaks were annotated (summing up to 23% of the overall peak intensity after filtering). Several of the most abundant signals, including the base peak, were not assigned. Thus we did a comparative study using the novel algorithms of mMass. Including only a- and b-ions, sequence scrambling and simple neutral losses of H_2_O and NH_3_ in our calculations in analogy to NRP-Annotation, we obtained identical assignments. Including also M-, c-, x-, and z-type ions matched an additional 26% of the overall peak intensity. Including the option for N-formyl ions brought a further 10% increase of matched intensity. Allowance of user defined neutral losses, described for the Adda moiety as discussed above, raised the matching quota by further 26%. The inclusion of multiple neutral losses gained 10%, resulting in a total annotated peak intensity of 96%.

The tandem mass spectrum of microcystin LF shows several significant peaks that had not been assigned by NRP-annotation, but were annotated by mMass (figure 5A). The fragment ions observed at m/z 478.2644 and m/z 561.3017 were annotated as c-ions c_4[7|1]_ _[1]_–_[4]_ (Δ 3.3 ppm) and c_5[6|7]_ _[1]_–_[5]_ (Δ 2.5 ppm), resp. A z-like ion with a neutral loss, z_5[2|3]_ _[3]_–_[7]_-C_9_H_10_O, at m/z 559.3127 (Δ 0.2 ppm) is often described for Adda-containing microcystins [47], [60]. The respective ion without neutral loss, z_5[2|3]_ _[3]_–_[7]_ is observed at m/z 693.3816 (also assigned by NRP-Annotation as b-NH_3_-ion; Δ 3.1 ppm). The base peak of the spectrum at m/z 852.4486 has been annotated as M-C_9_H_10_O by mMass (Δ 1.9 ppm), showing how important it is to allow for user definable neutral losses and to include M ions in the calculations. Another significant peak was observed at m/z 835.4252, annotated by mMass as M-C_9_H_10_O-NH_3_ (Δ 1.9 ppm), indicating that fragments with multiple neutral losses can make up a significant proportion of the total peak intensity. N-formyl ions have been assigned to both b- and c-like ion series by mMass; the proposed formation mechanism of a prominent N-formyl c-ion at m/z 801.4549, c_5[7|1]_ [_1]_–_[5]_-H_2_O+CO (Δ 0.5 ppm), is shown in figure 3. To confirm the annotations made, selected ions were subjected to MS^3^. The resulting product ion spectra and their interpretation are presented in Figure S1.

### The Cyclic Depsipeptide Cryptophycin-1

NRP-Annotation again filters many significant peaks out of the uploaded MS^2^ spectrum using its non-described algorithm. The base peak of the spectrum as well as other peaks of significant intensity are not annotated (figure 5B). Analysis by mMass shows that the base peak at m/z 218.1384 is the water adduct of a b-series ion, b_2[2|3]_ _[1]_–_[2]_+H_2_O (Δ 1.4 ppm). In fact, also the water adduct of the fragment ion b_3[1|2]_ [_1]_–_[3]_, containing one more monomer, is found in the spectrum at m/z 429.1782 (Δ 1.2 ppm), although with lower intensity. The free carboxylic acid C-terminus can result from two hypothetic ring opening pathways, either by direct c/z-like cleavage of the cyclic peptide or by b/y-like cleavage followed by OH transfer as proposed by Fang et al. [75] and depicted in figure 4. As all depsipeptides after initial b/y-type cleavage possess a free OH group that can serve as OH donor, or a free carboxylic acid after c/z-type cleavage, we expect that b+H_2_O ions will be commonly observed ions for all depsipeptides. Thus it is important for the interpretation of depsipeptide MS^2^ spectra to have this option available in the software used to annotate the spectra. The third most intense peak at m/z 410.1512 has also not been assigned by NRP-Annotation. mMass assigned this peak as a_2[4|1]_ [_1]_–_[2]_-H_2_O (Δ 1.2 ppm). This underlines the importance to allow neutral losses from all types of initial fragment ions. The corresponding a-ion without water loss is also observed with lower abundance at m/z 428.1628 (Δ 1.4 ppm). The MS^3^ spectra of the main signals discussed and their interpretation are shown in Figure S1.

### Other Cyclic Peptides

Because our assignments of the fragments observed for microcystins LF and cryptophycin-1 using mMass were rather extensive, we also compared the mass spectra annotations of further cyclic peptides, namely microcystin LR, seglitide, and cyclomarin A. For microcystin LR, we again found a more concise annotation using mMass. However, NRP-Annotation did assign a higher percentage of the peak intensity for this compound than for microcystin LF; due to the arginine-localized charge, b-ions containing arginine show a high abundance. The experimental data available for seglitide are low-resolution ion trap data; this leads to a very diverse annotation when using mMass at a peak tolerance of 0.2 Da, resulting in a peak intensity annotation of close to 100%. Data with higher resolution would possibly avoid the ambiguous annotation of the signals, but this data was not available from the NRP-Annotation website. The tandem mass spectrum of seglitide is much easier to interpret than those of the other compounds, because seglitide contains only one non-proteinogenic amino acid. In the case of cyclomarin A, 0.1 Da mass tolerance was used by the authors of MS-CPA although the data had been obtained using a TOF analyzer. Again, mMass was able to annotate almost every peak in the spectrum.

### The Linear Peptide Microginin FR-1

To study the suitability of the software for the *in-silico* fragmentation of linear non-ribosomal peptides, microginin FR-1 served as a model compound. The software efficiently annotated almost all observed peaks. Due to the fact that this compound is a linear peptide, the fragmentation is much simpler and could also have been accomplished with other software suitable for the *in-silico* fragmentation of linear peptides allowing for the definition of custom monomers. However, mMass offers more extensive fragmentation options than most of the other software available and enables very convenient non-ribosomal peptide monomer handling and sequence composition, and thus can also be the software of choice for the annotation of linear peptide tandem mass spectra.

### Conclusions

Because the currently available software tools for cyclic peptide tandem mass spectrum annotation could not be used for comprehensive spectrum annotation, we have significantly extended a freely available and open-source software, mMass, to allow for the necessary functionalities. The program, to the best of our knowledge, is the most concise tool available today for cyclic as well as non-ribosomal peptide tandem mass spectrum annotation, and has proven its usability. Due to the open nature of the software, users are able to contribute. This especially holds true for the monomers library, where individual users are invited to share their own extended monomer databases.

Using own experimental data sets as well as data obtained from other scientists that studied *in-silico* fragmentation of cyclic peptides, we have shown that for all examined peptides, our algorithms were able to assign >80% of the total observed peak intensity and that the software is equally well suited for the annotation of cyclic depsipeptide and linear peptide fragment ions. The assignments made by mMass are more comprehensive than those of all other currently available software tools for this purpose.

mMass is not suitable for the prediction of tandem mass spectra of cyclic peptides as no fragment ion intensities can be calculated. But as the knowledge about the intensity patterns of non-ribosomal peptide fragments, and even more for cyclic peptide fragments, is rudimentary and highly depending on the instrument type used, this is not easy to implement at the time being. Furthermore, the software is not able to check if calculated fragments are likely to be observed under experimental conditions. The resulting annotations thus need the attentive assessment of the analyst – but this holds true for all software solutions. The fragmentation prediction has not been extended yet to also include branched cyclic peptides or peptides with more than one cycle.

In the used datasets, some signals still remained unassigned. A closer examination of these peaks might reveal novel fragmentation mechanisms for cyclic peptides, which can be added in any future version of mMass.

The software can conveniently be used in early steps of structure elucidation/dereplication (matching of *in-silico* fragmentation of compounds in databases with experimental tandem MS spectra) and as one of the last steps of structure confirmation (extent of assignable peaks). A typical workflow for a tandem mass spectrum annotation is presented in Example S3.

### Availability

#### mMass 5.2.0

The Python source code, executables for MS Windows, Mac OS X and Linux as well as test data can be downloaded free of charge from the project webpage http://www.mmass.org. The executables do not require installation. All download packages contain a comprehensive manual.

## Supporting Information

Figure S1 Product ion spectra of selected fragments discussed in the publication.
(PDF)

Data S1 Raw and processed tandem MS data.
(ZIP)

Example S1 Sequence Scrambling using mMass.
(PDF)

Example S2 Analysis and Annotation Reports of the compounds discussed.
(PDF)

Example S3 Workflow for the annotation of mass spectra.
(PDF)
