# Supplementary material for: mMass as a Software Tool for the Annotation of Cyclic Peptide Tandem Mass Spectra
Source: PLoS One. 2012 Sep 13;7(9):e44913. doi: 10.1371/journal.pone.0044913 (PMC3441486; doi:10.1371/journal.pone.0044913)
Supplement: Figure S1 — Product ion spectra of selected fragments discussed in the publication. (PDF) [file pone.0044913.s001.pdf]

Product ion spectra of selected fragments discussed in the publication

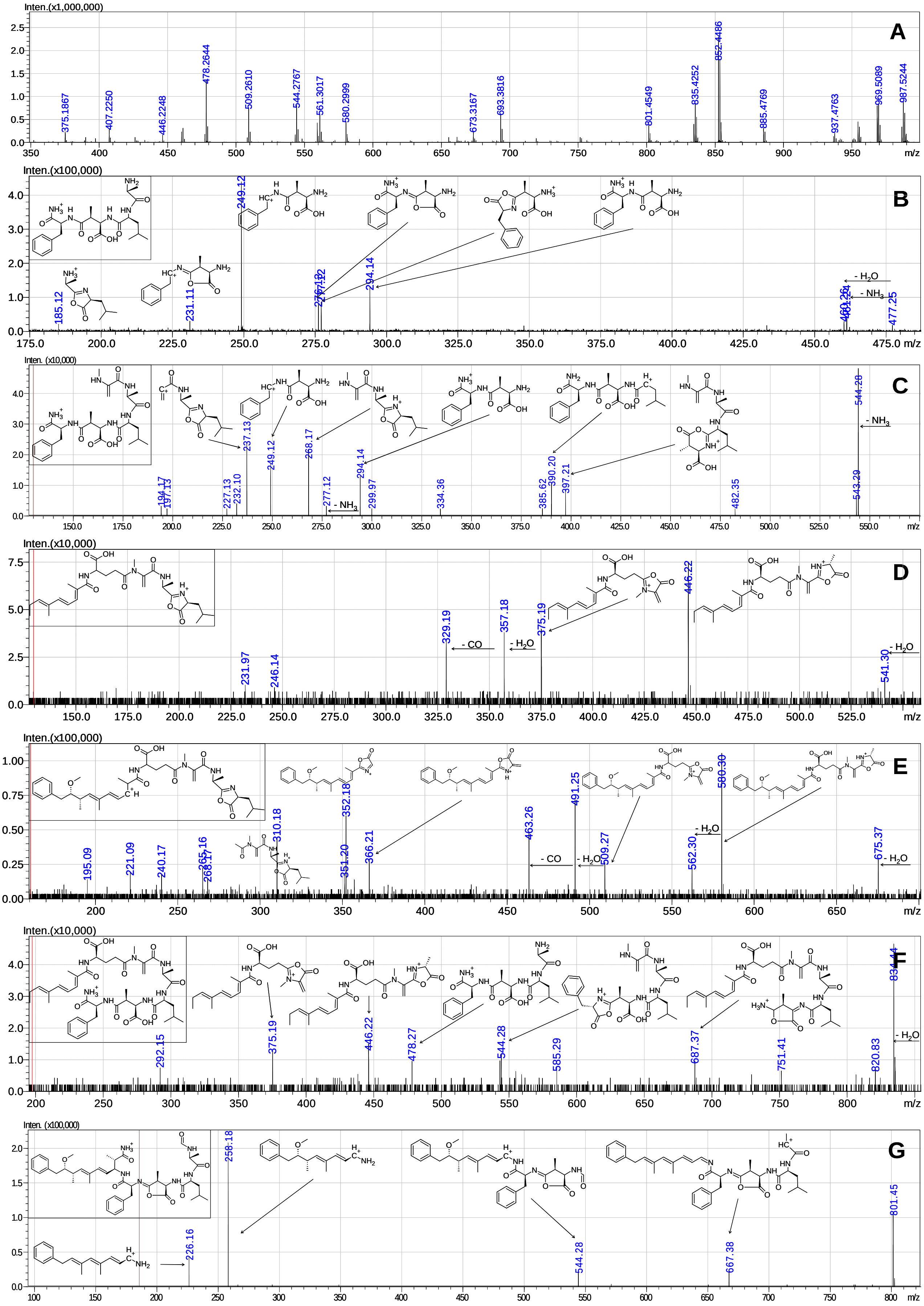

Figure S1a. MS^2 and MS^3 spectra of microcystin LF and their interpretation. **A** Overview of MS/MS spectrum, product ion spectra of the fragments at m/z **B** 478.2644 ($c_{[7|1][1-4]}$), **C** 561.3017 ($c_{[4|5][1-4]}$), **D** 559.3127 ($z_{[2|3][3-7]}$-$C_9H_{10}O$), **E** 693.3816 ($z_{[2|3][3-7]}$), **F** 852.4486 (M-$C_9H_{10}O$), and **G** 801.4549 ($c_{[7|1][1-5]}$-H_2O+CO).

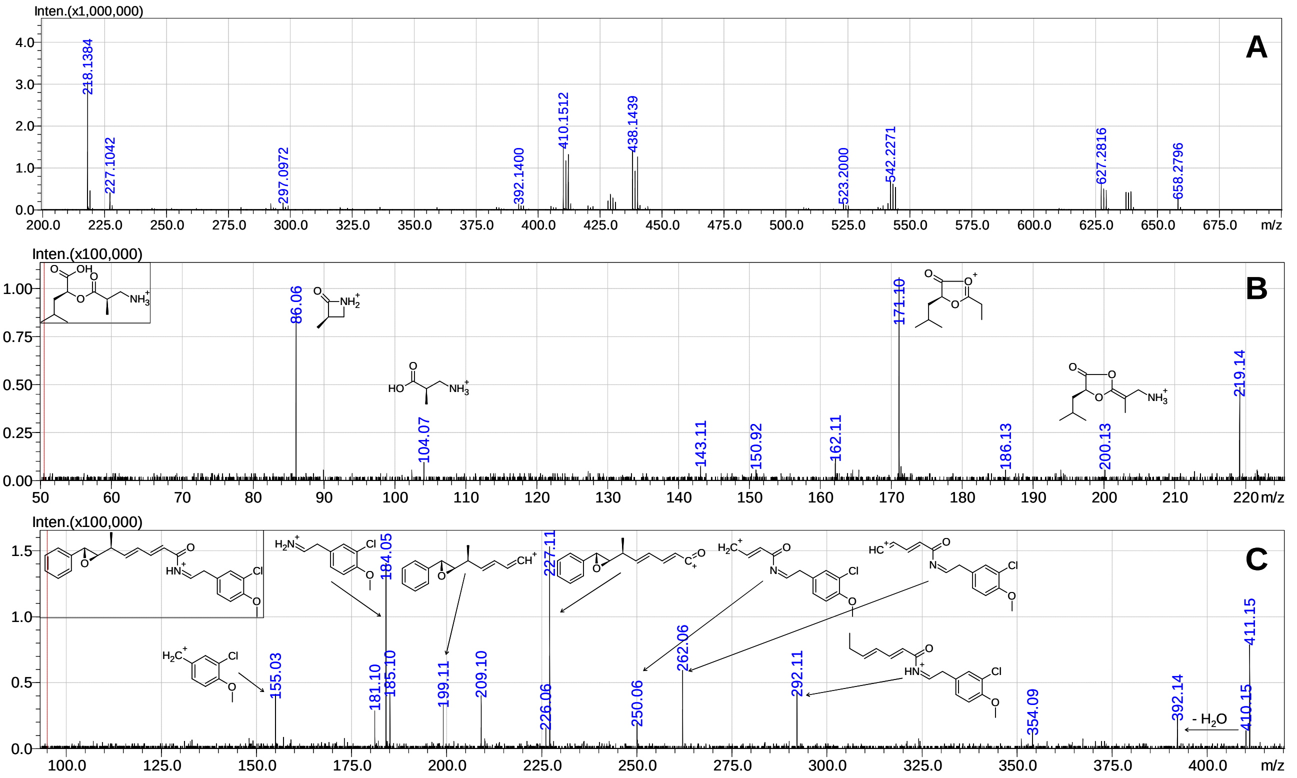

Figure S1b. MS[2] and MS[3] spectra of cryptophycin-1 and their interpretation. **A** Overview of MS/MS spectrum, product ion spectra of the fragments at m/z **B** 218.1384 ($b_{[2|3][1\text{-}2]}+H_2O$) and **C** 410.1512 ($a_{[4|1][1\text{-}2]}-H_2O$).
