# Supplementary material for: mMass as a Software Tool for the Annotation of Cyclic Peptide Tandem Mass Spectra
Source: PLoS One. 2012 Sep 13;7(9):e44913. doi: 10.1371/journal.pone.0044913 (PMC3441486; doi:10.1371/journal.pone.0044913)
Supplement: Example S1 — Sequence Scrambling using mMass. (PDF) [file pone.0044913.s003.pdf]

Sequence scrambling using mMass

As described in the publication, sequence scrambling of peptides can occur as one step during fragmentation. However, in the molecules we have studied, sequence scrambling only plays a very minor role concerning the total peak intensity. It was thus not possible to follow up these minor signals.

Although we did not observe a significant sequence scrambling in our compounds under the experimental conditions used, we have included this option in the software for the sake of completeness – it might well be usable for other compounds than those we have studied to date.

To demonstrate the accuracy of the sequence scrambling algorithm implemented in mMass, below we show a comparison of the experimental spectrum of the linear peptide YAGLF-NH_2 showing sequence scrambling (Figure S1) and the theoretical sequence scrambling signals calculated by mMass (Table S1).

mMass is able to calculate all experimentally observed and manually assigned sequence scrambling signals.

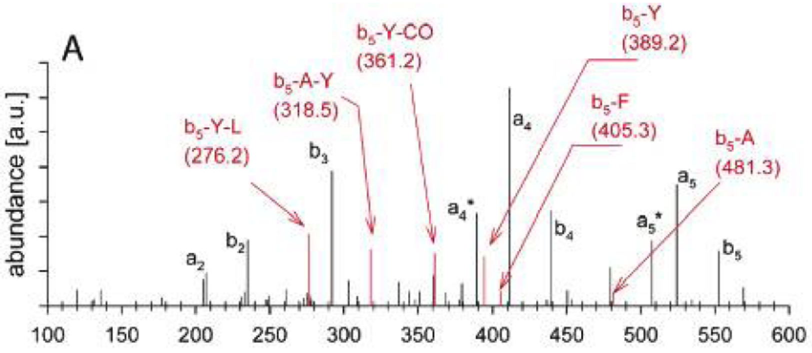

Figure S1. Experimentally observed spectrum of YAGFL-NH_2; manually assigned sequence scrambling b-ions are marked in red. Figure taken from Harrison, A. G.; Young, A. B.; Bleiholder, C.; Suhai, S.; Paizs, B. *J. Am. Chem. Soc.* **2006**, *128*, 10364-10365.

**b3**	**[1-5][1|2][1-3]**	**276.3111**	**.AGF.l**	b3	[1-4][3|4][1-3]	382.4333	.FYA.g
b3 -CO	[1-5][2|3][1-3]	290.3808	.GFL.y	**b4**	**[1-5][1|2][1-4]**	**389.4690**	**.AGFL.y**
**b3**	**[1-5][2|3][1-3]**	**318.3910**	**.GFL.y**	b3 -CO	[1-5][3|4][1-3]	396.5030	.FLY.a
b3 -CO	[1-5][4|5][1-3]	320.4069	.LYA.g	**b4**	**[1-5][4|5][1-4]**	**405.4684**	**.LYAG.f**
b3 -CO	[1-4][2|3][1-3]	340.3966	.GFY.a	b3	[1-5][3|4][1-3]	424.5132	.FLY.a
b3	[1-5][4|5][1-3]	348.4170	.LYA.g	b4 -CO	[1-5][2|3][1-4]	453.5545	.GFLY.a
b3 -CO	[1-4][3|4][1-3]	354.4232	.FYA.g	b4 -CO	[1-5][3|4][1-4]	467.5811	.FLYA.g
**b4 -CO**	**[1-5][1|2][1-4]**	**361.4589**	**.AGFL.y**	**b4**	**[1-5][2|3][1-4]**	**481.5646**	**.GFLY.a**
b3	[1-4][2|3][1-3]	368.4067	.GFY.a	b4	[1-5][3|4][1-4]	495.5912	.FLYA.g
b4 -CO	[1-5][4|5][1-4]	377.4583	.LYAG.f				

Table S1. Summary of all sequence scrambling b-fragments with at least 3 amino acids calculated by mMass; calculated fragments matching experimentally observed fragments are indicated in bold. As the raw data for the tandem MS spectrum of YAGFL-NH_2 have not been available, no further annotations could be done.
