# Supplementary material for: mMass as a Software Tool for the Annotation of Cyclic Peptide Tandem Mass Spectra
Source: PLoS One. 2012 Sep 13;7(9):e44913. doi: 10.1371/journal.pone.0044913 (PMC3441486; doi:10.1371/journal.pone.0044913)
Supplement: Example S2 — Analysis and Annotation Reports of the compounds discussed. (PDF) [file pone.0044913.s004.pdf]

Analysis / Annotation Reports of the compounds discussed

In the following, the analysis / annotation reports for the tandem mass spectra of the six peptides discussed in the manuscript are shown.

Please note: These annotations have been made using ALL fragmentation pathways that are implemented in mMass. The annotations have not been assessed, but are presented here as an unedited table to demonstrate mMass' annotation capabilities. As already mentioned, the software gives **suggestions**, helping the analyst to assign peaks. Critical assessment of the annotations by the analyst is needed after all assignments.

In this context, we want to cite a concern of one of the manuscript reviewers: "If software allows for a wide variety of fragmentation mechanisms in annotation, the number of predicted peaks for a given sequence may rise dramatically. A user unfamiliar with cyclopeptides may believe an ion from an unlikely series was present, even though an expert would reject it as a false positive. What mechanisms can protect naive users from simply turning on all possible ion series and marveling at the matches of impossible ions?"

The best mechanism to avoid false assignments is being aware that false assignments can occur.

Please be careful and do not take all assignments the software makes as true assignments. **If in doubt – leave it out!**

Please also note that using all available fragmentation pathways for the evaluation leads to multiple annotations for many peaks, as of course various fragment ions can have the same sum formula, and thus the same molecular mass.

Microcystin LF	Page 2
Cryptophycin-1	Page 11
Microcystin LR	Page 13
Cyclomarine A	Page 24
Seglitide	Page 37
Microginin FR1	Page 44

mMass Report: *Microcystin LF*

**Date**	Tue Sep 27 13:46:50 2011	**Scan Number**	0
**Operator**	Timo Niedermeyer	**Retention Time**	1055.9
**Contact**	timo.niedermeyer@cyano-biotech.com	**MS Level**	2
**Institution**	Cyano Biotech GmbH	**Precursor m/z**	986.53
**Instrument**	Shimadzu LCMS-IT-TOF ESI IT-TOF	**Polarity**	positive
		**Spectrum Points**	2367
		**Peak List**	56

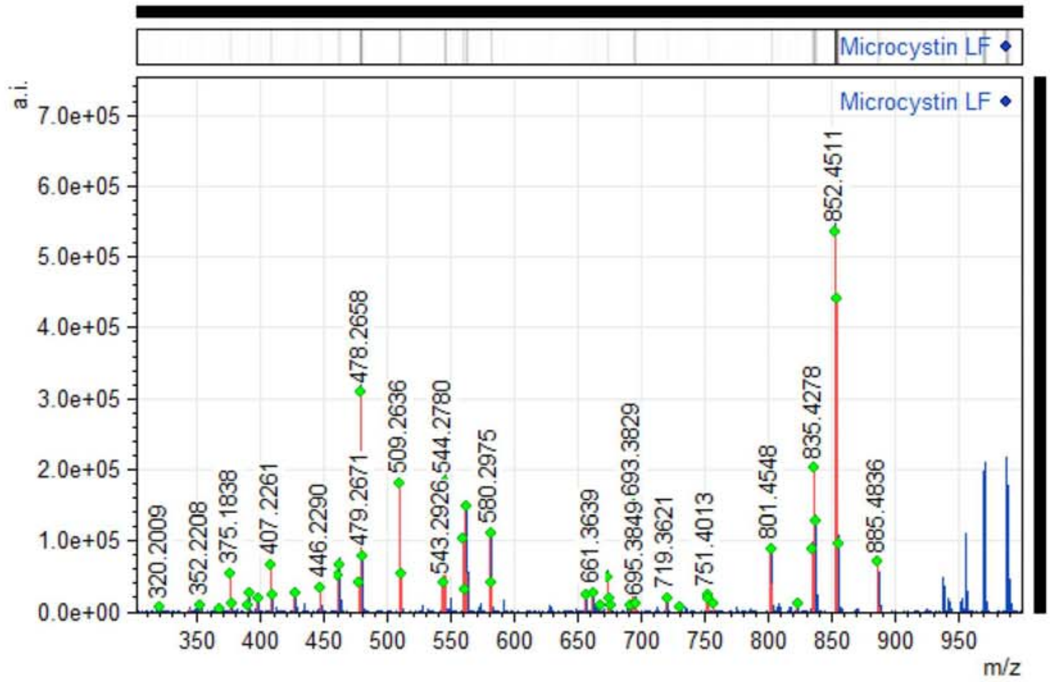

Sequence - *Microcystin LF*

Accession	Length	Mo. Mass	Av. Mass	Coverage	Matched Int.
	7 (Cyclic)	985.5161	986.1621	100.0 %	95.9 %

D-Ala | Leu | D-bMe-Asp | Phe | Adda | D-Glu | NMe-Dha

Meas. m/z	Calc. m/z	δ (Da)	δ (ppm)	Rel. Int. (%)	z	Annotation	Formula
320.2009	320.2094	-0.0085	-26.7	1.21	1	b2 +CO -C9H11O [1|2][1-4][3|4][1-2]	C18H27N2O3
320.2009	320.2094	-0.0085	-26.7	1.21	1	c2 +CO -C9H11O -NH3 [1|2][1-4][3|4][1-2]	C18H27N2O3
320.2009	320.2094	-0.0085	-26.7	1.21	1	b2 +CO -C9H11O [4|5][1-5][4|5][1-2]	C18H27N2O3
320.2009	320.2094	-0.0085	-26.7	1.21	1	c2 +CO -C9H11O -NH3 [4|5][1-5][4|5][1-2]	C18H27N2O3
320.2009	320.2009	0.0000	0.0	1.21	1	a2 -CH3OH -NH3 [4|5][1-3][2|3][1-2]	C22H25NO
320.2009	320.2009	0.0000	0.0	1.21	1	a2 -CH3OH -NH3 [6|7][1-6][5|6][1-2]	C22H25NO
352.2208	352.2271	-0.0064	-18.0	2.01	1	a2 -NH3 [4|5][1-3][2|3][1-2]	C23H29NO2
352.2208	352.2271	-0.0064	-18.0	2.01	1	a2 -NH3 [6|7][1-6][5|6][1-2]	C23H29NO2
352.2208	352.2231	-0.0023	-6.6	2.01	1	a3 -C9H10O [7|1][1-6][4|5][1-3]	C18H29N3O4
352.2208	352.2231	-0.0023	-6.6	2.01	1	a3 -C9H10O [4|5][1-4][3|4][1-3]	C18H29N3O4
352.2208	352.2231	-0.0023	-6.6	2.01	1	b3 +H2O -C9H10O [4|5][1-4][2|3][1-3]	C18H29N3O4
352.2208	352.2231	-0.0023	-6.6	2.01	1	b3 +H2O -C9H10O [6|7][1-6][5|6][1-3]	C18H29N3O4

Meas. m/z	Calc. m/z	δ (Da)	δ (ppm)	Rel. Int. (%)	z	Annotation	Formula
352.2208	352.2145	0.0062	17.7	2.01	1	a3 -C9H11O -NH3 [7|1][1-5][3|4][1-3]	C22H27N2O2
352.2208	352.2145	0.0062	17.7	2.01	1	a3 -C9H11O -NH3 [3|4][1-5][4|5][1-3]	C22H27N2O2
366.2026	366.2064	-0.0038	-10.4	1.02	1	a2 -CH3OH -NH3 [4|5][1-2]	C23H27NO3
366.2026	366.2064	-0.0038	-10.4	1.02	1	a2 -CH3OH -NH3 [2|3][1-3][2|3][1-2]	C23H27NO3
366.2026	366.2064	-0.0038	-10.4	1.02	1	a2 -CH3OH -NH3 [4|5][1-6][5|6][1-2]	C23H27NO3
366.2026	366.2064	-0.0038	-10.4	1.02	1	b2 +H2O -CH3OH -NH3 [4|5][1-3][2|3][1-2]	C23H27NO3
366.2026	366.2064	-0.0038	-10.4	1.02	1	b2 +H2O -CH3OH -NH3 [6|7][1-6][5|6][1-2]	C23H27NO3
375.1838	375.1914	-0.0076	-20.3	10.33	1	z3 -C9H10O [7|1][5-7]	C20H26N2O5
375.1838	375.1914	-0.0076	-20.3	10.33	1	b3 -C9H10O -NH3 [4|5][1-3]	C20H26N2O5
375.1838	375.1829	0.0009	2.5	10.33	1	z3 -C9H11O -NH3 [3|4][1-4][2|3][2-4]	C24H24NO3
375.1838	375.1829	0.0009	2.5	10.33	1	z3 -C9H11O -NH3 [6|7][1-6][1|2][4-6]	C24H24NO3
376.1917	376.1907	0.0010	2.6	2.12	1	x2 -H2O -NH3 [4|5][1-4][1|2][3-4]	C24H25NO3
376.1917	376.1907	0.0010	2.6	2.12	1	z2 -CH3OH -H2O [4|5][1-6][1|2][5-6]	C24H25NO3
376.1917	376.1907	0.0010	2.6	2.12	1	z3 -C9H10O -NH3 [6|7][1-6][1|2][4-6]	C24H25NO3
376.1917	376.1907	0.0010	2.6	2.12	1	b2 +CO -CH3OH -NH3 [4|5][1-3][2|3][1-2]	C24H25NO3
376.1917	376.1907	0.0010	2.6	2.12	1	b2 +CO -CH3OH -NH3 [6|7][1-6][5|6][1-2]	C24H25NO3
376.1917	376.1907	0.0010	2.6	2.12	1	z2 -CH3OH -H2O [6|7][6-7]	C24H25NO3
376.1917	376.1907	0.0010	2.6	2.12	1	x2 -H2O -NH3 [7|1][1-5][1|2][4-5]	C24H25NO3
376.1917	376.1907	0.0010	2.6	2.12	1	z2 -CH3OH -H2O [2|3][1-3][1|2][2-3]	C24H25NO3
376.1917	376.1907	0.0010	2.6	2.12	1	z3 -C9H10O -NH3 [3|4][1-4][2|3][2-4]	C24H25NO3
389.2185	389.2309	-0.0124	-31.8	1.96	1	x3 -C9H11O [7|1][1-5][2|3][3-5]	C21H30N3O4
389.2185	389.2309	-0.0124	-31.8	1.96	1	x3 -C9H11O [4|5][1-5][1|2][3-5]	C21H30N3O4
389.2185	389.2183	0.0002	0.5	1.96	1	c3 -H2O [1|2][1-3]	C20H28N4O4
390.2023	390.2064	-0.0041	-10.5	4.91	1	z2 -H2O -H2O [6|7][6-7]	C25H27NO3
390.2023	390.2064	-0.0041	-10.5	4.91	1	z2 -H2O -H2O [2|3][1-3][1|2][2-3]	C25H27NO3
390.2023	390.2064	-0.0041	-10.5	4.91	1	z2 -H2O -H2O [4|5][1-6][1|2][5-6]	C25H27NO3
390.2023	390.2064	-0.0041	-10.5	4.91	1	b2 +CO -H2O -NH3 [4|5][1-3][2|3][1-2]	C25H27NO3
390.2023	390.2064	-0.0041	-10.5	4.91	1	b2 +CO -H2O -NH3 [6|7][1-6][5|6][1-2]	C25H27NO3
390.2023	390.2023	-0.0001	-0.2	4.91	1	b3 [1|2][1-3]	C20H27N3O5
390.2023	390.2023	-0.0001	-0.2	4.91	1	c3 -NH3 [1|2][1-3]	C20H27N3O5
390.2023	390.2023	-0.0001	-0.2	4.91	1	b3 +CO -C9H10O -H2O [7|1][1-6][4|5][1-3]	C20H27N3O5
390.2023	390.2023	-0.0001	-0.2	4.91	1	b3 +CO -C9H10O -H2O [4|5][1-4][3|4][1-3]	C20H27N3O5
397.2041	397.2082	-0.0041	-10.3	3.89	1	c4 -NH3 [6|7][1-4]	C18H28N4O6
397.2041	397.2082	-0.0041	-10.3	3.89	1	a4 -H2O [7|1][1-6][5|6][1-4]	C18H28N4O6
397.2041	397.2082	-0.0041	-10.3	3.89	1	a4 -H2O [5|6][1-5][2|3][1-4]	C18H28N4O6
397.2041	397.2082	-0.0041	-10.3	3.89	1	b4 [5|6][1-4]	C18H28N4O6
397.2041	397.2082	-0.0041	-10.3	3.89	1	b4 [6|7][1-4]	C18H28N4O6
397.2041	397.2082	-0.0041	-10.3	3.89	1	c4 -NH3 [5|6][1-4]	C18H28N4O6
407.2261	407.2329	-0.0068	-16.7	12.61	1	b2 -H2O -H2O [4|5][1-2]	C25H30N2O3
407.2261	407.2329	-0.0068	-16.7	12.61	1	b2 -H2O -H2O [2|3][1-3][2|3][1-2]	C25H30N2O3
407.2261	407.2329	-0.0068	-16.7	12.61	1	b2 -H2O -H2O [4|5][1-6][5|6][1-2]	C25H30N2O3
407.2261	407.2329	-0.0068	-16.7	12.61	1	b2 +CO -H2O [4|5][1-3][2|3][1-2]	C25H30N2O3
407.2261	407.2329	-0.0068	-16.7	12.61	1	c2 +CO -H2O -NH3 [4|5][1-3][2|3][1-2]	C25H30N2O3
407.2261	407.2329	-0.0068	-16.7	12.61	1	b2 +CO -H2O [6|7][1-6][5|6][1-2]	C25H30N2O3
407.2261	407.2329	-0.0068	-16.7	12.61	1	c2 +CO -H2O -NH3 [6|7][1-6][5|6][1-2]	C25H30N2O3
407.2261	407.2289	-0.0028	-6.8	12.61	1	c3 [1|2][1-3]	C20H30N4O5
407.2261	407.2289	-0.0028	-6.8	12.61	1	c3 +CO -C9H10O -H2O [7|1][1-6][4|5][1-3]	C20H30N4O5
407.2261	407.2289	-0.0028	-6.8	12.61	1	c3 +CO -C9H10O -H2O [4|5][1-4][3|4][1-3]	C20H30N4O5
407.2261	407.2203	0.0058	14.2	12.61	1	b3 +CO -C9H11O -H2O [7|1][1-5][3|4][1-3]	C24H28N3O3
407.2261	407.2203	0.0058	14.2	12.61	1	b3 +CO -C9H11O -H2O [3|4][1-5][4|5][1-3]	C24H28N3O3
408.2295	408.2367	-0.0072	-17.7	4.72	1	c3 -C9H11O [4|5][1-3]	C20H31N4O5
408.2295	408.2282	0.0013	3.3	4.72	1	b3 +CO -C9H10O -H2O [7|1][1-5][3|4][1-3]	C24H29N3O3
408.2295	408.2282	0.0013	3.3	4.72	1	b3 +CO -C9H10O -H2O [3|4][1-5][4|5][1-3]	C24H29N3O3
408.2295	408.2169	0.0126	30.8	4.72	1	b2 +CO -NH3 [6|7][1-6][5|6][1-2]	C25H29NO4

Meas. m/z	Calc. m/z	δ (Da)	δ (ppm)	Rel. Int. (%)	z	Annotation	Formula
408.2295	408.2169	0.0126	30.8	4.72	1	b2 -H2O -NH3 [4|5][1-2]	C25H29NO4
408.2295	408.2169	0.0126	30.8	4.72	1	z2 -H2O [6|7][6-7]	C25H29NO4
408.2295	408.2169	0.0126	30.8	4.72	1	z2 -H2O [2|3][1-3][1|2][2-3]	C25H29NO4
408.2295	408.2169	0.0126	30.8	4.72	1	b2 -H2O -NH3 [2|3][1-3][2|3][1-2]	C25H29NO4
408.2295	408.2169	0.0126	30.8	4.72	1	z2 -H2O [4|5][1-6][1|2][5-6]	C25H29NO4
408.2295	408.2169	0.0126	30.8	4.72	1	b2 -H2O -NH3 [4|5][1-6][5|6][1-2]	C25H29NO4
408.2295	408.2169	0.0126	30.8	4.72	1	b2 +CO -NH3 [4|5][1-3][2|3][1-2]	C25H29NO4
426.2241	426.2387	-0.0147	-34.4	4.99	1	c3 +CO -C9H10O -NH3 [7|1][1-5][3|4][1-3]	C24H31N3O4
426.2241	426.2387	-0.0147	-34.4	4.99	1	b3 +CO -C9H10O [7|1][1-5][3|4][1-3]	C24H31N3O4
426.2241	426.2387	-0.0147	-34.4	4.99	1	b3 +CO -C9H10O [3|4][1-5][4|5][1-3]	C24H31N3O4
426.2241	426.2387	-0.0147	-34.4	4.99	1	c3 +CO -C9H10O -NH3 [3|4][1-5][4|5][1-3]	C24H31N3O4
426.2241	426.2275	-0.0034	-8.1	4.99	1	z2 [4|5][1-6][1|2][5-6]	C25H31NO5
426.2241	426.2275	-0.0034	-8.1	4.99	1	z2 [6|7][6-7]	C25H31NO5
426.2241	426.2275	-0.0034	-8.1	4.99	1	z2 [2|3][1-3][1|2][2-3]	C25H31NO5
426.2241	426.2275	-0.0034	-8.1	4.99	1	b2 -NH3 [4|5][1-2]	C25H31NO5
426.2241	426.2275	-0.0034	-8.1	4.99	1	b2 -NH3 [4|5][1-6][5|6][1-2]	C25H31NO5
426.2241	426.2275	-0.0034	-8.1	4.99	1	b2 -NH3 [2|3][1-3][2|3][1-2]	C25H31NO5
446.2290	446.2438	-0.0149	-33.3	6.56	1	z4 -C9H10O -H2O [6|7][1-6][2|3][3-6]	C27H31N3O3
446.2290	446.2438	-0.0149	-33.3	6.56	1	z4 -C9H10O -H2O [3|4][1-5][2|3][2-5]	C27H31N3O3
446.2290	446.2438	-0.0149	-33.3	6.56	1	b3 +CO -CH3OH -H2O [4|5][1-4][2|3][1-3]	C27H31N3O3
446.2290	446.2438	-0.0149	-33.3	6.56	1	b3 +CO -CH3OH -H2O [6|7][1-6][5|6][1-3]	C27H31N3O3
446.2290	446.2286	0.0004	0.9	6.56	1	z4 -C9H10O [1|2][4-7]	C23H31N3O6
446.2290	446.2286	0.0004	0.9	6.56	1	b4 -C9H10O -NH3 [4|5][1-4]	C23H31N3O6
446.2290	446.2200	0.0089	20.1	6.56	1	z4 -C9H11O -NH3 [3|4][1-5][2|3][2-5]	C27H29N2O4
446.2290	446.2200	0.0089	20.1	6.56	1	z4 -C9H11O -NH3 [6|7][1-6][2|3][3-6]	C27H29N2O4
460.2549	460.2554	-0.0005	-1.1	9.92	1	c4 +CO [3|4][1-6][3|4][1-4]	C23H33N5O5
460.2549	460.2554	-0.0005	-1.1	9.92	1	c4 +CO [6|7][1-5][4|5][1-4]	C23H33N5O5
460.2549	460.2554	-0.0005	-1.1	9.92	1	c4 -H2O [7|1][1-4]	C23H33N5O5
460.2549	460.2402	0.0147	32.0	9.92	1	c4 [5|6][1-5][2|3][1-4]	C19H33N5O8
460.2549	460.2402	0.0147	32.0	9.92	1	c4 [7|1][1-6][5|6][1-4]	C19H33N5O8
461.2477	461.2435	0.0042	9.2	12.69	1	z3 -H2O -H2O [7|1][1-6][1|2][4-6]	C28H32N2O4
461.2477	461.2435	0.0042	9.2	12.69	1	z3 -H2O -H2O [4|5][1-4][2|3][2-4]	C28H32N2O4
461.2477	461.2435	0.0042	9.2	12.69	1	b3 +CO -H2O -NH3 [4|5][1-4][2|3][1-3]	C28H32N2O4
461.2477	461.2435	0.0042	9.2	12.69	1	b3 +CO -H2O -NH3 [6|7][1-6][5|6][1-3]	C28H32N2O4
461.2477	461.2395	0.0083	17.9	12.69	1	c4 -NH3 [7|1][1-4]	C23H32N4O6
461.2477	461.2395	0.0083	17.9	12.69	1	b4 [7|1][1-4]	C23H32N4O6
477.2441	477.2537	-0.0096	-20.1	7.95	1	z3 -CH3OH -H2O [3|4][1-4][2|3][2-4]	C32H32N2O2
477.2441	477.2537	-0.0096	-20.1	7.95	1	z3 -CH3OH -H2O [6|7][1-6][1|2][4-6]	C32H32N2O2
477.2441	477.2384	0.0057	11.9	7.95	1	z3 -CH3OH [7|1][5-7]	C28H32N2O5
477.2441	477.2384	0.0057	11.9	7.95	1	b3 -CH3OH -NH3 [4|5][1-3]	C28H32N2O5
477.2441	477.2384	0.0057	11.9	7.95	1	x3 -NH3 [4|5][1-4][1|2][2-4]	C28H32N2O5
477.2441	477.2384	0.0057	11.9	7.95	1	x3 -NH3 [6|7][1-6][2|3][4-6]	C28H32N2O5
478.2658	478.2700	-0.0042	-8.8	57.54	1	b3 -H2O -H2O [7|1][1-6][4|5][1-3]	C28H35N3O4
478.2658	478.2700	-0.0042	-8.8	57.54	1	b3 -H2O -H2O [4|5][1-4][3|4][1-3]	C28H35N3O4
478.2658	478.2700	-0.0042	-8.8	57.54	1	b3 +CO -H2O [4|5][1-4][2|3][1-3]	C28H35N3O4
478.2658	478.2700	-0.0042	-8.8	57.54	1	c3 +CO -H2O -NH3 [4|5][1-4][2|3][1-3]	C28H35N3O4
478.2658	478.2700	-0.0042	-8.8	57.54	1	b3 +CO -H2O [6|7][1-6][5|6][1-3]	C28H35N3O4
478.2658	478.2700	-0.0042	-8.8	57.54	1	c3 +CO -H2O -NH3 [6|7][1-6][5|6][1-3]	C28H35N3O4
478.2658	478.2660	-0.0002	-0.4	57.54	1	c4 [7|1][1-4]	C23H35N5O6
479.2671	479.2738	-0.0067	-14.0	14.80	1	c4 -C9H11O [4|5][1-4]	C23H36N5O6
479.2671	479.2540	0.0131	27.3	14.80	1	z3 -H2O [7|1][1-6][1|2][4-6]	C28H34N2O5
479.2671	479.2540	0.0131	27.3	14.80	1	b3 -H2O -NH3 [7|1][1-6][4|5][1-3]	C28H34N2O5
479.2671	479.2540	0.0131	27.3	14.80	1	z3 -H2O [4|5][1-4][2|3][2-4]	C28H34N2O5
479.2671	479.2540	0.0131	27.3	14.80	1	b3 -H2O -NH3 [4|5][1-4][3|4][1-3]	C28H34N2O5

Meas. m/z	Calc. m/z	δ (Da)	δ (ppm)	Rel. Int. (%)	z	Annotation	Formula
479.2671	479.2540	0.0131	27.3	14.80	1	b3 +CO -NH3 [4|5][1-4][2|3][1-3]	C28H34N2O5
479.2671	479.2540	0.0131	27.3	14.80	1	b3 +CO -NH3 [6|7][1-6][5|6][1-3]	C28H34N2O5
509.2636	509.2758	-0.0122	-23.9	33.85	1	b4 -C9H10O -H2O [7|1][1-5][2|3][1-4]	C28H36N4O5
509.2636	509.2758	-0.0122	-23.9	33.85	1	b4 -C9H10O -H2O [7|1][1-6][3|4][1-4]	C28H36N4O5
509.2636	509.2758	-0.0122	-23.9	33.85	1	b4 -C9H10O -H2O [3|4][1-5][4|5][1-4]	C28H36N4O5
509.2636	509.2758	-0.0122	-23.9	33.85	1	b4 -C9H10O -H2O [2|3][1-6][5|6][1-4]	C28H36N4O5
509.2636	509.2758	-0.0122	-23.9	33.85	1	c3 +CO -CH3OH -H2O [7|1][1-6][4|5][1-3]	C28H36N4O5
509.2636	509.2758	-0.0122	-23.9	33.85	1	b4 +CO -C9H10O [6|7][1-6][4|5][1-4]	C28H36N4O5
509.2636	509.2758	-0.0122	-23.9	33.85	1	b4 +CO -C9H10O [3|4][1-5][3|4][1-4]	C28H36N4O5
509.2636	509.2758	-0.0122	-23.9	33.85	1	c4 +CO -C9H10O -NH3 [3|4][1-5][3|4][1-4]	C28H36N4O5
509.2636	509.2758	-0.0122	-23.9	33.85	1	c3 +CO -CH3OH -H2O [4|5][1-4][3|4][1-3]	C28H36N4O5
509.2636	509.2758	-0.0122	-23.9	33.85	1	c4 +CO -C9H10O -NH3 [6|7][1-6][4|5][1-4]	C28H36N4O5
509.2636	509.2646	-0.0010	-1.9	33.85	1	z3 [7|1][5-7]	C29H36N2O6
509.2636	509.2646	-0.0010	-1.9	33.85	1	b3 -NH3 [4|5][1-3]	C29H36N2O6
509.2636	509.2646	-0.0010	-1.9	33.85	1	a3 -H2O -NH3 [2|3][1-4][2|3][1-3]	C29H36N2O6
509.2636	509.2646	-0.0010	-1.9	33.85	1	a3 -H2O -NH3 [4|5][1-6][5|6][1-3]	C29H36N2O6
509.2636	509.2520	0.0116	22.8	33.85	1	z4 -C9H11O [7|1][1-5][1|2][2-5]	C28H34N3O6
509.2636	509.2520	0.0116	22.8	33.85	1	z4 -C9H11O [7|1][1-6][1|2][3-6]	C28H34N3O6
509.2636	509.2520	0.0116	22.8	33.85	1	b4 -C9H11O -NH3 [7|1][1-5][2|3][1-4]	C28H34N3O6
509.2636	509.2520	0.0116	22.8	33.85	1	b4 -C9H11O -NH3 [7|1][1-6][3|4][1-4]	C28H34N3O6
509.2636	509.2520	0.0116	22.8	33.85	1	z4 -C9H11O [2|3][1-6][3|4][3-6]	C28H34N3O6
509.2636	509.2520	0.0116	22.8	33.85	1	b4 -C9H11O -NH3 [2|3][1-6][5|6][1-4]	C28H34N3O6
509.2636	509.2520	0.0116	22.8	33.85	1	z4 -C9H11O [3|4][1-5][3|4][2-5]	C28H34N3O6
509.2636	509.2520	0.0116	22.8	33.85	1	b4 -C9H11O -NH3 [3|4][1-5][4|5][1-4]	C28H34N3O6
510.2649	510.2751	-0.0102	-20.1	10.20	1	b3 +CO -CH3OH -H2O [7|1][1-5][3|4][1-3]	C32H35N3O3
510.2649	510.2751	-0.0102	-20.1	10.20	1	b3 +CO -CH3OH -H2O [3|4][1-5][4|5][1-3]	C32H35N3O3
510.2649	510.2639	0.0010	1.9	10.20	1	z3 -NH3 [3|4][1-4][2|3][2-4]	C33H35NO4
510.2649	510.2639	0.0010	1.9	10.20	1	z3 -NH3 [6|7][1-6][1|2][4-6]	C33H35NO4
510.2649	510.2599	0.0050	9.8	10.20	1	z4 -C9H10O [2|3][1-6][3|4][3-6]	C28H35N3O6
510.2649	510.2599	0.0050	9.8	10.20	1	b4 -C9H10O -NH3 [2|3][1-6][5|6][1-4]	C28H35N3O6
510.2649	510.2599	0.0050	9.8	10.20	1	z4 -C9H10O [3|4][1-5][3|4][2-5]	C28H35N3O6
510.2649	510.2599	0.0050	9.8	10.20	1	b4 -C9H10O -NH3 [3|4][1-5][4|5][1-4]	C28H35N3O6
510.2649	510.2599	0.0050	9.8	10.20	1	b3 +CO -CH3OH [7|1][1-6][4|5][1-3]	C28H35N3O6
510.2649	510.2599	0.0050	9.8	10.20	1	c3 +CO -CH3OH -NH3 [7|1][1-6][4|5][1-3]	C28H35N3O6
510.2649	510.2599	0.0050	9.8	10.20	1	b3 +CO -CH3OH [4|5][1-4][3|4][1-3]	C28H35N3O6
510.2649	510.2599	0.0050	9.8	10.20	1	c3 +CO -CH3OH -NH3 [4|5][1-4][3|4][1-3]	C28H35N3O6
510.2649	510.2599	0.0050	9.8	10.20	1	b4 -C9H10O -NH3 [7|1][1-6][3|4][1-4]	C28H35N3O6
510.2649	510.2599	0.0050	9.8	10.20	1	z4 -C9H10O [7|1][1-5][1|2][2-5]	C28H35N3O6
510.2649	510.2599	0.0050	9.8	10.20	1	b4 -C9H10O -NH3 [7|1][1-5][2|3][1-4]	C28H35N3O6
510.2649	510.2599	0.0050	9.8	10.20	1	z4 -C9H10O [7|1][1-6][1|2][3-6]	C28H35N3O6
543.2926	543.3051	-0.0125	-23.1	7.71	1	c4 -C9H11O [7|1][1-5][2|3][1-4]	C28H40N5O6
543.2926	543.3051	-0.0125	-23.1	7.71	1	c4 -C9H11O [7|1][1-6][3|4][1-4]	C28H40N5O6
543.2926	543.3051	-0.0125	-23.1	7.71	1	c4 -C9H11O [2|3][1-6][5|6][1-4]	C28H40N5O6
543.2926	543.3051	-0.0125	-23.1	7.71	1	c4 -C9H11O [3|4][1-5][4|5][1-4]	C28H40N5O6
543.2926	543.2926	0.0001	0.1	7.71	1	c5 -H2O [6|7][1-5]	C27H38N6O6
543.2926	543.2926	0.0001	0.1	7.71	1	c5 -H2O [3|4][1-6][2|3][1-5]	C27H38N6O6
543.2926	543.2926	0.0001	0.1	7.71	1	c5 -H2O [5|6][1-6][5|6][1-5]	C27H38N6O6
543.2926	543.2853	0.0073	13.4	7.71	1	b3 +CO -NH3 [7|1][1-5][3|4][1-3]	C33H38N2O5
543.2926	543.2853	0.0073	13.4	7.71	1	b3 +CO -NH3 [3|4][1-5][4|5][1-3]	C33H38N2O5
544.2780	544.2892	-0.0112	-20.5	34.07	1	b4 +H2O -C9H11O [7|1][1-5][2|3][1-4]	C28H39N4O7
544.2780	544.2892	-0.0112	-20.5	34.07	1	b4 +H2O -C9H11O [7|1][1-6][3|4][1-4]	C28H39N4O7
544.2780	544.2892	-0.0112	-20.5	34.07	1	b4 +H2O -C9H11O [2|3][1-6][5|6][1-4]	C28H39N4O7
544.2780	544.2892	-0.0112	-20.5	34.07	1	b4 +H2O -C9H11O [3|4][1-5][4|5][1-4]	C28H39N4O7
544.2780	544.2806	-0.0026	-4.8	34.07	1	z4 -H2O -H2O [1|2][4-7]	C32H37N3O5

Meas. m/z	Calc. m/z	δ (Da)	δ (ppm)	Rel. Int. (%)	z	Annotation	Formula
544.2780	544.2766	0.0014	2.6	34.07	1	c5 -NH3 [6|7][1-5]	C27H37N5O7
544.2780	544.2766	0.0014	2.6	34.07	1	a5 -H2O [7|1][1-6][5|6][1-5]	C27H37N5O7
544.2780	544.2766	0.0014	2.6	34.07	1	c5 -NH3 [3|4][1-6][2|3][1-5]	C27H37N5O7
544.2780	544.2766	0.0014	2.6	34.07	1	a5 -H2O [5|6][1-6][2|3][1-5]	C27H37N5O7
544.2780	544.2766	0.0014	2.6	34.07	1	c5 -NH3 [5|6][1-6][5|6][1-5]	C27H37N5O7
544.2780	544.2766	0.0014	2.6	34.07	1	b5 [6|7][1-5]	C27H37N5O7
544.2780	544.2766	0.0014	2.6	34.07	1	b5 [3|4][1-6][2|3][1-5]	C27H37N5O7
544.2780	544.2766	0.0014	2.6	34.07	1	b5 [5|6][1-6][5|6][1-5]	C27H37N5O7
545.2780	545.2844	-0.0064	-11.7	12.34	1	a5 -C9H11O -H2O [2|3][1-6][2|3][1-5]	C27H38N5O7
545.2780	545.2844	-0.0064	-11.7	12.34	1	a5 -C9H11O -H2O [4|5][1-6][5|6][1-5]	C27H38N5O7
545.2780	545.2646	0.0134	24.6	12.34	1	z4 -H2O -NH3 [1|2][4-7]	C32H36N2O6
559.3139	559.3279	-0.0140	-25.0	19.24	1	b4 +CO -CH3OH -H2O [4|5][1-5][2|3][1-4]	C33H42N4O4
559.3139	559.3279	-0.0140	-25.0	19.24	1	c3 +CO -H2O [3|4][1-5][4|5][1-3]	C33H42N4O4
559.3139	559.3279	-0.0140	-25.0	19.24	1	b4 +CO -CH3OH -H2O [6|7][1-6][5|6][1-4]	C33H42N4O4
559.3139	559.3279	-0.0140	-25.0	19.24	1	z5 -C9H10O -H2O [3|4][1-6][2|3][2-6]	C33H42N4O4
559.3139	559.3279	-0.0140	-25.0	19.24	1	z5 -C9H10O -H2O [6|7][1-6][3|4][2-6]	C33H42N4O4
559.3139	559.3279	-0.0140	-25.0	19.24	1	c3 +CO -H2O [7|1][1-5][3|4][1-3]	C33H42N4O4
559.3139	559.3126	0.0013	2.3	19.24	1	z5 -C9H10O [2|3][3-7]	C29H42N4O7
559.3139	559.3126	0.0013	2.3	19.24	1	b5 -C9H10O -NH3 [4|5][1-5]	C29H42N4O7
559.3139	559.3126	0.0013	2.3	19.24	1	z5 -C9H10O [4|5][1-6][1|2][2-6]	C29H42N4O7
559.3139	559.3126	0.0013	2.3	19.24	1	b5 -C9H10O -NH3 [4|5][1-6][2|3][1-5]	C29H42N4O7
559.3139	559.3126	0.0013	2.3	19.24	1	z5 -C9H10O [6|7][1-6][4|5][2-6]	C29H42N4O7
559.3139	559.3126	0.0013	2.3	19.24	1	b5 -C9H10O -NH3 [6|7][1-6][5|6][1-5]	C29H42N4O7
559.3139	559.3126	0.0013	2.3	19.24	1	c3 +CO [7|1][1-6][4|5][1-3]	C29H42N4O7
559.3139	559.3126	0.0013	2.3	19.24	1	c3 +CO [4|5][1-4][3|4][1-3]	C29H42N4O7
559.3139	559.3041	0.0098	17.6	19.24	1	z5 -C9H11O -NH3 [3|4][1-6][2|3][2-6]	C33H40N3O5
559.3139	559.3041	0.0098	17.6	19.24	1	z5 -C9H11O -NH3 [6|7][1-6][3|4][2-6]	C33H40N3O5
560.3149	560.3119	0.0030	5.3	6.22	1	b4 +CO -CH3OH -NH3 [6|7][1-6][5|6][1-4]	C33H41N3O5
560.3149	560.3119	0.0030	5.3	6.22	1	z4 -CH3OH -H2O [4|5][1-6][1|2][3-6]	C33H41N3O5
560.3149	560.3119	0.0030	5.3	6.22	1	z4 -CH3OH -H2O [7|1][1-5][3|4][2-5]	C33H41N3O5
560.3149	560.3119	0.0030	5.3	6.22	1	z4 -CH3OH -H2O [7|1][1-6][2|3][3-6]	C33H41N3O5
560.3149	560.3119	0.0030	5.3	6.22	1	z5 -C9H10O -NH3 [3|4][1-6][2|3][2-6]	C33H41N3O5
560.3149	560.3119	0.0030	5.3	6.22	1	z4 -CH3OH -H2O [4|5][1-5][2|3][2-5]	C33H41N3O5
560.3149	560.3119	0.0030	5.3	6.22	1	z5 -C9H10O -NH3 [6|7][1-6][3|4][2-6]	C33H41N3O5
560.3149	560.3119	0.0030	5.3	6.22	1	b3 +CO [7|1][1-5][3|4][1-3]	C33H41N3O5
560.3149	560.3119	0.0030	5.3	6.22	1	b3 +CO [3|4][1-5][4|5][1-3]	C33H41N3O5
560.3149	560.3119	0.0030	5.3	6.22	1	c3 +CO -NH3 [7|1][1-5][3|4][1-3]	C33H41N3O5
560.3149	560.3119	0.0030	5.3	6.22	1	c3 +CO -NH3 [3|4][1-5][4|5][1-3]	C33H41N3O5
560.3149	560.3119	0.0030	5.3	6.22	1	b4 +CO -CH3OH -NH3 [4|5][1-5][2|3][1-4]	C33H41N3O5
561.3034	561.3071	-0.0037	-6.6	27.76	1	b4 -H2O -H2O [4|5][1-4]	C32H40N4O5
561.3034	561.3031	0.0003	0.6	27.76	1	c5 [6|7][1-5]	C27H40N6O7
561.3034	561.3031	0.0003	0.6	27.76	1	c5 [3|4][1-6][2|3][1-5]	C27H40N6O7
561.3034	561.3031	0.0003	0.6	27.76	1	c5 [5|6][1-6][5|6][1-5]	C27H40N6O7
561.3034	561.2959	0.0075	13.4	27.76	1	z4 -CH3OH -NH3 [4|5][1-5][2|3][2-5]	C33H40N2O6
561.3034	561.2959	0.0075	13.4	27.76	1	z4 -CH3OH -NH3 [4|5][1-6][1|2][3-6]	C33H40N2O6
561.3034	561.2959	0.0075	13.4	27.76	1	z4 -CH3OH -NH3 [7|1][1-5][3|4][2-5]	C33H40N2O6
561.3034	561.2959	0.0075	13.4	27.76	1	z4 -CH3OH -NH3 [7|1][1-6][2|3][3-6]	C33H40N2O6
561.3034	561.2919	0.0116	20.6	27.76	1	b4 +CO -C9H10O -H2O [4|5][1-6][4|5][1-4]	C28H40N4O8
561.3034	561.2919	0.0116	20.6	27.76	1	b4 +CO -C9H10O -H2O [1|2][1-5][3|4][1-4]	C28H40N4O8
580.2975	580.3017	-0.0042	-7.3	20.98	1	z4 [1|2][4-7]	C32H41N3O7
580.2975	580.3017	-0.0042	-7.3	20.98	1	b4 -NH3 [4|5][1-4]	C32H41N3O7
580.2975	580.3017	-0.0042	-7.3	20.98	1	b4 +CO -C9H10O -NH3 [1|2][1-4]	C32H41N3O7
580.2975	580.3017	-0.0042	-7.3	20.98	1	b4 +CO -C9H10O -NH3 [1|2][1-5][2|3][1-4]	C32H41N3O7
580.2975	580.3017	-0.0042	-7.3	20.98	1	b4 +CO -C9H10O -NH3 [3|4][1-6][5|6][1-4]	C32H41N3O7

Meas. m/z	Calc. m/z	δ (Da)	δ (ppm)	Rel. Int. (%)	z	Annotation	Formula
581.3040	581.3010	0.0030	5.1	7.84	1	z4 -NH3 [3|4][1-5][2|3][2-5]	C36H40N2O5
581.3040	581.3010	0.0030	5.1	7.84	1	z4 -NH3 [6|7][1-6][2|3][3-6]	C36H40N2O5
581.3040	581.2970	0.0070	12.1	7.84	1	c3 +CO -H2O -H2O [2|3][1-4][2|3][1-3]	C31H40N4O7
581.3040	581.2970	0.0070	12.1	7.84	1	c3 +CO -H2O -H2O [4|5][1-6][5|6][1-3]	C31H40N4O7
655.3062	655.3212	-0.0150	-22.9	4.61	1	b5 -C9H11O [7|1][1-6][2|3][1-5]	C33H44N5O9
655.3062	655.3212	-0.0150	-22.9	4.61	1	c5 -C9H11O -NH3 [7|1][1-6][2|3][1-5]	C33H44N5O9
655.3062	655.3212	-0.0150	-22.9	4.61	1	b5 -C9H11O [2|3][1-6][5|6][1-5]	C33H44N5O9
655.3062	655.3212	-0.0150	-22.9	4.61	1	c5 -C9H11O -NH3 [2|3][1-6][5|6][1-5]	C33H44N5O9
655.3062	655.3126	-0.0064	-9.8	4.61	1	x4 -CH3OH [7|1][1-5][1|2][2-5]	C37H42N4O7
655.3062	655.3126	-0.0064	-9.8	4.61	1	x4 -CH3OH [7|1][1-6][1|2][3-6]	C37H42N4O7
655.3062	655.3126	-0.0064	-9.8	4.61	1	x4 -CH3OH [2|3][1-6][3|4][3-6]	C37H42N4O7
655.3062	655.3126	-0.0064	-9.8	4.61	1	x4 -CH3OH [3|4][1-5][3|4][2-5]	C37H42N4O7
655.3062	655.3086	-0.0024	-3.7	4.61	1	b6 -H2O [5|6][1-6]	C32H42N6O9
655.3062	655.3086	-0.0024	-3.7	4.61	1	c6 -H2O -NH3 [5|6][1-6]	C32H42N6O9
661.3639	661.3748	-0.0109	-16.5	5.20	1	z5 -CH3OH -H2O [3|4][1-6][2|3][2-6]	C41H48N4O4
661.3639	661.3748	-0.0109	-16.5	5.20	1	z5 -CH3OH -H2O [6|7][1-6][3|4][2-6]	C41H48N4O4
661.3639	661.3596	0.0043	6.5	5.20	1	c4 -NH3 [7|1][1-6][3|4][1-4]	C37H48N4O7
661.3639	661.3596	0.0043	6.5	5.20	1	c4 -NH3 [2|3][1-6][5|6][1-4]	C37H48N4O7
661.3639	661.3596	0.0043	6.5	5.20	1	c4 -NH3 [3|4][1-5][4|5][1-4]	C37H48N4O7
661.3639	661.3596	0.0043	6.5	5.20	1	z5 -CH3OH [4|5][1-6][1|2][2-6]	C37H48N4O7
661.3639	661.3596	0.0043	6.5	5.20	1	b5 -CH3OH -NH3 [4|5][1-6][2|3][1-5]	C37H48N4O7
661.3639	661.3596	0.0043	6.5	5.20	1	z5 -CH3OH [6|7][1-6][4|5][2-6]	C37H48N4O7
661.3639	661.3596	0.0043	6.5	5.20	1	b5 -CH3OH -NH3 [6|7][1-6][5|6][1-5]	C37H48N4O7
661.3639	661.3596	0.0043	6.5	5.20	1	b4 [7|1][1-5][2|3][1-4]	C37H48N4O7
661.3639	661.3596	0.0043	6.5	5.20	1	b4 [7|1][1-6][3|4][1-4]	C37H48N4O7
661.3639	661.3596	0.0043	6.5	5.20	1	b4 [2|3][1-6][5|6][1-4]	C37H48N4O7
661.3639	661.3596	0.0043	6.5	5.20	1	b4 [3|4][1-5][4|5][1-4]	C37H48N4O7
661.3639	661.3596	0.0043	6.5	5.20	1	z5 -CH3OH [2|3][3-7]	C37H48N4O7
661.3639	661.3596	0.0043	6.5	5.20	1	b5 -CH3OH -NH3 [4|5][1-5]	C37H48N4O7
661.3639	661.3596	0.0043	6.5	5.20	1	c4 -NH3 [7|1][1-5][2|3][1-4]	C37H48N4O7
661.3639	661.3556	0.0083	12.6	5.20	1	c5 +CO -C9H10O -H2O [1|2][1-6][3|4][1-5]	C32H48N6O9
661.3639	661.3556	0.0083	12.6	5.20	1	c5 +CO -C9H10O -H2O [4|5][1-6][4|5][1-5]	C32H48N6O9
667.3762	667.3854	-0.0092	-13.7	1.66	1	b4 -H2O -H2O [1|2][1-4]	C40H50N4O5
667.3762	667.3854	-0.0092	-13.7	1.66	1	b4 -H2O -H2O [1|2][1-5][2|3][1-4]	C40H50N4O5
667.3762	667.3854	-0.0092	-13.7	1.66	1	b4 -H2O -H2O [3|4][1-6][5|6][1-4]	C40H50N4O5
667.3762	667.3814	-0.0051	-7.7	1.66	1	c5 +CO -C9H10O -H2O [7|1][1-5]	C35H50N6O7
667.3762	667.3814	-0.0051	-7.7	1.66	1	c5 +CO -C9H10O -H2O [7|1][1-6][3|4][1-5]	C35H50N6O7
667.3762	667.3814	-0.0051	-7.7	1.66	1	c5 +CO -C9H10O -H2O [3|4][1-6][4|5][1-5]	C35H50N6O7
667.3762	667.3701	0.0061	9.1	1.66	1	c4 +CO -NH3 [4|5][1-5][4|5][1-4]	C36H50N4O8
667.3762	667.3701	0.0061	9.1	1.66	1	b4 -H2O [1|2][1-5][3|4][1-4]	C36H50N4O8
667.3762	667.3701	0.0061	9.1	1.66	1	c4 -H2O -NH3 [1|2][1-5][3|4][1-4]	C36H50N4O8
667.3762	667.3701	0.0061	9.1	1.66	1	b4 -H2O [4|5][1-6][4|5][1-4]	C36H50N4O8
667.3762	667.3701	0.0061	9.1	1.66	1	c4 -H2O -NH3 [4|5][1-6][4|5][1-4]	C36H50N4O8
667.3762	667.3701	0.0061	9.1	1.66	1	b4 +CO [1|2][1-6][3|4][1-4]	C36H50N4O8
667.3762	667.3701	0.0061	9.1	1.66	1	b4 +CO [4|5][1-5][4|5][1-4]	C36H50N4O8
667.3762	667.3701	0.0061	9.1	1.66	1	c4 +CO -NH3 [1|2][1-6][3|4][1-4]	C36H50N4O8
667.3762	667.3661	0.0101	15.1	1.66	1	c5 +CO -C9H10O [4|5][1-6][3|4][1-5]	C31H50N6O10
667.3762	667.3661	0.0101	15.1	1.66	1	c5 +CO -C9H10O [7|1][1-6][4|5][1-5]	C31H50N6O10
673.3240	673.3317	-0.0078	-11.6	9.07	1	b5 +H2O -C9H11O [7|1][1-6][2|3][1-5]	C33H46N5O10
673.3240	673.3317	-0.0078	-11.6	9.07	1	b5 +H2O -C9H11O [2|3][1-6][5|6][1-5]	C33H46N5O10
673.3240	673.3232	0.0008	1.2	9.07	1	z5 -H2O -H2O [2|3][1-6][1|2][2-6]	C37H44N4O8
673.3240	673.3232	0.0008	1.2	9.07	1	z5 -H2O -H2O [4|5][1-6][4|5][2-6]	C37H44N4O8
673.3240	673.3192	0.0048	7.1	9.07	1	b6 [5|6][1-6]	C32H44N6O10
673.3240	673.3192	0.0048	7.1	9.07	1	c6 -NH3 [5|6][1-6]	C32H44N6O10

Meas. m/z	Calc. m/z	δ (Da)	δ (ppm)	Rel. Int. (%)	z	Annotation	Formula
674.3267	674.3396	-0.0128	-19.0	3.65	1	b5 +H2O -C9H10O [2|3][1-6][5|6][1-5]	C33H47N5O10
674.3267	674.3396	-0.0128	-19.0	3.65	1	b5 +H2O -C9H10O [7|1][1-6][2|3][1-5]	C33H47N5O10
675.3639	675.3752	-0.0113	-16.7	1.72	1	b5 -H2O -NH3 [4|5][1-5]	C38H50N4O7
675.3639	675.3752	-0.0113	-16.7	1.72	1	z5 -H2O [6|7][1-6][4|5][2-6]	C38H50N4O7
675.3639	675.3752	-0.0113	-16.7	1.72	1	z5 -H2O [2|3][3-7]	C38H50N4O7
675.3639	675.3752	-0.0113	-16.7	1.72	1	z5 -H2O [4|5][1-6][1|2][2-6]	C38H50N4O7
675.3639	675.3752	-0.0113	-16.7	1.72	1	b5 -H2O -NH3 [4|5][1-6][2|3][1-5]	C38H50N4O7
675.3639	675.3752	-0.0113	-16.7	1.72	1	b5 -H2O -NH3 [6|7][1-6][5|6][1-5]	C38H50N4O7
690.3443	690.3497	-0.0055	-7.9	1.67	1	b5 -H2O -H2O [2|3][1-6][2|3][1-5]	C37H47N5O8
690.3443	690.3497	-0.0055	-7.9	1.67	1	b5 -H2O -H2O [4|5][1-6][5|6][1-5]	C37H47N5O8
690.3443	690.3457	-0.0014	-2.1	1.67	1	c6 [5|6][1-6]	C32H47N7O10
690.3443	690.3385	0.0058	8.4	1.67	1	z5 -CH3OH -NH3 [7|1][1-6][3|4][2-6]	C38H47N3O9
690.3443	690.3385	0.0058	8.4	1.67	1	z5 -CH3OH -NH3 [4|5][1-6][2|3][2-6]	C38H47N3O9
693.3829	693.3858	-0.0028	-4.1	28.08	1	a5 -H2O -NH3 [7|1][1-6][4|5][1-5]	C38H52N4O8
693.3829	693.3858	-0.0028	-4.1	28.08	1	b5 -NH3 [4|5][1-6][2|3][1-5]	C38H52N4O8
693.3829	693.3858	-0.0028	-4.1	28.08	1	a5 -H2O -NH3 [4|5][1-6][3|4][1-5]	C38H52N4O8
693.3829	693.3858	-0.0028	-4.1	28.08	1	b5 -NH3 [6|7][1-6][5|6][1-5]	C38H52N4O8
693.3829	693.3858	-0.0028	-4.1	28.08	1	b5 -NH3 [4|5][1-5]	C38H52N4O8
693.3829	693.3858	-0.0028	-4.1	28.08	1	z5 [2|3][3-7]	C38H52N4O8
693.3829	693.3858	-0.0028	-4.1	28.08	1	z5 [6|7][1-6][4|5][2-6]	C38H52N4O8
693.3829	693.3858	-0.0028	-4.1	28.08	1	z5 [4|5][1-6][1|2][2-6]	C38H52N4O8
694.3885	694.3851	0.0035	5.0	12.01	1	z5 -NH3 [3|4][1-6][2|3][2-6]	C42H51N3O6
694.3885	694.3851	0.0035	5.0	12.01	1	z5 -NH3 [6|7][1-6][3|4][2-6]	C42H51N3O6
694.3885	694.3810	0.0075	10.8	12.01	1	c4 +CO -H2O -H2O [1|2][1-5][3|4][1-4]	C37H51N5O8
694.3885	694.3810	0.0075	10.8	12.01	1	c4 +CO -H2O -H2O [4|5][1-6][4|5][1-4]	C37H51N5O8
695.3849	695.3803	0.0046	6.7	2.21	1	b4 +CO -H2O -H2O [1|2][1-4]	C41H50N4O6
695.3849	695.3803	0.0046	6.7	2.21	1	b4 +CO -H2O -H2O [1|2][1-5][2|3][1-4]	C41H50N4O6
695.3849	695.3803	0.0046	6.7	2.21	1	b4 +CO -H2O -H2O [3|4][1-6][5|6][1-4]	C41H50N4O6
719.3621	719.3651	-0.0030	-4.1	3.82	1	b4 [2|3][1-4]	C39H50N4O9
719.3621	719.3651	-0.0030	-4.1	3.82	1	c4 -NH3 [2|3][1-4]	C39H50N4O9
719.3621	719.3651	-0.0030	-4.1	3.82	1	x5 -NH3 [2|3][3-7]	C39H50N4O9
719.3621	719.3651	-0.0030	-4.1	3.82	1	z5 -CH3OH [1|2][1-6][2|3][2-6]	C39H50N4O9
719.3621	719.3651	-0.0030	-4.1	3.82	1	b5 -CH3OH -NH3 [1|2][1-6][3|4][1-5]	C39H50N4O9
719.3621	719.3651	-0.0030	-4.1	3.82	1	x5 -NH3 [4|5][1-6][1|2][2-6]	C39H50N4O9
719.3621	719.3651	-0.0030	-4.1	3.82	1	z5 -CH3OH [4|5][1-6][3|4][2-6]	C39H50N4O9
719.3621	719.3651	-0.0030	-4.1	3.82	1	b5 -CH3OH -NH3 [4|5][1-6][4|5][1-5]	C39H50N4O9
719.3621	719.3651	-0.0030	-4.1	3.82	1	x5 -NH3 [6|7][1-6][4|5][2-6]	C39H50N4O9
729.3395	729.3494	-0.0099	-13.6	1.46	1	b4 +CO -H2O [2|3][1-4]	C40H48N4O9
729.3395	729.3494	-0.0099	-13.6	1.46	1	c4 +CO -H2O -NH3 [2|3][1-4]	C40H48N4O9
751.4013	751.4065	-0.0052	-6.9	4.46	1	z5 -H2O [1|2][1-6][1|2][2-6]	C44H54N4O7
751.4013	751.4065	-0.0052	-6.9	4.46	1	b5 -H2O -NH3 [1|2][1-6][2|3][1-5]	C44H54N4O7
751.4013	751.4065	-0.0052	-6.9	4.46	1	z5 -H2O [1|2][1-6][4|5][2-6]	C44H54N4O7
751.4013	751.4065	-0.0052	-6.9	4.46	1	b5 -H2O -NH3 [1|2][1-6][5|6][1-5]	C44H54N4O7
751.4013	751.4065	-0.0052	-6.9	4.46	1	z5 -H2O [3|4][1-6][4|5][2-6]	C44H54N4O7
751.4013	751.4065	-0.0052	-6.9	4.46	1	b5 -H2O -NH3 [3|4][1-6][5|6][1-5]	C44H54N4O7
751.4013	751.4065	-0.0052	-6.9	4.46	1	z5 -H2O [6|7][1-6][1|2][2-6]	C44H54N4O7
751.4013	751.4065	-0.0052	-6.9	4.46	1	b5 -H2O -NH3 [6|7][1-6][2|3][1-5]	C44H54N4O7
751.4013	751.4025	-0.0012	-1.6	4.46	1	b6 +CO -C9H10O [3|4][1-6]	C39H54N6O9
751.4013	751.4025	-0.0012	-1.6	4.46	1	b6 +CO -C9H10O [6|7][1-6]	C39H54N6O9
751.4013	751.4025	-0.0012	-1.6	4.46	1	c6 +CO -C9H10O -NH3 [3|4][1-6]	C39H54N6O9
751.4013	751.4025	-0.0012	-1.6	4.46	1	c6 +CO -C9H10O -NH3 [6|7][1-6]	C39H54N6O9
751.4013	751.4025	-0.0012	-1.6	4.46	1	c5 +CO -CH3OH -H2O [7|1][1-6][4|5][1-5]	C39H54N6O9
751.4013	751.4025	-0.0012	-1.6	4.46	1	c5 +CO -CH3OH -H2O [4|5][1-6][3|4][1-5]	C39H54N6O9
751.4013	751.4025	-0.0012	-1.6	4.46	1	b6 -C9H10O -H2O [7|1][1-6]	C39H54N6O9

Meas. m/z	Calc. m/z	δ (Da)	δ (ppm)	Rel. Int. (%)	z	Annotation	Formula
751.4013	751.3913	0.0100	13.4	4.46	1	b5 -NH3 [1|2][1-6][3|4][1-5]	C40H54N4O10
751.4013	751.3913	0.0100	13.4	4.46	1	b5 -NH3 [4|5][1-6][4|5][1-5]	C40H54N4O10
751.4013	751.3913	0.0100	13.4	4.46	1	z5 [1|2][1-6][2|3][2-6]	C40H54N4O10
751.4013	751.3913	0.0100	13.4	4.46	1	z5 [4|5][1-6][3|4][2-6]	C40H54N4O10
752.3997	752.4103	-0.0106	-14.1	3.84	1	a6 -C9H11O [1|2][1-6]	C39H55N6O9
752.3997	752.4018	-0.0020	-2.7	3.84	1	b5 +CO -CH3OH -H2O [3|4][1-6][4|5][1-5]	C43H53N5O7
752.3997	752.4018	-0.0020	-2.7	3.84	1	b5 +CO -CH3OH -H2O [7|1][1-6][3|4][1-5]	C43H53N5O7
752.3997	752.4018	-0.0020	-2.7	3.84	1	b5 +CO -CH3OH -H2O [7|1][1-5]	C43H53N5O7
752.3997	752.3905	0.0092	12.2	3.84	1	z5 -NH3 [1|2][1-6][1|2][2-6]	C44H53N3O8
752.3997	752.3905	0.0092	12.2	3.84	1	z5 -NH3 [1|2][1-6][4|5][2-6]	C44H53N3O8
752.3997	752.3905	0.0092	12.2	3.84	1	z5 -NH3 [3|4][1-6][4|5][2-6]	C44H53N3O8
752.3997	752.3905	0.0092	12.2	3.84	1	z5 -NH3 [6|7][1-6][1|2][2-6]	C44H53N3O8
752.3997	752.3865	0.0132	17.6	3.84	1	c5 +CO -CH3OH -NH3 [7|1][1-6][4|5][1-5]	C39H53N5O10
752.3997	752.3865	0.0132	17.6	3.84	1	b5 +CO -CH3OH [4|5][1-6][3|4][1-5]	C39H53N5O10
752.3997	752.3865	0.0132	17.6	3.84	1	c5 +CO -CH3OH -NH3 [4|5][1-6][3|4][1-5]	C39H53N5O10
752.3997	752.3865	0.0132	17.6	3.84	1	b6 -C9H10O -NH3 [7|1][1-6]	C39H53N5O10
752.3997	752.3865	0.0132	17.6	3.84	1	z6 -C9H10O [6|7][2-7]	C39H53N5O10
752.3997	752.3865	0.0132	17.6	3.84	1	b5 +CO -CH3OH [7|1][1-6][4|5][1-5]	C39H53N5O10
756.3532	756.3491	0.0042	5.5	2.12	1	z5 -NH3 [7|1][1-6][1|2][2-6]	C42H49N3O10
756.3532	756.3491	0.0042	5.5	2.12	1	z5 -NH3 [2|3][1-6][4|5][2-6]	C42H49N3O10
801.4548	801.4545	0.0003	0.3	16.68	1	c5 +CO -H2O [7|1][1-5]	C44H60N6O8
801.4548	801.4545	0.0003	0.3	16.68	1	c5 +CO -H2O [7|1][1-6][3|4][1-5]	C44H60N6O8
801.4548	801.4545	0.0003	0.3	16.68	1	c5 +CO -H2O [3|4][1-6][4|5][1-5]	C44H60N6O8
822.4350	822.4436	-0.0087	-10.5	2.11	1	z6 -H2O [2|3][2-7]	C47H59N5O8
822.4350	822.4436	-0.0087	-10.5	2.11	1	b6 -H2O -NH3 [3|4][1-6]	C47H59N5O8
822.4350	822.4436	-0.0087	-10.5	2.11	1	z6 -H2O [5|6][2-7]	C47H59N5O8
822.4350	822.4436	-0.0087	-10.5	2.11	1	b6 -H2O -NH3 [6|7][1-6]	C47H59N5O8
822.4350	822.4284	0.0066	8.0	2.11	1	z6 [3|4][2-7]	C43H59N5O11
822.4350	822.4284	0.0066	8.0	2.11	1	b6 -NH3 [4|5][1-6]	C43H59N5O11
834.4413	834.4396	0.0017	2.0	16.82	1	M -C9H10O -H2O [7|1][1-7]	C43H59N7O10
834.4413	834.4396	0.0017	2.0	16.82	1	c6 +CO -CH3OH -H2O [4|5][1-6]	C43H59N7O10
835.4278	835.4389	-0.0111	-13.3	37.88	1	b6 +CO -CH3OH -H2O [3|4][1-6]	C47H58N6O8
835.4278	835.4389	-0.0111	-13.3	37.88	1	b6 +CO -CH3OH -H2O [6|7][1-6]	C47H58N6O8
835.4278	835.4236	0.0042	5.0	37.88	1	M -C9H10O -NH3 [7|1][1-7]	C43H58N6O11
835.4278	835.4236	0.0042	5.0	37.88	1	c5 +CO [7|1][1-6][2|3][1-5]	C43H58N6O11
835.4278	835.4236	0.0042	5.0	37.88	1	c5 +CO [2|3][1-6][5|6][1-5]	C43H58N6O11
835.4278	835.4236	0.0042	5.0	37.88	1	b6 +CO -CH3OH [4|5][1-6]	C43H58N6O11
835.4278	835.4236	0.0042	5.0	37.88	1	c6 +CO -CH3OH -NH3 [4|5][1-6]	C43H58N6O11
836.4302	836.4229	0.0073	8.7	24.11	1	z6 -CH3OH -H2O [6|7][2-7]	C47H57N5O9
836.4302	836.4229	0.0073	8.7	24.11	1	b6 +CO -CH3OH -NH3 [3|4][1-6]	C47H57N5O9
836.4302	836.4229	0.0073	8.7	24.11	1	b6 +CO -CH3OH -NH3 [6|7][1-6]	C47H57N5O9
852.4511	852.4654	-0.0143	-16.8	100.00	1	c6 +CO -CH3OH -H2O [3|4][1-6]	C47H61N7O8
852.4511	852.4654	-0.0143	-16.8	100.00	1	c6 +CO -CH3OH -H2O [6|7][1-6]	C47H61N7O8
852.4511	852.4542	-0.0031	-3.6	100.00	1	a6 -H2O -NH3 [1|2][1-6]	C48H61N5O9
852.4511	852.4502	0.0009	1.1	100.00	1	M -C9H10O [7|1][1-7]	C43H61N7O11
852.4511	852.4502	0.0009	1.1	100.00	1	c6 +CO -CH3OH [4|5][1-6]	C43H61N7O11
853.4524	853.4495	0.0029	3.4	82.19	1	b6 -CH3OH -H2O [7|1][1-6]	C47H60N6O9
853.4524	853.4495	0.0029	3.4	82.19	1	b6 +CO -CH3OH [3|4][1-6]	C47H60N6O9
853.4524	853.4495	0.0029	3.4	82.19	1	c6 +CO -CH3OH -NH3 [3|4][1-6]	C47H60N6O9
853.4524	853.4495	0.0029	3.4	82.19	1	b6 +CO -CH3OH [6|7][1-6]	C47H60N6O9
853.4524	853.4495	0.0029	3.4	82.19	1	c6 +CO -CH3OH -NH3 [6|7][1-6]	C47H60N6O9
854.4550	854.4447	0.0103	12.0	17.97	1	c6 -H2O -H2O [2|3][1-6]	C46H59N7O9
885.4836	885.4757	0.0079	9.0	13.32	1	b6 -H2O [7|1][1-6]	C48H64N6O10
885.4836	885.4757	0.0079	9.0	13.32	1	c6 -H2O -NH3 [7|1][1-6]	C48H64N6O10

Meas. m/z	Calc. m/z	δ (Da)	δ (ppm)	Rel. Int. (%)	z	Annotation	Formula
885.4836	885.4757	0.0079	9.0	13.32	1	b6 +CO [3|4][1-6]	C48H64N6O10
885.4836	885.4757	0.0079	9.0	13.32	1	b6 +CO [6|7][1-6]	C48H64N6O10
885.4836	885.4757	0.0079	9.0	13.32	1	c6 +CO -NH3 [3|4][1-6]	C48H64N6O10
885.4836	885.4757	0.0079	9.0	13.32	1	c6 +CO -NH3 [6|7][1-6]	C48H64N6O10

Generated by mMass • Open Source Mass Spectrometry Tool • www.mmass.org

mMass Report: *Cryptophycin-1*

**Date**	Wed Oct 05 15:16:20 2011	**Scan Number**	0
**Operator**	Timo Niedermeyer	**Retention Time**	1222.8
**Contact**	timo.niedermeyer@cyano-biotech.com	**MS Level**	2
**Institution**	Cyano Biotech GmbH	**Precursor m/z**	655.28
**Instrument**	Shimadzu LCMS-IT-TOF ESI IT-TOF	**Polarity**	positive
		**Spectrum Points**	4945
		**Peak List**	36

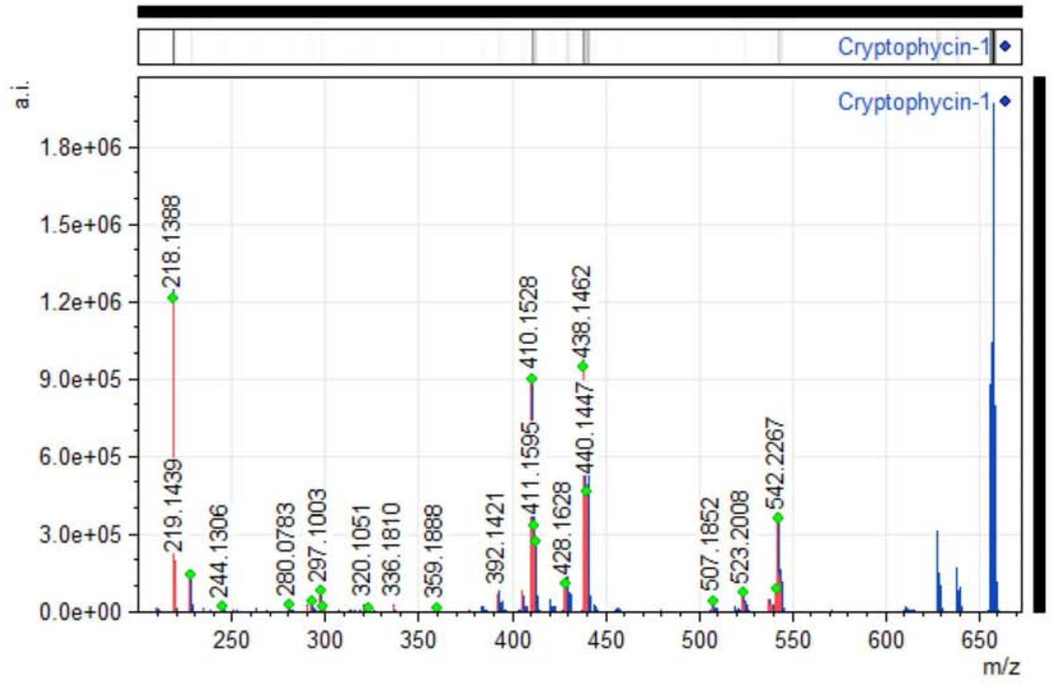

Sequence - *Cryptophycin-1*

Accession	Length	Mo. Mass	Av. Mass	Coverage	Matched Int.
	4 (Cyclic)	654.2708	655.1785	100.0 %	79.6 %

Epoxy-HMP-octenOOH | D-Cl-OMe-Tyr | Me-bAla | L-Leu-COOH

Meas. m/z	Calc. m/z	δ (Da)	δ (ppm)	Rel. Int. (%)	z	Annotation	Formula
218.1388	218.1387	0.0001	0.5	100.00	1	b2 +H2O [2|3][1-2]	C10H19NO4
227.1053	227.1067	-0.0014	-6.0	11.76	1	c1 -H2O -NH3 [4|1][1-1]	C15H14O2
227.1053	227.1067	-0.0014	-6.0	11.76	1	b1 -H2O [4|1][1-1]	C15H14O2
244.1306	244.1332	-0.0027	-10.9	1.53	1	c1 -H2O [4|1][1-1]	C15H17NO2
280.0783	280.0735	0.0048	17.1	2.10	1	b2 -NH3 [1|2][1-2]	C14ClH14NO3
280.0783	280.0735	0.0048	17.1	2.10	1	z2 [3|4][3-4]	C14ClH14NO3
292.0981	292.0861	0.0120	41.3	3.39	1	a2 -C8H7O -NH3 [4|1][1-2]	C16ClH16O3
297.1003	297.1000	0.0002	0.7	6.47	1	b2 [1|2][1-2]	C14ClH17N2O3
297.1003	297.1000	0.0002	0.7	6.47	1	c2 -NH3 [1|2][1-2]	C14ClH17N2O3
298.0943	298.0841	0.0102	34.3	1.47	1	b2 +H2O -NH3 [1|2][1-2]	C14ClH16NO4
323.0838	323.0793	0.0045	13.9	1.40	1	x2 [3|4][3-4]	C15ClH15N2O4
359.1888	359.1853	0.0035	9.7	1.37	1	c2 -NH3 [3|4][1-2]	C21H26O5

Meas. m/z	Calc. m/z	δ (Da)	δ (ppm)	Rel. Int. (%)	z	Annotation	Formula
359.1888	359.1853	0.0035	9.7	1.37	1	b2 [3|4][1-2]	C21H26O5
410.1528	410.1517	0.0011	2.7	74.30	1	a2 -H2O [4|1][1-2]	C24ClH24NO3
411.1595	411.1681	-0.0086	-20.9	27.65	1	b3 [1|2][1-3]	C20ClH27N2O5
411.1595	411.1681	-0.0086	-20.9	27.65	1	c3 -NH3 [1|2][1-3]	C20ClH27N2O5
411.1595	411.1556	0.0040	9.7	27.65	1	c3 -C10H11O [4|1][1-3]	C19ClH25N3O5
412.1531	412.1521	0.0010	2.4	22.19	1	b3 +H2O -NH3 [1|2][1-3]	C20ClH26NO6
412.1531	412.1396	0.0136	33.0	22.19	1	b3 +H2O -C10H11O [4|1][1-3]	C19ClH24N2O6
428.1628	428.1623	0.0005	1.1	8.72	1	a2 [4|1][1-2]	C24ClH26NO4
429.1757	429.1787	-0.0030	-6.9	16.29	1	b3 +H2O [1|2][1-3]	C20ClH29N2O6
438.1462	438.1467	-0.0004	-1.0	77.95	1	b2 -H2O [4|1][1-2]	C25ClH24NO4
438.1462	438.1467	-0.0004	-1.0	77.95	1	c2 -H2O -NH3 [4|1][1-2]	C25ClH24NO4
439.1482	439.1630	-0.0148	-33.7	38.30	1	b3 +CO [1|2][1-3]	C21ClH27N2O6
439.1482	439.1630	-0.0148	-33.7	38.30	1	c3 +CO -NH3 [1|2][1-3]	C21ClH27N2O6
439.1482	439.1505	-0.0022	-5.1	38.30	1	c3 +CO -C10H11O [4|1][1-3]	C20ClH25N3O6
507.1852	507.1933	-0.0081	-15.9	3.56	1	a3 -H2O -NH3 [3|4][1-3]	C30ClH31O5
523.2008	523.1994	0.0014	2.6	5.96	1	c3 -H2O -NH3 [4|1][1-3]	C29ClH31N2O5
523.2008	523.1994	0.0014	2.6	5.96	1	b3 -H2O [4|1][1-3]	C29ClH31N2O5
541.2084	541.2100	-0.0016	-2.9	7.12	1	b3 [4|1][1-3]	C29ClH33N2O6
541.2084	541.2100	-0.0016	-2.9	7.12	1	c3 -NH3 [4|1][1-3]	C29ClH33N2O6
542.2267	542.2304	-0.0037	-6.8	29.67	1	a3 [3|4][1-3]	C30ClH36NO6

Generated by mMass • Open Source Mass Spectrometry Tool • www.mmass.org

mMass Report: *Microcystin LR*

**Date**	Sun Aug 14 19:24:49 2011	**Scan Number**	1184
**Operator**	Timo Niedermeyer	**Retention Time**	722.5
**Contact**	timo.niedermeyer@cyano-biotech.com	**MS Level**	2
**Institution**	Cyano Biotech GmbH	**Precursor m/z**	995.53
**Instrument**	Shimadzu LCMS-IT-TOF ESI IT-TOF	**Polarity**	positive
		**Spectrum Points**	2284
		**Peak List**	67

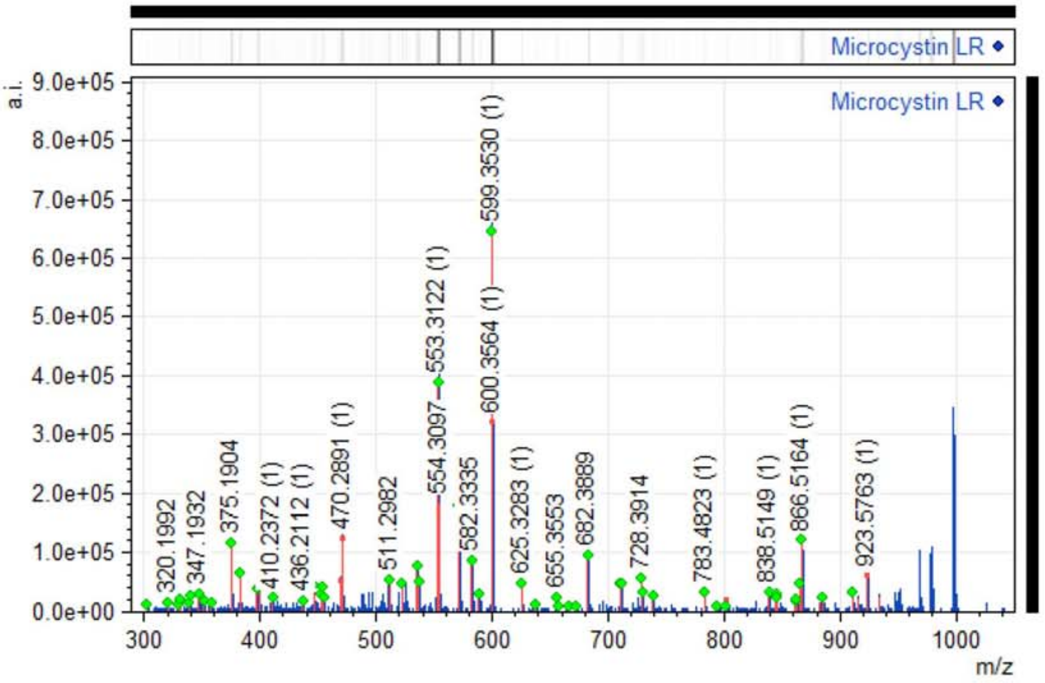

Sequence - *Microcystin LR*

Accession	Length	Mo. Mass	Av. Mass	Coverage	Matched Int.
	7 (Cyclic)	994.5488	995.1740	100.0 %	78.7 %

D-Ala | Leu | D-bMe-Asp | Arg | Adda | D-Glu | NMe-Dha

Meas. m/z	Calc. m/z	δ (Da)	δ (ppm)	Rel. Int. (%)	z	Annotation	Formula
302.1603	302.1751	-0.0148	-48.9	1.98	1	x2 -C9H10O -NH3 [4|5][1-5][1|2][4-5]	C18H23NO3
302.1603	302.1751	-0.0148	-48.9	1.98	1	x2 -C9H10O -NH3 [1|2][1-4][1|2][3-4]	C18H23NO3
302.1603	302.1625	-0.0022	-7.3	1.98	1	x2 -C9H11O -CH5N3 [5|6][6-7]	C17H21N2O3
302.1603	302.1625	-0.0022	-7.3	1.98	1	b2 +CO -C9H11O -CHN3H6 [3|4][1-2]	C17H21N2O3
320.1992	320.2094	-0.0102	-32.0	2.36	1	b2 +CO -C9H11O [1|2][1-4][3|4][1-2]	C18H27N2O3
320.1992	320.2094	-0.0102	-32.0	2.36	1	c2 +CO -C9H11O -NH3 [1|2][1-4][3|4][1-2]	C18H27N2O3
320.1992	320.2094	-0.0102	-32.0	2.36	1	b2 +CO -C9H11O [4|5][1-5][4|5][1-2]	C18H27N2O3
320.1992	320.2094	-0.0102	-32.0	2.36	1	c2 +CO -C9H11O -NH3 [4|5][1-5][4|5][1-2]	C18H27N2O3
320.1992	320.2009	-0.0017	-5.2	2.36	1	a2 -CH3OH -NH3 [4|5][1-3][2|3][1-2]	C22H25NO
320.1992	320.2009	-0.0017	-5.2	2.36	1	a2 -CH3OH -NH3 [6|7][1-6][5|6][1-2]	C22H25NO
320.1992	320.1969	0.0023	7.3	2.36	1	x2 -C9H10O -CN2H2 [5|6][6-7]	C17H25N3O3
320.1992	320.1969	0.0023	7.3	2.36	1	a4 -CH5N3 -NH3 [3|4][1-6][3|4][1-4]	C17H25N3O3

Meas. m/z	Calc. m/z	δ (Da)	δ (ppm)	Rel. Int. (%)	z	Annotation	Formula
320.1992	320.1969	0.0023	7.3	2.36	1	a4 -CH5N3 -NH3 [6|7][1-5][4|5][1-4]	C17H25N3O3
320.1992	320.1969	0.0023	7.3	2.36	1	c2 +CO -C9H10O -CHN3H6 [3|4][1-2]	C17H25N3O3
329.1758	329.1860	-0.0102	-30.9	1.98	1	z3 -C9H10O -CHN3H6 [7|1][1-5][1|2][3-5]	C19H24N2O3
329.1758	329.1860	-0.0102	-30.9	1.98	1	z3 -C9H10O -CHN3H6 [3|4][1-5][2|3][3-5]	C19H24N2O3
331.2028	331.2016	0.0012	3.7	3.25	1	z3 -C9H10O -CH5N3 [7|1][1-5][1|2][3-5]	C19H26N2O3
331.2028	331.2016	0.0012	3.7	3.25	1	z3 -C9H10O -CH5N3 [3|4][1-5][2|3][3-5]	C19H26N2O3
331.2028	331.1976	0.0052	15.8	3.25	1	c3 [7|1][1-3]	C14H26N4O5
331.2028	331.1976	0.0052	15.8	3.25	1	c3 [7|1][1-6][5|6][1-3]	C14H26N4O5
331.2028	331.1976	0.0052	15.8	3.25	1	c3 [5|6][1-4][2|3][1-3]	C14H26N4O5
338.1415	338.1347	0.0068	20.2	2.47	1	c3 +CO -CH5N3 -NH3 [5|6][1-6][5|6][1-3]	C15H19N3O6
338.1415	338.1347	0.0068	20.2	2.47	1	b3 +CO -CH5N3 [6|7][1-5][3|4][1-3]	C15H19N3O6
338.1415	338.1347	0.0068	20.2	2.47	1	b3 +CO -CN2H2 -NH3 [6|7][1-5][3|4][1-3]	C15H19N3O6
338.1415	338.1347	0.0068	20.2	2.47	1	c3 +CO -CH5N3 -NH3 [6|7][1-5][3|4][1-3]	C15H19N3O6
338.1415	338.1347	0.0068	20.2	2.47	1	z3 -CN2H2 -H2O [2|3][1-4][2|3][2-4]	C15H19N3O6
338.1415	338.1347	0.0068	20.2	2.47	1	b3 -CH5N3 -H2O [2|3][1-4][3|4][1-3]	C15H19N3O6
338.1415	338.1347	0.0068	20.2	2.47	1	z3 -CN2H2 -H2O [5|6][1-6][1|2][4-6]	C15H19N3O6
338.1415	338.1347	0.0068	20.2	2.47	1	b3 -CH5N3 -H2O [5|6][1-6][4|5][1-3]	C15H19N3O6
338.1415	338.1347	0.0068	20.2	2.47	1	b3 +CO -CH5N3 [2|3][1-5][4|5][1-3]	C15H19N3O6
338.1415	338.1347	0.0068	20.2	2.47	1	b3 +CO -CN2H2 -NH3 [2|3][1-5][4|5][1-3]	C15H19N3O6
338.1415	338.1347	0.0068	20.2	2.47	1	c3 +CO -CH5N3 -NH3 [2|3][1-5][4|5][1-3]	C15H19N3O6
338.1415	338.1347	0.0068	20.2	2.47	1	b3 +CO -CH5N3 [3|4][1-4][2|3][1-3]	C15H19N3O6
338.1415	338.1347	0.0068	20.2	2.47	1	b3 +CO -CN2H2 -NH3 [3|4][1-4][2|3][1-3]	C15H19N3O6
338.1415	338.1347	0.0068	20.2	2.47	1	c3 +CO -CH5N3 -NH3 [3|4][1-4][2|3][1-3]	C15H19N3O6
338.1415	338.1347	0.0068	20.2	2.47	1	b3 +CO -CH5N3 [5|6][1-6][5|6][1-3]	C15H19N3O6
338.1415	338.1347	0.0068	20.2	2.47	1	b3 +CO -CN2H2 -NH3 [5|6][1-6][5|6][1-3]	C15H19N3O6
338.1415	338.1347	0.0068	20.2	2.47	1	c3 +CO -CH5N3 -NH3 [5|6][1-6][5|6][1-3]	C15H19N3O6
338.1415	338.1347	0.0068	20.2	2.47	1	b3 +CO -CH5N3 [6|7][1-5][3|4][1-3]	C15H19N3O6
338.1415	338.1347	0.0068	20.2	2.47	1	b3 +CO -CN2H2 -NH3 [6|7][1-5][3|4][1-3]	C15H19N3O6
338.1415	338.1347	0.0068	20.2	2.47	1	c3 +CO -CH5N3 -NH3 [6|7][1-5][3|4][1-3]	C15H19N3O6
338.1415	338.1347	0.0068	20.2	2.47	1	z3 -CN2H2 -H2O [2|3][1-4][2|3][2-4]	C15H19N3O6
338.1415	338.1347	0.0068	20.2	2.47	1	b3 -CH5N3 -H2O [2|3][1-4][3|4][1-3]	C15H19N3O6
338.1415	338.1347	0.0068	20.2	2.47	1	z3 -CN2H2 -H2O [5|6][1-6][1|2][4-6]	C15H19N3O6
338.1415	338.1347	0.0068	20.2	2.47	1	b3 -CH5N3 -H2O [5|6][1-6][4|5][1-3]	C15H19N3O6
338.1415	338.1347	0.0068	20.2	2.47	1	b3 +CO -CH5N3 [2|3][1-5][4|5][1-3]	C15H19N3O6
338.1415	338.1347	0.0068	20.2	2.47	1	b3 +CO -CN2H2 -NH3 [2|3][1-5][4|5][1-3]	C15H19N3O6
338.1415	338.1347	0.0068	20.2	2.47	1	c3 +CO -CH5N3 -NH3 [2|3][1-5][4|5][1-3]	C15H19N3O6
338.1415	338.1347	0.0068	20.2	2.47	1	b3 +CO -CH5N3 [3|4][1-4][2|3][1-3]	C15H19N3O6
338.1415	338.1347	0.0068	20.2	2.47	1	b3 +CO -CN2H2 -NH3 [3|4][1-4][2|3][1-3]	C15H19N3O6
338.1415	338.1347	0.0068	20.2	2.47	1	c3 +CO -CH5N3 -NH3 [3|4][1-4][2|3][1-3]	C15H19N3O6
338.1415	338.1347	0.0068	20.2	2.47	1	b3 +CO -CH5N3 [5|6][1-6][5|6][1-3]	C15H19N3O6
338.1415	338.1347	0.0068	20.2	2.47	1	b3 +CO -CN2H2 -NH3 [5|6][1-6][5|6][1-3]	C15H19N3O6
339.2042	339.2027	0.0015	4.5	4.21	1	b3 -CN2H2 -H2O [1|2][1-3]	C16H26N4O4
339.2042	339.2027	0.0015	4.5	4.21	1	c3 -CH5N3 -H2O [1|2][1-3]	C16H26N4O4
347.1932	347.2078	-0.0145	-41.9	4.77	1	z4 -CN2H2 -H2O [3|4][1-6][1|2][3-6]	C18H26N4O3
347.1932	347.2078	-0.0145	-41.9	4.77	1	b4 -CH5N3 -H2O [3|4][1-6][3|4][1-4]	C18H26N4O3
347.1932	347.2078	-0.0145	-41.9	4.77	1	z4 -CN2H2 -H2O [6|7][1-5][3|4][2-5]	C18H26N4O3
347.1932	347.2078	-0.0145	-41.9	4.77	1	b4 -CH5N3 -H2O [6|7][1-5][4|5][1-4]	C18H26N4O3
347.1932	347.2078	-0.0145	-41.9	4.77	1	b2 +CO -C9H10O -NH3 [3|4][1-2]	C18H26N4O3
347.1932	347.1965	-0.0033	-9.5	4.77	1	a3 -C9H10O -NH3 [4|5][1-3]	C19H26N2O4
351.1949	351.2027	-0.0078	-22.3	2.98	1	a4 -H2O [5|6][1-4]	C17H26N4O4
351.1949	351.2027	-0.0078	-22.3	2.98	1	a4 -H2O [6|7][1-4]	C17H26N4O4
351.1949	351.1955	-0.0006	-1.8	2.98	1	z2 -NH3 [7|1][1-5][1|2][4-5]	C23H26O3
351.1949	351.1955	-0.0006	-1.8	2.98	1	z2 -NH3 [4|5][1-4][1|2][3-4]	C23H26O3
357.2016	357.2132	-0.0116	-32.5	2.47	1	c3 -CH5N3 [1|2][1-3]	C16H28N4O5

Meas. m/z	Calc. m/z	δ (Da)	δ (ppm)	Rel. Int. (%)	z	Annotation	Formula
357.2016	357.2132	-0.0116	-32.5	2.47	1	c3 -CN2H2 -NH3 [1|2][1-3]	C16H28N4O5
357.2016	357.2132	-0.0116	-32.5	2.47	1	b3 -CN2H2 [1|2][1-3]	C16H28N4O5
357.2016	357.2047	-0.0031	-8.5	2.47	1	b3 -C9H11O -CHN3H6 [3|4][1-4][3|4][1-3]	C20H26N3O3
357.2016	357.2047	-0.0031	-8.5	2.47	1	b3 -C9H11O -CHN3H6 [6|7][1-6][4|5][1-3]	C20H26N3O3
357.2016	357.1881	0.0135	37.9	2.47	1	c3 -NH3 [7|1][1-4][2|3][1-3]	C14H24N6O5
357.2016	357.1881	0.0135	37.9	2.47	1	c3 -NH3 [2|3][1-6][5|6][1-3]	C14H24N6O5
357.2016	357.1881	0.0135	37.9	2.47	1	b3 [7|1][1-4][2|3][1-3]	C14H24N6O5
357.2016	357.1881	0.0135	37.9	2.47	1	b3 [2|3][1-6][5|6][1-3]	C14H24N6O5
375.1904	375.2027	-0.0123	-32.9	18.15	1	a4 -CHN3H6 -H2O [1|2][1-6][5|6][1-4]	C19H26N4O4
375.1904	375.2027	-0.0123	-32.9	18.15	1	a4 -CHN3H6 -H2O [6|7][1-5][2|3][1-4]	C19H26N4O4
375.1904	375.2027	-0.0123	-32.9	18.15	1	b4 +CO -CH5N3 -H2O [3|4][1-6][3|4][1-4]	C19H26N4O4
375.1904	375.2027	-0.0123	-32.9	18.15	1	b4 +CO -CH5N3 -H2O [6|7][1-5][4|5][1-4]	C19H26N4O4
375.1904	375.1987	-0.0083	-22.1	18.15	1	b3 +H2O [7|1][1-4][2|3][1-3]	C14H26N6O6
375.1904	375.1987	-0.0083	-22.1	18.15	1	b3 +H2O [2|3][1-6][5|6][1-3]	C14H26N6O6
375.1904	375.1955	-0.0051	-13.6	18.15	1	z2 -CHN3H6 -NH3 [5|6][6-7]	C25H26O3
375.1904	375.1914	-0.0011	-2.9	18.15	1	z3 -C9H10O [7|1][5-7]	C20H26N2O5
375.1904	375.1914	-0.0011	-2.9	18.15	1	b3 -C9H10O -NH3 [4|5][1-3]	C20H26N2O5
382.2053	382.2085	-0.0032	-8.3	9.96	1	z3 [4|5][5-7]	C17H27N5O5
382.2053	382.2085	-0.0032	-8.3	9.96	1	b3 -NH3 [1|2][1-3]	C17H27N5O5
382.2053	382.2085	-0.0032	-8.3	9.96	1	c3 -NH3 -NH3 [1|2][1-3]	C17H27N5O5
397.2341	397.2486	-0.0145	-36.5	6.16	1	c2 -NH3 [6|7][1-6][5|6][1-2]	C24H32N2O3
397.2341	397.2486	-0.0145	-36.5	6.16	1	a2 -H2O [4|5][1-6][5|6][1-2]	C24H32N2O3
397.2341	397.2486	-0.0145	-36.5	6.16	1	b2 +H2O -CH3OH -CH5N3 [3|4][1-2]	C24H32N2O3
397.2341	397.2486	-0.0145	-36.5	6.16	1	b2 [4|5][1-3][2|3][1-2]	C24H32N2O3
397.2341	397.2486	-0.0145	-36.5	6.16	1	b2 [6|7][1-6][5|6][1-2]	C24H32N2O3
397.2341	397.2486	-0.0145	-36.5	6.16	1	a2 -H2O [4|5][1-2]	C24H32N2O3
397.2341	397.2486	-0.0145	-36.5	6.16	1	a2 -H2O [2|3][1-3][2|3][1-2]	C24H32N2O3
397.2341	397.2486	-0.0145	-36.5	6.16	1	c2 -NH3 [4|5][1-3][2|3][1-2]	C24H32N2O3
397.2341	397.2445	-0.0105	-26.4	6.16	1	c3 -C9H10O [4|5][1-4][3|4][1-3]	C19H32N4O5
397.2341	397.2445	-0.0105	-26.4	6.16	1	c3 -C9H10O [7|1][1-6][4|5][1-3]	C19H32N4O5
397.2341	397.2207	0.0133	33.5	6.16	1	b3 +H2O -C9H11O [7|1][1-6][4|5][1-3]	C19H30N3O6
397.2341	397.2207	0.0133	33.5	6.16	1	b3 +H2O -C9H11O [4|5][1-4][3|4][1-3]	C19H30N3O6
397.2341	397.2194	0.0147	36.9	6.16	1	c4 -CN2H2 -H2O [5|6][1-6][5|6][1-4]	C17H28N6O5
397.2341	397.2194	0.0147	36.9	6.16	1	c4 -CN2H2 -H2O [6|7][1-5][3|4][1-4]	C17H28N6O5
397.2341	397.2194	0.0147	36.9	6.16	1	c4 -CN2H2 -H2O [2|3][1-6][4|5][1-4]	C17H28N6O5
397.2341	397.2194	0.0147	36.9	6.16	1	c4 -CN2H2 -H2O [3|4][1-5][2|3][1-4]	C17H28N6O5
399.2356	399.2350	0.0006	1.4	6.62	1	c3 -NH3 [1|2][1-3]	C17H30N6O5
399.2356	399.2350	0.0006	1.4	6.62	1	b3 [1|2][1-3]	C17H30N6O5
410.2372	410.2438	-0.0066	-16.1	3.50	1	c2 -CH3OH -H2O [4|5][1-2]	C24H31N3O3
410.2372	410.2438	-0.0066	-16.1	3.50	1	c2 -CH3OH -H2O [2|3][1-3][2|3][1-2]	C24H31N3O3
410.2372	410.2438	-0.0066	-16.1	3.50	1	c2 -CH3OH -H2O [4|5][1-6][5|6][1-2]	C24H31N3O3
410.2372	410.2438	-0.0066	-16.1	3.50	1	c2 +CO -CH3OH [4|5][1-3][2|3][1-2]	C24H31N3O3
410.2372	410.2438	-0.0066	-16.1	3.50	1	c2 +CO -CH3OH [6|7][1-6][5|6][1-2]	C24H31N3O3
410.2372	410.2398	-0.0026	-6.3	3.50	1	c4 -CH5N3 -H2O [7|1][1-4]	C19H31N5O5
410.2372	410.2398	-0.0026	-6.3	3.50	1	b4 -CN2H2 -H2O [7|1][1-4]	C19H31N5O5
410.2372	410.2398	-0.0026	-6.3	3.50	1	b4 +CO -CN2H2 [3|4][1-6][3|4][1-4]	C19H31N5O5
410.2372	410.2398	-0.0026	-6.3	3.50	1	c4 +CO -CH5N3 [3|4][1-6][3|4][1-4]	C19H31N5O5
410.2372	410.2398	-0.0026	-6.3	3.50	1	c4 +CO -CN2H2 -NH3 [3|4][1-6][3|4][1-4]	C19H31N5O5
410.2372	410.2398	-0.0026	-6.3	3.50	1	c4 +CO -CH5N3 [6|7][1-5][4|5][1-4]	C19H31N5O5
410.2372	410.2398	-0.0026	-6.3	3.50	1	b4 +CO -CN2H2 [6|7][1-5][4|5][1-4]	C19H31N5O5
410.2372	410.2398	-0.0026	-6.3	3.50	1	c4 +CO -CN2H2 -NH3 [6|7][1-5][4|5][1-4]	C19H31N5O5
410.2372	410.2326	0.0046	11.2	3.50	1	b2 +H2O -CHN3H6 -NH3 [3|4][1-2]	C25H31NO4
410.2372	410.2286	0.0086	21.0	3.50	1	a3 -C9H10O [2|3][1-4][2|3][1-3]	C20H31N3O6
410.2372	410.2286	0.0086	21.0	3.50	1	b3 +H2O -C9H10O [4|5][1-3]	C20H31N3O6

Meas. m/z	Calc. m/z	δ (Da)	δ (ppm)	Rel. Int. (%)	z	Annotation	Formula
410.2372	410.2286	0.0086	21.0	3.50	1	a3 -C9H10O [4|5][1-6][5|6][1-3]	C20H31N3O6
436.2112	436.2191	-0.0078	-18.0	2.84	1	b4 -NH3 -NH3 [7|1][1-4]	C20H29N5O6
436.2112	436.2191	-0.0078	-18.0	2.84	1	x4 -CN2H2 -H2O [4|5][4-7]	C20H29N5O6
436.2112	436.2191	-0.0078	-18.0	2.84	1	z4 -NH3 [4|5][4-7]	C20H29N5O6
436.2112	436.2191	-0.0078	-18.0	2.84	1	c4 -H2O -H2O [1|2][1-6][4|5][1-4]	C20H29N5O6
436.2112	436.2191	-0.0078	-18.0	2.84	1	c4 -H2O -H2O [5|6][1-5][3|4][1-4]	C20H29N5O6
436.2112	436.2191	-0.0078	-18.0	2.84	1	c4 +CO -CHN3H6 -H2O [7|1][1-4]	C20H29N5O6
436.2112	436.2118	-0.0006	-1.4	2.84	1	b2 +CO -H2O -NH3 [4|5][1-2]	C26H29NO5
436.2112	436.2118	-0.0006	-1.4	2.84	1	b2 +CO -H2O -NH3 [2|3][1-3][2|3][1-2]	C26H29NO5
436.2112	436.2118	-0.0006	-1.4	2.84	1	b2 +CO -H2O -NH3 [4|5][1-6][5|6][1-2]	C26H29NO5
446.2253	446.2398	-0.0145	-32.4	7.06	1	a5 -CHN3H6 -H2O [6|7][1-5]	C22H31N5O5
446.2253	446.2398	-0.0145	-32.4	7.06	1	a5 -CHN3H6 -H2O [3|4][1-6][2|3][1-5]	C22H31N5O5
446.2253	446.2398	-0.0145	-32.4	7.06	1	a5 -CHN3H6 -H2O [5|6][1-6][5|6][1-5]	C22H31N5O5
446.2253	446.2326	-0.0072	-16.2	7.06	1	z3 -CHN3H6 -NH3 [7|1][1-5][1|2][3-5]	C28H31NO4
446.2253	446.2326	-0.0072	-16.2	7.06	1	z3 -CHN3H6 -NH3 [3|4][1-5][2|3][3-5]	C28H31NO4
446.2253	446.2286	-0.0032	-7.2	7.06	1	z4 -C9H10O [1|2][4-7]	C23H31N3O6
446.2253	446.2286	-0.0032	-7.2	7.06	1	b4 -C9H10O -NH3 [4|5][1-4]	C23H31N3O6
452.2747	452.2616	0.0131	28.9	4.72	1	b4 -H2O [7|1][1-4]	C20H33N7O5
452.2747	452.2616	0.0131	28.9	4.72	1	c4 -H2O -NH3 [7|1][1-4]	C20H33N7O5
452.2747	452.2616	0.0131	28.9	4.72	1	b4 +CO [3|4][1-6][3|4][1-4]	C20H33N7O5
452.2747	452.2616	0.0131	28.9	4.72	1	b4 +CO [6|7][1-5][4|5][1-4]	C20H33N7O5
452.2747	452.2616	0.0131	28.9	4.72	1	c4 +CO -NH3 [3|4][1-6][3|4][1-4]	C20H33N7O5
452.2747	452.2616	0.0131	28.9	4.72	1	c4 +CO -NH3 [6|7][1-5][4|5][1-4]	C20H33N7O5
453.2667	453.2748	-0.0081	-17.9	6.24	1	x2 [4|5][1-5][1|2][4-5]	C27H36N2O4
453.2667	453.2748	-0.0081	-17.9	6.24	1	x2 [1|2][1-4][1|2][3-4]	C27H36N2O4
454.2725	454.2772	-0.0047	-10.4	3.50	1	a4 [1|2][1-6][5|6][1-4]	C20H35N7O5
454.2725	454.2772	-0.0047	-10.4	3.50	1	a4 [6|7][1-5][2|3][1-4]	C20H35N7O5
454.2725	454.2700	0.0025	5.5	3.50	1	x2 -CN2H2 [5|6][6-7]	C26H35N3O4
454.2725	454.2700	0.0025	5.5	3.50	1	a3 -CH3OH [7|1][1-6][4|5][1-3]	C26H35N3O4
454.2725	454.2700	0.0025	5.5	3.50	1	a3 -CH3OH [4|5][1-4][3|4][1-3]	C26H35N3O4
454.2725	454.2700	0.0025	5.5	3.50	1	b2 +H2O -NH3 -NH3 [3|4][1-2]	C26H35N3O4
454.2725	454.2700	0.0025	5.5	3.50	1	c2 +CO -CHN3H6 [3|4][1-2]	C26H35N3O4
454.2725	454.2700	0.0025	5.5	3.50	1	b3 +H2O -CH3OH [4|5][1-4][2|3][1-3]	C26H35N3O4
454.2725	454.2700	0.0025	5.5	3.50	1	b3 +H2O -CH3OH [6|7][1-6][5|6][1-3]	C26H35N3O4
454.2725	454.2687	0.0038	8.5	3.50	1	z4 -C9H11O -H2O [3|4][1-5][2|3][2-5]	C24H33N6O3
454.2725	454.2687	0.0038	8.5	3.50	1	z4 -C9H11O -H2O [6|7][1-6][2|3][3-6]	C24H33N6O3
511.2982	511.3126	-0.0144	-28.2	8.46	1	b4 +H2O -C9H10O [7|1][1-5][4|5][1-4]	C25H42N4O7
511.2982	511.3126	-0.0144	-28.2	8.46	1	b4 +H2O -C9H10O [7|1][1-6][4|5][1-4]	C25H42N4O7
511.2982	511.3126	-0.0144	-28.2	8.46	1	b4 +H2O -C9H10O [4|5][1-5][3|4][1-4]	C25H42N4O7
511.2982	511.3126	-0.0144	-28.2	8.46	1	b4 +H2O -C9H10O [4|5][1-6][3|4][1-4]	C25H42N4O7
511.2982	511.3001	-0.0018	-3.6	8.46	1	b4 +H2O -C9H11O -CN2H2 [3|4][1-5][4|5][1-4]	C24H40N5O7
511.2982	511.3001	-0.0018	-3.6	8.46	1	b4 +H2O -C9H11O -CN2H2 [7|1][1-6][3|4][1-4]	C24H40N5O7
511.2982	511.3001	-0.0018	-3.6	8.46	1	b4 +H2O -C9H11O -CN2H2 [2|3][1-6][5|6][1-4]	C24H40N5O7
511.2982	511.3001	-0.0018	-3.6	8.46	1	b4 +H2O -C9H11O -CN2H2 [7|1][1-5][2|3][1-4]	C24H40N5O7
511.2982	511.2915	0.0067	13.1	8.46	1	c3 -CH3OH [4|5][1-3]	C28H38N4O5
511.2982	511.2875	0.0107	21.0	8.46	1	b5 -CN2H2 [6|7][1-5]	C23H38N6O7
511.2982	511.2875	0.0107	21.0	8.46	1	c5 -CH5N3 [6|7][1-5]	C23H38N6O7
511.2982	511.2875	0.0107	21.0	8.46	1	c5 -CN2H2 -NH3 [6|7][1-5]	C23H38N6O7
511.2982	511.2875	0.0107	21.0	8.46	1	a5 -CN2H2 -H2O [7|1][1-6][5|6][1-5]	C23H38N6O7
511.2982	511.2875	0.0107	21.0	8.46	1	b5 -CN2H2 [3|4][1-6][2|3][1-5]	C23H38N6O7
511.2982	511.2875	0.0107	21.0	8.46	1	c5 -CH5N3 [3|4][1-6][2|3][1-5]	C23H38N6O7
511.2982	511.2875	0.0107	21.0	8.46	1	c5 -CN2H2 -NH3 [3|4][1-6][2|3][1-5]	C23H38N6O7
511.2982	511.2875	0.0107	21.0	8.46	1	a5 -CN2H2 -H2O [5|6][1-6][2|3][1-5]	C23H38N6O7
511.2982	511.2875	0.0107	21.0	8.46	1	b5 -CN2H2 [5|6][1-6][5|6][1-5]	C23H38N6O7

Meas. m/z	Calc. m/z	δ (Da)	δ (ppm)	Rel. Int. (%)	z	Annotation	Formula
511.2982	511.2875	0.0107	21.0	8.46	1	c5 -CH5N3 [5|6][1-6][5|6][1-5]	C23H38N6O7
511.2982	511.2875	0.0107	21.0	8.46	1	c5 -CN2H2 -NH3 [5|6][1-6][5|6][1-5]	C23H38N6O7
522.2687	522.2599	0.0089	17.0	7.46	1	x3 -H2O [7|1][1-6][1|2][4-6]	C29H35N3O6
522.2687	522.2599	0.0089	17.0	7.46	1	b3 -CH3OH -H2O [2|3][1-4][2|3][1-3]	C29H35N3O6
522.2687	522.2599	0.0089	17.0	7.46	1	x3 -H2O [4|5][1-4][2|3][2-4]	C29H35N3O6
522.2687	522.2599	0.0089	17.0	7.46	1	b3 -CH3OH -H2O [4|5][1-6][5|6][1-3]	C29H35N3O6
522.2687	522.2599	0.0089	17.0	7.46	1	c3 +CO -CH3OH -NH3 [4|5][1-3]	C29H35N3O6
522.2687	522.2599	0.0089	17.0	7.46	1	b3 +CO -CH3OH [4|5][1-3]	C29H35N3O6
522.2687	522.2558	0.0129	24.7	7.46	1	b5 -CH5N3 -H2O [7|1][1-6][5|6][1-5]	C24H35N5O8
522.2687	522.2558	0.0129	24.7	7.46	1	z5 -CN2H2 -H2O [7|1][1-6][4|5][2-6]	C24H35N5O8
522.2687	522.2558	0.0129	24.7	7.46	1	a5 -CHN3H6 [1|2][1-6][4|5][1-5]	C24H35N5O8
522.2687	522.2558	0.0129	24.7	7.46	1	z5 -CN2H2 -H2O [5|6][1-6][1|2][2-6]	C24H35N5O8
522.2687	522.2558	0.0129	24.7	7.46	1	a5 -CHN3H6 [5|6][1-6][3|4][1-5]	C24H35N5O8
522.2687	522.2558	0.0129	24.7	7.46	1	b5 -CH5N3 -H2O [5|6][1-6][2|3][1-5]	C24H35N5O8
522.2687	522.2558	0.0129	24.7	7.46	1	b5 +CO -CH5N3 [6|7][1-5]	C24H35N5O8
522.2687	522.2558	0.0129	24.7	7.46	1	b5 +CO -CH5N3 [5|6][1-6][5|6][1-5]	C24H35N5O8
522.2687	522.2558	0.0129	24.7	7.46	1	c5 +CO -CH5N3 -NH3 [6|7][1-5]	C24H35N5O8
522.2687	522.2558	0.0129	24.7	7.46	1	b5 +CO -CN2H2 -NH3 [6|7][1-5]	C24H35N5O8
522.2687	522.2558	0.0129	24.7	7.46	1	b5 +CO -CH5N3 [3|4][1-6][2|3][1-5]	C24H35N5O8
522.2687	522.2558	0.0129	24.7	7.46	1	b5 +CO -CN2H2 -NH3 [3|4][1-6][2|3][1-5]	C24H35N5O8
522.2687	522.2558	0.0129	24.7	7.46	1	c5 +CO -CH5N3 -NH3 [3|4][1-6][2|3][1-5]	C24H35N5O8
522.2687	522.2558	0.0129	24.7	7.46	1	b5 +CO -CN2H2 -NH3 [5|6][1-6][5|6][1-5]	C24H35N5O8
522.2687	522.2558	0.0129	24.7	7.46	1	c5 +CO -CH5N3 -NH3 [5|6][1-6][5|6][1-5]	C24H35N5O8
535.2988	535.3126	-0.0138	-25.8	11.88	1	b4 +H2O -C9H10O -CHN3H6 [3|4][1-6][5|6][1-4]	C27H42N4O7
535.2988	535.3126	-0.0138	-25.8	11.88	1	b4 +H2O -C9H10O -CHN3H6 [1|2][1-4]	C27H42N4O7
535.2988	535.3126	-0.0138	-25.8	11.88	1	b4 +H2O -C9H10O -CHN3H6 [1|2][1-5][2|3][1-4]	C27H42N4O7
535.2988	535.3113	-0.0125	-23.3	11.88	1	b4 -C9H11O [7|1][1-5][2|3][1-4]	C25H40N7O6
535.2988	535.3113	-0.0125	-23.3	11.88	1	c4 -C9H11O -NH3 [7|1][1-5][2|3][1-4]	C25H40N7O6
535.2988	535.3113	-0.0125	-23.3	11.88	1	b4 -C9H11O [7|1][1-6][3|4][1-4]	C25H40N7O6
535.2988	535.3113	-0.0125	-23.3	11.88	1	c4 -C9H11O -NH3 [7|1][1-6][3|4][1-4]	C25H40N7O6
535.2988	535.3113	-0.0125	-23.3	11.88	1	b4 -C9H11O [2|3][1-6][5|6][1-4]	C25H40N7O6
535.2988	535.3113	-0.0125	-23.3	11.88	1	c4 -C9H11O -NH3 [2|3][1-6][5|6][1-4]	C25H40N7O6
535.2988	535.3113	-0.0125	-23.3	11.88	1	b4 -C9H11O [3|4][1-5][4|5][1-4]	C25H40N7O6
535.2988	535.3113	-0.0125	-23.3	11.88	1	c4 -C9H11O -NH3 [3|4][1-5][4|5][1-4]	C25H40N7O6
535.2988	535.3027	-0.0039	-7.3	11.88	1	x3 -CH3OH [7|1][1-5][1|2][3-5]	C29H38N6O4
535.2988	535.3027	-0.0039	-7.3	11.88	1	x3 -CH3OH [3|4][1-5][2|3][3-5]	C29H38N6O4
535.2988	535.2987	0.0001	0.2	11.88	1	b5 -H2O [6|7][1-5]	C24H38N8O6
535.2988	535.2987	0.0001	0.2	11.88	1	c5 -H2O -NH3 [6|7][1-5]	C24H38N8O6
535.2988	535.2987	0.0001	0.2	11.88	1	a5 -H2O -H2O [7|1][1-6][5|6][1-5]	C24H38N8O6
535.2988	535.2987	0.0001	0.2	11.88	1	b5 -H2O [3|4][1-6][2|3][1-5]	C24H38N8O6
535.2988	535.2987	0.0001	0.2	11.88	1	c5 -H2O -NH3 [3|4][1-6][2|3][1-5]	C24H38N8O6
535.2988	535.2987	0.0001	0.2	11.88	1	a5 -H2O -H2O [5|6][1-6][2|3][1-5]	C24H38N8O6
535.2988	535.2987	0.0001	0.2	11.88	1	b5 -H2O [5|6][1-6][5|6][1-5]	C24H38N8O6
535.2988	535.2987	0.0001	0.2	11.88	1	c5 -H2O -NH3 [5|6][1-6][5|6][1-5]	C24H38N8O6
535.2988	535.2915	0.0073	13.7	11.88	1	c3 +CO -H2O -H2O [4|5][1-3]	C30H38N4O5
535.2988	535.2915	0.0073	13.7	11.88	1	b3 +CO -NH3 -NH3 [7|1][1-5][3|4][1-3]	C30H38N4O5
535.2988	535.2915	0.0073	13.7	11.88	1	b3 +CO -NH3 -NH3 [3|4][1-5][4|5][1-3]	C30H38N4O5
536.2940	536.2953	-0.0013	-2.5	8.07	1	b4 +H2O -C9H11O -NH3 [7|1][1-5][2|3][1-4]	C25H39N6O7
536.2940	536.2953	-0.0013	-2.5	8.07	1	b4 +H2O -C9H11O -NH3 [7|1][1-6][3|4][1-4]	C25H39N6O7
536.2940	536.2953	-0.0013	-2.5	8.07	1	b4 +H2O -C9H11O -NH3 [2|3][1-6][5|6][1-4]	C25H39N6O7
536.2940	536.2953	-0.0013	-2.5	8.07	1	b4 +H2O -C9H11O -NH3 [3|4][1-5][4|5][1-4]	C25H39N6O7
536.2940	536.2827	0.0113	21.0	8.07	1	z5 [4|5][3-7]	C24H37N7O7
536.2940	536.2827	0.0113	21.0	8.07	1	z5 [3|4][1-6][1|2][2-6]	C24H37N7O7
536.2940	536.2827	0.0113	21.0	8.07	1	z5 [5|6][1-6][4|5][2-6]	C24H37N7O7

Meas. m/z	Calc. m/z	δ (Da)	δ (ppm)	Rel. Int. (%)	z	Annotation	Formula
536.2940	536.2827	0.0113	21.0	8.07	1	b5 -NH3 [6|7][1-5]	C24H37N7O7
536.2940	536.2827	0.0113	21.0	8.07	1	c5 -NH3 -NH3 [6|7][1-5]	C24H37N7O7
536.2940	536.2827	0.0113	21.0	8.07	1	a5 -H2O -NH3 [7|1][1-6][5|6][1-5]	C24H37N7O7
536.2940	536.2827	0.0113	21.0	8.07	1	b5 -NH3 [3|4][1-6][2|3][1-5]	C24H37N7O7
536.2940	536.2827	0.0113	21.0	8.07	1	c5 -NH3 -NH3 [3|4][1-6][2|3][1-5]	C24H37N7O7
536.2940	536.2827	0.0113	21.0	8.07	1	a5 -H2O -NH3 [5|6][1-6][2|3][1-5]	C24H37N7O7
536.2940	536.2827	0.0113	21.0	8.07	1	b5 -NH3 [5|6][1-6][5|6][1-5]	C24H37N7O7
536.2940	536.2827	0.0113	21.0	8.07	1	c5 -NH3 -NH3 [5|6][1-6][5|6][1-5]	C24H37N7O7
552.3260	552.3378	-0.0118	-21.4	42.47	1	c4 -C9H11O [7|1][1-5][2|3][1-4]	C25H43N8O6
552.3260	552.3378	-0.0118	-21.4	42.47	1	c4 -C9H11O [7|1][1-6][3|4][1-4]	C25H43N8O6
552.3260	552.3378	-0.0118	-21.4	42.47	1	c4 -C9H11O [2|3][1-6][5|6][1-4]	C25H43N8O6
552.3260	552.3378	-0.0118	-21.4	42.47	1	c4 -C9H11O [3|4][1-5][4|5][1-4]	C25H43N8O6
552.3260	552.3253	0.0008	1.4	42.47	1	c5 -H2O [6|7][1-5]	C24H41N9O6
552.3260	552.3253	0.0008	1.4	42.47	1	c5 -H2O [3|4][1-6][2|3][1-5]	C24H41N9O6
552.3260	552.3253	0.0008	1.4	42.47	1	c5 -H2O [5|6][1-6][5|6][1-5]	C24H41N9O6
552.3260	552.3180	0.0080	14.5	42.47	1	b3 +CO -NH3 [3|4][1-5][4|5][1-3]	C30H41N5O5
552.3260	552.3180	0.0080	14.5	42.47	1	c3 +CO -NH3 -NH3 [3|4][1-5][4|5][1-3]	C30H41N5O5
552.3260	552.3180	0.0080	14.5	42.47	1	b3 +CO -NH3 [7|1][1-5][3|4][1-3]	C30H41N5O5
552.3260	552.3180	0.0080	14.5	42.47	1	c3 +CO -NH3 -NH3 [7|1][1-5][3|4][1-3]	C30H41N5O5
553.3122	553.3218	-0.0096	-17.4	60.29	1	b4 +H2O -C9H11O [3|4][1-5][4|5][1-4]	C25H42N7O7
553.3122	553.3218	-0.0096	-17.4	60.29	1	b4 +H2O -C9H11O [2|3][1-6][5|6][1-4]	C25H42N7O7
553.3122	553.3218	-0.0096	-17.4	60.29	1	b4 +H2O -C9H11O [7|1][1-5][2|3][1-4]	C25H42N7O7
553.3122	553.3218	-0.0096	-17.4	60.29	1	b4 +H2O -C9H11O [7|1][1-6][3|4][1-4]	C25H42N7O7
553.3122	553.3093	0.0029	5.3	60.29	1	b5 [6|7][1-5]	C24H40N8O7
553.3122	553.3093	0.0029	5.3	60.29	1	b5 [3|4][1-6][2|3][1-5]	C24H40N8O7
553.3122	553.3093	0.0029	5.3	60.29	1	b5 [5|6][1-6][5|6][1-5]	C24H40N8O7
553.3122	553.3093	0.0029	5.3	60.29	1	c5 -NH3 [6|7][1-5]	C24H40N8O7
553.3122	553.3093	0.0029	5.3	60.29	1	a5 -H2O [7|1][1-6][5|6][1-5]	C24H40N8O7
553.3122	553.3093	0.0029	5.3	60.29	1	c5 -NH3 [3|4][1-6][2|3][1-5]	C24H40N8O7
553.3122	553.3093	0.0029	5.3	60.29	1	a5 -H2O [5|6][1-6][2|3][1-5]	C24H40N8O7
553.3122	553.3093	0.0029	5.3	60.29	1	c5 -NH3 [5|6][1-6][5|6][1-5]	C24H40N8O7
553.3122	553.3021	0.0102	18.4	60.29	1	c3 -H2O -H2O [4|5][1-6][5|6][1-3]	C30H40N4O6
553.3122	553.3021	0.0102	18.4	60.29	1	c3 -H2O -H2O [2|3][1-4][2|3][1-3]	C30H40N4O6
553.3122	553.3021	0.0102	18.4	60.29	1	b3 +CO -CH3OH -CN2H2 [2|3][1-3]	C30H40N4O6
553.3122	553.3021	0.0102	18.4	60.29	1	b3 +CO -CH3OH -CN2H2 [3|4][1-3]	C30H40N4O6
553.3122	553.3021	0.0102	18.4	60.29	1	c3 +CO -CH3OH -CH5N3 [2|3][1-3]	C30H40N4O6
553.3122	553.3021	0.0102	18.4	60.29	1	c3 +CO -CH3OH -CH5N3 [3|4][1-3]	C30H40N4O6
553.3122	553.3021	0.0102	18.4	60.29	1	c3 +CO -H2O [4|5][1-3]	C30H40N4O6
570.3329	570.3358	-0.0029	-5.1	27.95	1	c5 [6|7][1-5]	C24H43N9O7
570.3329	570.3358	-0.0029	-5.1	27.95	1	c5 [5|6][1-6][5|6][1-5]	C24H43N9O7
570.3329	570.3358	-0.0029	-5.1	27.95	1	c5 [3|4][1-6][2|3][1-5]	C24H43N9O7
570.3329	570.3286	0.0043	7.5	27.95	1	x5 -C9H10O -CH5N3 [6|7][1-6][3|4][2-6]	C30H43N5O6
570.3329	570.3286	0.0043	7.5	27.95	1	c3 +CO -CH3OH -CN2H2 [2|3][1-3]	C30H43N5O6
570.3329	570.3286	0.0043	7.5	27.95	1	c3 +CO -CH3OH -CN2H2 [3|4][1-3]	C30H43N5O6
570.3329	570.3286	0.0043	7.5	27.95	1	b5 +CO -C9H10O -CHN3H6 [3|4][1-6][3|4][1-5]	C30H43N5O6
570.3329	570.3286	0.0043	7.5	27.95	1	b5 +CO -C9H10O -CHN3H6 [6|7][1-6][4|5][1-5]	C30H43N5O6
570.3329	570.3286	0.0043	7.5	27.95	1	x5 -C9H10O -CH5N3 [3|4][1-6][2|3][2-6]	C30H43N5O6
571.3507	571.3602	-0.0096	-16.8	36.05	1	a3 [2|3][1-3]	C30H46N6O5
571.3507	571.3602	-0.0096	-16.8	36.05	1	a3 [3|4][1-3]	C30H46N6O5
571.3507	571.3602	-0.0096	-16.8	36.05	1	b3 +H2O [3|4][1-4][3|4][1-3]	C30H46N6O5
571.3507	571.3602	-0.0096	-16.8	36.05	1	b3 +H2O [6|7][1-6][4|5][1-3]	C30H46N6O5
571.3507	571.3364	0.0142	24.9	36.05	1	a5 -C9H11O -CHN3H6 [1|2][1-6][2|3][1-5]	C30H44N5O6
571.3507	571.3364	0.0142	24.9	36.05	1	a5 -C9H11O -CHN3H6 [1|2][1-6][5|6][1-5]	C30H44N5O6
571.3507	571.3364	0.0142	24.9	36.05	1	a5 -C9H11O -CHN3H6 [3|4][1-6][5|6][1-5]	C30H44N5O6

Meas. m/z	Calc. m/z	δ (Da)	δ (ppm)	Rel. Int. (%)	z	Annotation	Formula
571.3507	571.3364	0.0142	24.9	36.05	1	a5 -C9H11O -CHN3H6 [6|7][1-6][2|3][1-5]	C30H44N5O6
571.3507	571.3364	0.0142	24.9	36.05	1	b5 +CO -C9H11O -CH5N3 [3|4][1-6][3|4][1-5]	C30H44N5O6
571.3507	571.3364	0.0142	24.9	36.05	1	b5 +CO -C9H11O -CH5N3 [6|7][1-6][4|5][1-5]	C30H44N5O6
582.3335	582.3286	0.0049	8.4	13.53	1	z3 [5|6][5-7]	C31H43N5O6
582.3335	582.3286	0.0049	8.4	13.53	1	z3 [6|7][5-7]	C31H43N5O6
582.3335	582.3286	0.0049	8.4	13.53	1	b3 -NH3 [2|3][1-3]	C31H43N5O6
582.3335	582.3286	0.0049	8.4	13.53	1	c3 -NH3 -NH3 [2|3][1-3]	C31H43N5O6
582.3335	582.3286	0.0049	8.4	13.53	1	b3 -NH3 [3|4][1-3]	C31H43N5O6
582.3335	582.3286	0.0049	8.4	13.53	1	c3 -NH3 -NH3 [3|4][1-3]	C31H43N5O6
582.3335	582.3286	0.0049	8.4	13.53	1	c4 -CH3OH [4|5][1-4]	C31H43N5O6
588.3320	588.3392	-0.0072	-12.2	4.49	1	b5 -C9H10O -CHN3H6 [7|1][1-5]	C30H45N5O7
588.3320	588.3392	-0.0072	-12.2	4.49	1	b5 -C9H10O -CHN3H6 [7|1][1-6][3|4][1-5]	C30H45N5O7
588.3320	588.3392	-0.0072	-12.2	4.49	1	a5 -C9H10O -H2O [1|2][1-6][3|4][1-5]	C30H45N5O7
588.3320	588.3392	-0.0072	-12.2	4.49	1	b5 -C9H10O -CHN3H6 [3|4][1-6][4|5][1-5]	C30H45N5O7
588.3320	588.3392	-0.0072	-12.2	4.49	1	a5 -C9H10O -H2O [4|5][1-6][4|5][1-5]	C30H45N5O7
588.3320	588.3266	0.0054	9.2	4.49	1	b4 +CO -C9H11O -NH3 [1|2][1-4]	C29H43N6O7
588.3320	588.3266	0.0054	9.2	4.49	1	b4 +CO -C9H11O -NH3 [1|2][1-5][2|3][1-4]	C29H43N6O7
588.3320	588.3266	0.0054	9.2	4.49	1	b4 +CO -C9H11O -NH3 [3|4][1-6][5|6][1-4]	C29H43N6O7
599.3530	599.3552	-0.0022	-3.6	100.00	1	c3 -NH3 [3|4][1-3]	C31H46N6O6
599.3530	599.3552	-0.0022	-3.6	100.00	1	b3 [3|4][1-3]	C31H46N6O6
599.3530	599.3552	-0.0022	-3.6	100.00	1	b3 [2|3][1-3]	C31H46N6O6
599.3530	599.3552	-0.0022	-3.6	100.00	1	c3 -NH3 [2|3][1-3]	C31H46N6O6
625.3283	625.3344	-0.0061	-9.7	7.55	1	x3 [5|6][5-7]	C32H44N6O7
625.3283	625.3344	-0.0061	-9.7	7.55	1	x3 [6|7][5-7]	C32H44N6O7
625.3283	625.3232	0.0051	8.2	7.55	1	b4 +CO [4|5][1-4]	C33H44N4O8
625.3283	625.3232	0.0051	8.2	7.55	1	c4 +CO -NH3 [4|5][1-4]	C33H44N4O8
636.3707	636.3841	-0.0134	-21.1	2.03	1	b5 +H2O -C9H11O -CN2H2 [1|2][1-6][2|3][1-5]	C31H51N6O8
636.3707	636.3841	-0.0134	-21.1	2.03	1	b5 +H2O -C9H11O -CN2H2 [1|2][1-6][5|6][1-5]	C31H51N6O8
636.3707	636.3841	-0.0134	-21.1	2.03	1	b5 +H2O -C9H11O -CN2H2 [3|4][1-6][5|6][1-5]	C31H51N6O8
636.3707	636.3841	-0.0134	-21.1	2.03	1	b5 +H2O -C9H11O -CN2H2 [6|7][1-6][2|3][1-5]	C31H51N6O8
636.3707	636.3841	-0.0134	-21.1	2.03	1	a5 -C9H11O -CN2H2 [1|2][1-5]	C31H51N6O8
636.3707	636.3828	-0.0121	-18.9	2.03	1	c5 -C9H10O [6|7][1-6][3|4][1-5]	C29H49N9O7
636.3707	636.3828	-0.0121	-18.9	2.03	1	c5 -C9H10O [3|4][1-5]	C29H49N9O7
636.3707	636.3828	-0.0121	-18.9	2.03	1	c5 -C9H10O [2|3][1-6][4|5][1-5]	C29H49N9O7
636.3707	636.3756	-0.0048	-7.6	2.03	1	c4 -CH3OH -CHN3H6 [3|4][1-6][5|6][1-4]	C35H49N5O6
636.3707	636.3756	-0.0048	-7.6	2.03	1	c4 +CO -H2O -H2O [7|1][1-5][4|5][1-4]	C35H49N5O6
636.3707	636.3756	-0.0048	-7.6	2.03	1	c4 +CO -H2O -H2O [7|1][1-6][4|5][1-4]	C35H49N5O6
636.3707	636.3756	-0.0048	-7.6	2.03	1	c4 +CO -H2O -H2O [4|5][1-5][3|4][1-4]	C35H49N5O6
636.3707	636.3756	-0.0048	-7.6	2.03	1	c4 +CO -H2O -H2O [4|5][1-6][3|4][1-4]	C35H49N5O6
636.3707	636.3756	-0.0048	-7.6	2.03	1	c4 -CH3OH -CHN3H6 [1|2][1-4]	C35H49N5O6
636.3707	636.3756	-0.0048	-7.6	2.03	1	c4 -CH3OH -CHN3H6 [1|2][1-5][2|3][1-4]	C35H49N5O6
636.3707	636.3643	0.0064	10.0	2.03	1	z4 -CH5N3 [3|4][1-6][3|4][3-6]	C36H49N3O7
636.3707	636.3643	0.0064	10.0	2.03	1	z4 -CN2H2 -NH3 [3|4][1-6][3|4][3-6]	C36H49N3O7
636.3707	636.3643	0.0064	10.0	2.03	1	b4 -CH5N3 -NH3 [3|4][1-6][5|6][1-4]	C36H49N3O7
636.3707	636.3643	0.0064	10.0	2.03	1	b4 -CH5N3 -NH3 [1|2][1-4]	C36H49N3O7
636.3707	636.3643	0.0064	10.0	2.03	1	z4 -CH5N3 [5|6][4-7]	C36H49N3O7
636.3707	636.3643	0.0064	10.0	2.03	1	z4 -CN2H2 -NH3 [5|6][4-7]	C36H49N3O7
636.3707	636.3643	0.0064	10.0	2.03	1	b4 -CH5N3 -NH3 [1|2][1-5][2|3][1-4]	C36H49N3O7
636.3707	636.3643	0.0064	10.0	2.03	1	z4 -CH5N3 [1|2][1-5][1|2][2-5]	C36H49N3O7
636.3707	636.3643	0.0064	10.0	2.03	1	z4 -CN2H2 -NH3 [1|2][1-5][1|2][2-5]	C36H49N3O7
636.3707	636.3590	0.0118	18.5	2.03	1	b5 +H2O -C9H11O [3|4][1-5]	C29H47N8O8
636.3707	636.3590	0.0118	18.5	2.03	1	b5 +H2O -C9H11O [2|3][1-6][4|5][1-5]	C29H47N8O8
636.3707	636.3590	0.0118	18.5	2.03	1	b5 +H2O -C9H11O [6|7][1-6][3|4][1-5]	C29H47N8O8
636.3707	636.3590	0.0118	18.5	2.03	1	a5 -C9H11O [7|1][1-6][2|3][1-5]	C29H47N8O8

Meas. m/z	Calc. m/z	δ (Da)	δ (ppm)	Rel. Int. (%)	z	Annotation	Formula
636.3707	636.3590	0.0118	18.5	2.03	1	a5 -C9H11O [2|3][1-6][5|6][1-5]	C29H47N8O8
637.3333	637.3304	0.0029	4.6	1.98	1	a6 -NH3 [5|6][1-6]	C28H44N8O9
637.3333	637.3232	0.0101	15.9	1.98	1	x4 -CH5N3 [2|3][1-6][3|4][3-6]	C34H44N4O8
637.3333	637.3232	0.0101	15.9	1.98	1	x4 -CN2H2 -NH3 [2|3][1-6][3|4][3-6]	C34H44N4O8
637.3333	637.3232	0.0101	15.9	1.98	1	x4 -CH5N3 [3|4][1-5][3|4][2-5]	C34H44N4O8
637.3333	637.3232	0.0101	15.9	1.98	1	x4 -CN2H2 -NH3 [3|4][1-5][3|4][2-5]	C34H44N4O8
637.3333	637.3232	0.0101	15.9	1.98	1	b4 -H2O [4|5][1-6][5|6][1-4]	C34H44N4O8
637.3333	637.3232	0.0101	15.9	1.98	1	c4 -H2O -NH3 [4|5][1-6][5|6][1-4]	C34H44N4O8
637.3333	637.3232	0.0101	15.9	1.98	1	b4 +CO -CHN3H6 [7|1][1-5][2|3][1-4]	C34H44N4O8
637.3333	637.3232	0.0101	15.9	1.98	1	c4 +CO -CHN3H6 -NH3 [7|1][1-5][2|3][1-4]	C34H44N4O8
637.3333	637.3232	0.0101	15.9	1.98	1	b4 +CO -CHN3H6 [7|1][1-6][3|4][1-4]	C34H44N4O8
637.3333	637.3232	0.0101	15.9	1.98	1	c4 +CO -CHN3H6 -NH3 [7|1][1-6][3|4][1-4]	C34H44N4O8
637.3333	637.3232	0.0101	15.9	1.98	1	b4 +CO -CHN3H6 [2|3][1-6][5|6][1-4]	C34H44N4O8
637.3333	637.3232	0.0101	15.9	1.98	1	c4 +CO -CHN3H6 -NH3 [2|3][1-6][5|6][1-4]	C34H44N4O8
637.3333	637.3232	0.0101	15.9	1.98	1	b4 +CO -CHN3H6 [3|4][1-5][4|5][1-4]	C34H44N4O8
637.3333	637.3232	0.0101	15.9	1.98	1	c4 +CO -CHN3H6 -NH3 [3|4][1-5][4|5][1-4]	C34H44N4O8
637.3333	637.3232	0.0101	15.9	1.98	1	b4 -CH3OH -CH5N3 [2|3][1-4]	C34H44N4O8
637.3333	637.3232	0.0101	15.9	1.98	1	z4 -CH3OH -CN2H2 [6|7][4-7]	C34H44N4O8
637.3333	637.3232	0.0101	15.9	1.98	1	x4 -CH5N3 [7|1][1-5][1|2][2-5]	C34H44N4O8
637.3333	637.3232	0.0101	15.9	1.98	1	x4 -CN2H2 -NH3 [7|1][1-5][1|2][2-5]	C34H44N4O8
637.3333	637.3232	0.0101	15.9	1.98	1	x4 -CH5N3 [7|1][1-6][1|2][3-6]	C34H44N4O8
637.3333	637.3232	0.0101	15.9	1.98	1	x4 -CN2H2 -NH3 [7|1][1-6][1|2][3-6]	C34H44N4O8
637.3333	637.3232	0.0101	15.9	1.98	1	b4 -H2O [2|3][1-5][2|3][1-4]	C34H44N4O8
637.3333	637.3232	0.0101	15.9	1.98	1	c4 -H2O -NH3 [2|3][1-5][2|3][1-4]	C34H44N4O8
637.3333	637.3192	0.0142	22.2	1.98	1	c5 +CO -C9H10O [2|3][1-6][2|3][1-5]	C29H44N6O10
637.3333	637.3192	0.0142	22.2	1.98	1	c5 +CO -C9H10O [4|5][1-6][5|6][1-5]	C29H44N6O10
655.3553	655.3701	-0.0148	-22.6	3.87	1	b4 +CO [4|5][1-6][3|4][1-4]	C35H50N4O8
655.3553	655.3701	-0.0148	-22.6	3.87	1	b4 +CO [4|5][1-5][3|4][1-4]	C35H50N4O8
655.3553	655.3701	-0.0148	-22.6	3.87	1	b4 +CO [7|1][1-6][4|5][1-4]	C35H50N4O8
655.3553	655.3701	-0.0148	-22.6	3.87	1	b4 +CO [7|1][1-5][4|5][1-4]	C35H50N4O8
655.3553	655.3701	-0.0148	-22.6	3.87	1	c4 +CO -NH3 [4|5][1-6][3|4][1-4]	C35H50N4O8
655.3553	655.3701	-0.0148	-22.6	3.87	1	c4 +CO -NH3 [4|5][1-5][3|4][1-4]	C35H50N4O8
655.3553	655.3701	-0.0148	-22.6	3.87	1	c4 +CO -NH3 [7|1][1-6][4|5][1-4]	C35H50N4O8
655.3553	655.3701	-0.0148	-22.6	3.87	1	c4 +CO -NH3 [7|1][1-5][4|5][1-4]	C35H50N4O8
655.3553	655.3576	-0.0022	-3.4	3.87	1	z6 -C9H11O -CH5N3 [2|3][2-7]	C34H48N5O8
655.3553	655.3576	-0.0022	-3.4	3.87	1	z6 -C9H11O -CH5N3 [5|6][2-7]	C34H48N5O8
657.3786	657.3858	-0.0072	-11.0	1.56	1	a4 [4|5][1-6][4|5][1-4]	C35H52N4O8
657.3786	657.3858	-0.0072	-11.0	1.56	1	a4 [1|2][1-5][3|4][1-4]	C35H52N4O8
657.3786	657.3858	-0.0072	-11.0	1.56	1	b4 +H2O [4|5][1-5][4|5][1-4]	C35H52N4O8
657.3786	657.3858	-0.0072	-11.0	1.56	1	b4 +H2O [1|2][1-6][3|4][1-4]	C35H52N4O8
657.3786	657.3719	0.0067	10.2	1.56	1	x5 -C9H10O -H2O [5|6][3-7]	C32H48N8O7
657.3786	657.3719	0.0067	10.2	1.56	1	x5 -C9H10O -H2O [3|4][1-6][3|4][2-6]	C32H48N8O7
657.3786	657.3719	0.0067	10.2	1.56	1	x5 -C9H10O -H2O [7|1][1-6][2|3][2-6]	C32H48N8O7
657.3786	657.3647	0.0139	21.2	1.56	1	z5 -H2O -H2O [2|3][3-7]	C38H48N4O6
657.3786	657.3647	0.0139	21.2	1.56	1	z5 -H2O -H2O [6|7][1-6][4|5][2-6]	C38H48N4O6
657.3786	657.3647	0.0139	21.2	1.56	1	z5 -H2O -H2O [4|5][1-6][1|2][2-6]	C38H48N4O6
665.3731	665.3869	-0.0138	-20.7	1.46	1	c5 -C9H10O -CH5N3 [1|2][1-5]	C32H52N6O9
665.3731	665.3869	-0.0138	-20.7	1.46	1	b5 -C9H10O -CN2H2 [1|2][1-5]	C32H52N6O9
665.3731	665.3770	-0.0039	-5.9	1.46	1	c4 +CO -CH3OH -H2O [7|1][1-6][3|4][1-4]	C34H48N8O6
665.3731	665.3770	-0.0039	-5.9	1.46	1	c4 +CO -CH3OH -H2O [2|3][1-6][5|6][1-4]	C34H48N8O6
665.3731	665.3770	-0.0039	-5.9	1.46	1	c4 +CO -CH3OH -H2O [3|4][1-5][4|5][1-4]	C34H48N8O6
665.3731	665.3770	-0.0039	-5.9	1.46	1	c4 +CO -CH3OH -H2O [7|1][1-5][2|3][1-4]	C34H48N8O6
665.3731	665.3657	0.0073	11.0	1.46	1	z4 [2|3][1-5][3|4][2-5]	C35H48N6O7
665.3731	665.3657	0.0073	11.0	1.46	1	z4 [7|1][4-7]	C35H48N6O7

Meas. m/z	Calc. m/z	δ (Da)	δ (ppm)	Rel. Int. (%)	z	Annotation	Formula
665.3731	665.3657	0.0073	11.0	1.46	1	c4 -NH3 -NH3 [6|7][1-6][3|4][1-4]	C35H48N6O7
665.3731	665.3657	0.0073	11.0	1.46	1	b4 -NH3 [6|7][1-6][3|4][1-4]	C35H48N6O7
665.3731	665.3657	0.0073	11.0	1.46	1	c4 -NH3 -NH3 [2|3][1-5][4|5][1-4]	C35H48N6O7
665.3731	665.3657	0.0073	11.0	1.46	1	b4 -NH3 [2|3][1-5][4|5][1-4]	C35H48N6O7
665.3731	665.3657	0.0073	11.0	1.46	1	c4 -NH3 -NH3 [3|4][1-4]	C35H48N6O7
665.3731	665.3657	0.0073	11.0	1.46	1	b4 -NH3 [3|4][1-4]	C35H48N6O7
665.3731	665.3657	0.0073	11.0	1.46	1	a4 -H2O -NH3 [2|3][1-4]	C35H48N6O7
665.3731	665.3657	0.0073	11.0	1.46	1	z4 [6|7][1-6][1|2][3-6]	C35H48N6O7
665.3731	665.3630	0.0100	15.0	1.46	1	b5 +H2O -C9H11O -CH5N3 [1|2][1-5]	C32H50N5O10
665.3731	665.3617	0.0114	17.1	1.46	1	c5 -C9H10O -NH3 [2|3][1-6][5|6][1-5]	C30H48N8O9
665.3731	665.3617	0.0114	17.1	1.46	1	b5 -C9H10O [2|3][1-6][5|6][1-5]	C30H48N8O9
665.3731	665.3617	0.0114	17.1	1.46	1	c5 -C9H10O -NH3 [7|1][1-6][2|3][1-5]	C30H48N8O9
665.3731	665.3617	0.0114	17.1	1.46	1	b5 -C9H10O [7|1][1-6][2|3][1-5]	C30H48N8O9
672.4021	672.3967	0.0054	8.0	1.46	1	c4 +CO [7|1][1-5][4|5][1-4]	C35H53N5O8
672.4021	672.3967	0.0054	8.0	1.46	1	c4 +CO [7|1][1-6][4|5][1-4]	C35H53N5O8
672.4021	672.3967	0.0054	8.0	1.46	1	c4 +CO [4|5][1-5][3|4][1-4]	C35H53N5O8
672.4021	672.3967	0.0054	8.0	1.46	1	c4 +CO [4|5][1-6][3|4][1-4]	C35H53N5O8
682.3889	682.3923	-0.0034	-5.0	14.86	1	a4 -H2O [2|3][1-4]	C35H51N7O7
682.3889	682.3923	-0.0034	-5.0	14.86	1	b4 [6|7][1-6][3|4][1-4]	C35H51N7O7
682.3889	682.3923	-0.0034	-5.0	14.86	1	c4 -NH3 [3|4][1-4]	C35H51N7O7
682.3889	682.3923	-0.0034	-5.0	14.86	1	c4 -NH3 [2|3][1-5][4|5][1-4]	C35H51N7O7
682.3889	682.3923	-0.0034	-5.0	14.86	1	c4 -NH3 [6|7][1-6][3|4][1-4]	C35H51N7O7
682.3889	682.3923	-0.0034	-5.0	14.86	1	b4 [3|4][1-4]	C35H51N7O7
682.3889	682.3923	-0.0034	-5.0	14.86	1	b4 [2|3][1-5][4|5][1-4]	C35H51N7O7
682.3889	682.3896	-0.0007	-1.0	14.86	1	b5 +H2O -C9H11O -CN2H2 [1|2][1-5]	C32H53N6O10
682.3889	682.3883	0.0006	0.9	14.86	1	c5 -C9H10O [7|1][1-6][2|3][1-5]	C30H51N9O9
682.3889	682.3883	0.0006	0.9	14.86	1	c5 -C9H10O [2|3][1-6][5|6][1-5]	C30H51N9O9
710.3874	710.3872	0.0002	0.3	7.20	1	b4 -H2O [2|3][1-4]	C36H51N7O8
710.3874	710.3872	0.0002	0.3	7.20	1	c4 -H2O -NH3 [2|3][1-4]	C36H51N7O8
710.3874	710.3872	0.0002	0.3	7.20	1	b4 +CO [2|3][1-5][4|5][1-4]	C36H51N7O8
710.3874	710.3872	0.0002	0.3	7.20	1	b4 +CO [3|4][1-4]	C36H51N7O8
710.3874	710.3872	0.0002	0.3	7.20	1	b4 +CO [6|7][1-6][3|4][1-4]	C36H51N7O8
710.3874	710.3872	0.0002	0.3	7.20	1	c4 +CO -NH3 [3|4][1-4]	C36H51N7O8
710.3874	710.3872	0.0002	0.3	7.20	1	c4 +CO -NH3 [2|3][1-5][4|5][1-4]	C36H51N7O8
710.3874	710.3872	0.0002	0.3	7.20	1	c4 +CO -NH3 [6|7][1-6][3|4][1-4]	C36H51N7O8
710.3874	710.3832	0.0043	6.0	7.20	1	c5 +CO -C9H10O [7|1][1-6][2|3][1-5]	C31H51N9O10
710.3874	710.3832	0.0043	6.0	7.20	1	c5 +CO -C9H10O [2|3][1-6][5|6][1-5]	C31H51N9O10
710.3874	710.3760	0.0115	16.1	7.20	1	a5 -CHN3H6 [7|1][1-6][2|3][1-5]	C37H51N5O9
710.3874	710.3760	0.0115	16.1	7.20	1	a5 -CHN3H6 [2|3][1-6][5|6][1-5]	C37H51N5O9
710.3874	710.3760	0.0115	16.1	7.20	1	b5 +H2O -CHN3H6 [3|4][1-5]	C37H51N5O9
710.3874	710.3760	0.0115	16.1	7.20	1	b5 +H2O -CHN3H6 [2|3][1-6][4|5][1-5]	C37H51N5O9
710.3874	710.3760	0.0115	16.1	7.20	1	b5 +H2O -CHN3H6 [6|7][1-6][3|4][1-5]	C37H51N5O9
711.4020	711.4076	-0.0056	-7.8	7.38	1	c5 -CN2H2 -NH3 [3|4][1-5]	C37H54N6O8
711.4020	711.4076	-0.0056	-7.8	7.38	1	a5 -CN2H2 -H2O [7|1][1-6][2|3][1-5]	C37H54N6O8
711.4020	711.4076	-0.0056	-7.8	7.38	1	b5 -CN2H2 [2|3][1-6][4|5][1-5]	C37H54N6O8
711.4020	711.4076	-0.0056	-7.8	7.38	1	c5 -CH5N3 [2|3][1-6][4|5][1-5]	C37H54N6O8
711.4020	711.4076	-0.0056	-7.8	7.38	1	c5 -CN2H2 -NH3 [2|3][1-6][4|5][1-5]	C37H54N6O8
711.4020	711.4076	-0.0056	-7.8	7.38	1	a5 -CN2H2 -H2O [2|3][1-6][5|6][1-5]	C37H54N6O8
711.4020	711.4076	-0.0056	-7.8	7.38	1	b5 -CN2H2 [6|7][1-6][3|4][1-5]	C37H54N6O8
711.4020	711.4076	-0.0056	-7.8	7.38	1	c5 -CH5N3 [6|7][1-6][3|4][1-5]	C37H54N6O8
711.4020	711.4076	-0.0056	-7.8	7.38	1	c5 -CN2H2 -NH3 [6|7][1-6][3|4][1-5]	C37H54N6O8
711.4020	711.4076	-0.0056	-7.8	7.38	1	c5 -CH5N3 [3|4][1-5]	C37H54N6O8
711.4020	711.4076	-0.0056	-7.8	7.38	1	b5 -CN2H2 [3|4][1-5]	C37H54N6O8
711.4020	711.3964	0.0057	8.0	7.38	1	a5 -NH3 [7|1][1-6][4|5][1-5]	C38H54N4O9

Meas. m/z	Calc. m/z	δ (Da)	δ (ppm)	Rel. Int. (%)	z	Annotation	Formula
711.4020	711.3964	0.0057	8.0	7.38	1	a5 -NH3 [4|5][1-6][3|4][1-5]	C38H54N4O9
711.4020	711.3964	0.0057	8.0	7.38	1	b5 +H2O -NH3 [4|5][1-5]	C38H54N4O9
711.4020	711.3964	0.0057	8.0	7.38	1	b5 +H2O -NH3 [4|5][1-6][2|3][1-5]	C38H54N4O9
711.4020	711.3964	0.0057	8.0	7.38	1	b5 +H2O -NH3 [6|7][1-6][5|6][1-5]	C38H54N4O9
728.3914	728.3978	-0.0063	-8.7	8.98	1	b4 [2|3][1-4]	C36H53N7O9
728.3914	728.3978	-0.0063	-8.7	8.98	1	c4 -NH3 [2|3][1-4]	C36H53N7O9
729.4095	729.4182	-0.0087	-11.9	5.02	1	b5 +H2O -CN2H2 [2|3][1-6][4|5][1-5]	C37H56N6O9
729.4095	729.4182	-0.0087	-11.9	5.02	1	b5 +H2O -CN2H2 [6|7][1-6][3|4][1-5]	C37H56N6O9
729.4095	729.4182	-0.0087	-11.9	5.02	1	a5 -CN2H2 [7|1][1-6][2|3][1-5]	C37H56N6O9
729.4095	729.4182	-0.0087	-11.9	5.02	1	a5 -CN2H2 [2|3][1-6][5|6][1-5]	C37H56N6O9
729.4095	729.4182	-0.0087	-11.9	5.02	1	b5 +H2O -CN2H2 [3|4][1-5]	C37H56N6O9
729.4095	729.3970	0.0124	17.1	5.02	1	z5 -CH3OH -NH3 [1|2][1-6][1|2][2-6]	C40H52N6O7
729.4095	729.3970	0.0124	17.1	5.02	1	z5 -CH3OH -NH3 [1|2][1-6][4|5][2-6]	C40H52N6O7
729.4095	729.3970	0.0124	17.1	5.02	1	x5 -NH3 -NH3 [3|4][1-6][2|3][2-6]	C40H52N6O7
729.4095	729.3970	0.0124	17.1	5.02	1	z5 -CH3OH -NH3 [3|4][1-6][4|5][2-6]	C40H52N6O7
729.4095	729.3970	0.0124	17.1	5.02	1	z5 -CH3OH -NH3 [6|7][1-6][1|2][2-6]	C40H52N6O7
729.4095	729.3970	0.0124	17.1	5.02	1	x5 -NH3 -NH3 [6|7][1-6][3|4][2-6]	C40H52N6O7
738.3820	738.3821	-0.0001	-0.1	4.12	1	b4 +CO -H2O [2|3][1-4]	C37H51N7O9
738.3820	738.3821	-0.0001	-0.1	4.12	1	c4 +CO -H2O -NH3 [2|3][1-4]	C37H51N7O9
738.3820	738.3709	0.0112	15.1	4.12	1	b5 -CHN3H6 [7|1][1-6][2|3][1-5]	C38H51N5O10
738.3820	738.3709	0.0112	15.1	4.12	1	c5 -CHN3H6 -NH3 [7|1][1-6][2|3][1-5]	C38H51N5O10
738.3820	738.3709	0.0112	15.1	4.12	1	b5 -CHN3H6 [2|3][1-6][5|6][1-5]	C38H51N5O10
738.3820	738.3709	0.0112	15.1	4.12	1	c5 -CHN3H6 -NH3 [2|3][1-6][5|6][1-5]	C38H51N5O10
738.3820	738.3709	0.0112	15.1	4.12	1	b5 +H2O -CH3OH -CH5N3 [2|3][1-5]	C38H51N5O10
783.4823	783.4763	0.0060	7.6	5.15	1	b5 [7|1][1-5]	C40H62N8O8
783.4823	783.4763	0.0060	7.6	5.15	1	b5 [7|1][1-6][3|4][1-5]	C40H62N8O8
783.4823	783.4763	0.0060	7.6	5.15	1	b5 [3|4][1-6][4|5][1-5]	C40H62N8O8
783.4823	783.4763	0.0060	7.6	5.15	1	c5 -NH3 [7|1][1-5]	C40H62N8O8
783.4823	783.4763	0.0060	7.6	5.15	1	c5 -NH3 [7|1][1-6][3|4][1-5]	C40H62N8O8
783.4823	783.4763	0.0060	7.6	5.15	1	c5 -NH3 [3|4][1-6][4|5][1-5]	C40H62N8O8
793.4653	793.4607	0.0046	5.8	1.23	1	b5 +CO -H2O [7|1][1-5]	C41H60N8O8
793.4653	793.4607	0.0046	5.8	1.23	1	c5 +CO -H2O -NH3 [7|1][1-5]	C41H60N8O8
793.4653	793.4607	0.0046	5.8	1.23	1	b5 +CO -H2O [7|1][1-6][3|4][1-5]	C41H60N8O8
793.4653	793.4607	0.0046	5.8	1.23	1	c5 +CO -H2O -NH3 [7|1][1-6][3|4][1-5]	C41H60N8O8
793.4653	793.4607	0.0046	5.8	1.23	1	b5 +CO -H2O [3|4][1-6][4|5][1-5]	C41H60N8O8
793.4653	793.4607	0.0046	5.8	1.23	1	c5 +CO -H2O -NH3 [3|4][1-6][4|5][1-5]	C41H60N8O8
800.4589	800.4440	0.0149	18.6	1.56	1	b5 +H2O -CH5N3 [1|2][1-5]	C41H61N5O11
800.4589	800.4440	0.0149	18.6	1.56	1	b5 +H2O -CN2H2 -NH3 [1|2][1-5]	C41H61N5O11
838.5149	838.5185	-0.0036	-4.3	5.17	1	a6 [3|4][1-6]	C43H67N9O8
838.5149	838.5185	-0.0036	-4.3	5.17	1	a6 [6|7][1-6]	C43H67N9O8
844.4580	844.4716	-0.0136	-16.1	4.51	1	b6 +CO -CH3OH -H2O [3|4][1-6]	C44H61N9O8
844.4580	844.4716	-0.0136	-16.1	4.51	1	b6 +CO -CH3OH -H2O [6|7][1-6]	C44H61N9O8
844.4580	844.4563	0.0017	2.0	4.51	1	M -C9H10O -NH3 [7|1][1-7]	C40H61N9O11
844.4580	844.4563	0.0017	2.0	4.51	1	c5 +CO [7|1][1-6][2|3][1-5]	C40H61N9O11
844.4580	844.4563	0.0017	2.0	4.51	1	c5 +CO [2|3][1-6][5|6][1-5]	C40H61N9O11
845.4701	845.4556	0.0145	17.2	3.50	1	b6 +CO -CH3OH -NH3 [6|7][1-6]	C44H60N8O9
845.4701	845.4556	0.0145	17.2	3.50	1	z6 -CH3OH -H2O [6|7][2-7]	C44H60N8O9
845.4701	845.4556	0.0145	17.2	3.50	1	b6 +CO -CH3OH -NH3 [3|4][1-6]	C44H60N8O9
861.4821	861.4869	-0.0048	-5.6	2.99	1	a6 -H2O -NH3 [1|2][1-6]	C45H64N8O9
861.4821	861.4829	-0.0008	-0.9	2.99	1	M -C9H10O [7|1][1-7]	C40H64N10O11
862.4820	862.4822	-0.0001	-0.2	3.45	1	b6 -CH3OH -H2O [7|1][1-6]	C44H63N9O9
862.4820	862.4822	-0.0001	-0.2	3.45	1	c6 +CO -CH3OH -NH3 [3|4][1-6]	C44H63N9O9
862.4820	862.4822	-0.0001	-0.2	3.45	1	b6 +CO -CH3OH [3|4][1-6]	C44H63N9O9
862.4820	862.4822	-0.0001	-0.2	3.45	1	b6 +CO -CH3OH [6|7][1-6]	C44H63N9O9

Meas. m/z	Calc. m/z	δ (Da)	δ (ppm)	Rel. Int. (%)	z	Annotation	Formula
862.4820	862.4822	-0.0001	-0.2	3.45	1	c6 +CO -CH3OH -NH3 [6|7][1-6]	C44H63N9O9
862.4820	862.4709	0.0111	12.9	3.45	1	c6 -CHN3H6 -H2O [1|2][1-6]	C45H63N7O10
862.4820	862.4709	0.0111	12.9	3.45	1	a6 -NH3 -NH3 [1|2][1-6]	C45H63N7O10
865.5212	865.5294	-0.0083	-9.5	7.37	1	c6 -H2O [3|4][1-6]	C44H68N10O8
865.5212	865.5294	-0.0083	-9.5	7.37	1	c6 -H2O [6|7][1-6]	C44H68N10O8
866.5164	866.5135	0.0030	3.5	18.91	1	b6 [6|7][1-6]	C44H67N9O9
866.5164	866.5135	0.0030	3.5	18.91	1	b6 [3|4][1-6]	C44H67N9O9
866.5164	866.5135	0.0030	3.5	18.91	1	a6 -H2O [7|1][1-6]	C44H67N9O9
866.5164	866.5135	0.0030	3.5	18.91	1	c6 -NH3 [3|4][1-6]	C44H67N9O9
866.5164	866.5135	0.0030	3.5	18.91	1	c6 -NH3 [6|7][1-6]	C44H67N9O9
884.5304	884.5240	0.0064	7.2	3.65	1	b6 +H2O [3|4][1-6]	C44H69N9O10
884.5304	884.5240	0.0064	7.2	3.65	1	a6 [7|1][1-6]	C44H69N9O10
884.5304	884.5240	0.0064	7.2	3.65	1	b6 +H2O [6|7][1-6]	C44H69N9O10
910.5136	910.5033	0.0103	11.3	4.98	1	b6 +H2O -CH3OH [1|2][1-6]	C45H67N9O11

Generated by mMass • Open Source Mass Spectrometry Tool • www.mmass.org

mMass Report: *cycloA_TOF_filtered_ms3*

**Date**	Mon Oct 24 16:08:57 2011	**Scan Number**	
**Operator**		**Retention Time**	
**Contact**	http://bix.ucsd.edu/nrp/	**MS Level**	
**Institution**		**Precursor m/z**	
**Instrument**		**Polarity**	unknown
		**Spectrum Points**	0
		**Peak List**	99

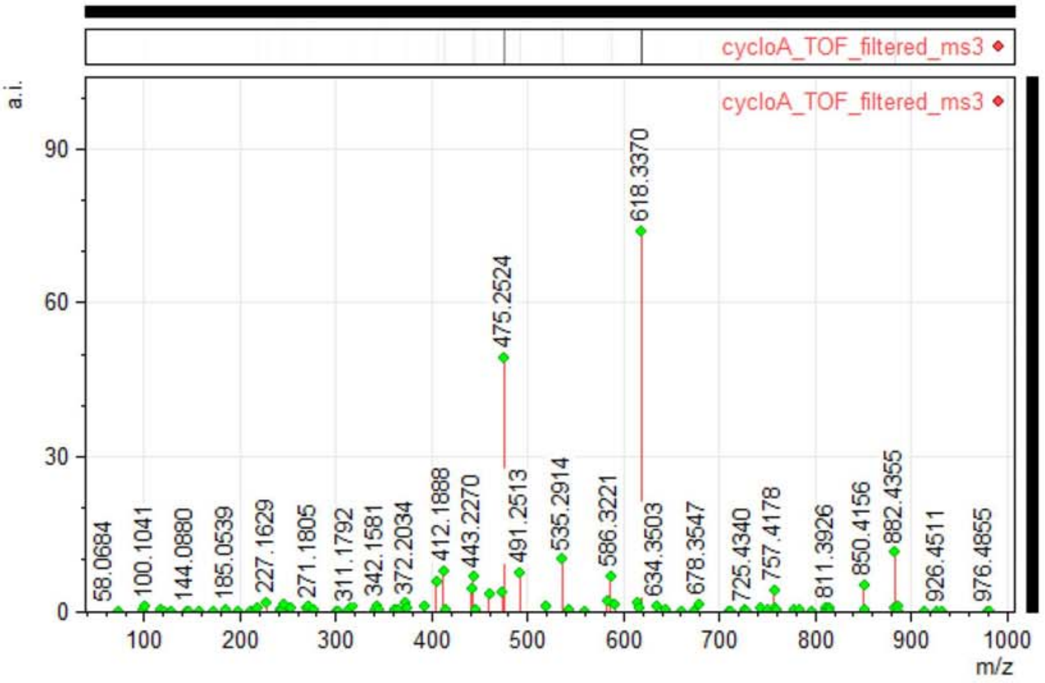

Sequence - *Cyclomarin A*

Accession	Length	Mo. Mass	Av. Mass	Coverage	Matched Int.
	7 (Cyclic)	1042.6103	1043.2998	100.0 %	99.7 %

Ala | b-OMe-Phe | Val | NMe-Leu | NH2-diCH3-hex | diMe-epoxprop-OH-Trp | NMe-OH-Leu

Meas. m/z	Calc. m/z	δ (Da)	δ (ppm)	Rel. Int. (%)	z	Annotation	Formula
72.0719	72.0808	-0.0089	-123.1	0.05	1	a1 [2|3][1-1]	C4H9N
72.0719	72.0444	0.0275	381.8	0.05	1	c1 -NH3 [7|1][1-1]	C3H5NO
72.0719	72.0444	0.0275	381.8	0.05	1	b1 [7|1][1-1]	C3H5NO
98.0868	98.0964	-0.0096	-98.1	0.56	1	a1 -H2O [6|7][1-1]	C6H11N
98.0868	98.0237	0.0631	644.2	0.56	1	x1 [1|2][7-7]	C4H3NO2
100.1041	100.1121	-0.0080	-79.7	1.25	1	a1 [3|4][1-1]	C6H13N
100.1041	100.0757	0.0284	283.9	1.25	1	b1 [2|3][1-1]	C5H9NO
100.1041	100.0757	0.0284	283.9	1.25	1	c1 -NH3 [2|3][1-1]	C5H9NO
100.1041	100.0393	0.0648	647.7	1.25	1	b1 +CO [7|1][1-1]	C4H5NO2
100.1041	100.0393	0.0648	647.7	1.25	1	c1 +CO -NH3 [7|1][1-1]	C4H5NO2
116.1009	116.1070	-0.0061	-52.5	0.49	1	a1 [6|7][1-1]	C6H13NO
121.0589	121.0648	-0.0059	-48.7	0.17	1	b1 +CO -H2O -NH3 [3|4][1-1]	C8H8O

Meas. m/z	Calc. m/z	δ (Da)	δ (ppm)	Rel. Int. (%)	z	Annotation	Formula
128.1036	128.1070	-0.0034	-26.5	0.06	1	c1 -NH3 [3|4][1-1]	C7H13NO
128.1036	128.1070	-0.0034	-26.5	0.06	1	b1 [3|4][1-1]	C7H13NO
128.1036	128.0706	0.0330	257.6	0.06	1	b1 +CO [2|3][1-1]	C6H9NO2
128.1036	128.0706	0.0330	257.6	0.06	1	c1 +CO -NH3 [2|3][1-1]	C6H9NO2
144.0880	144.1019	-0.0139	-96.5	0.14	1	c1 -NH3 [6|7][1-1]	C7H13NO2
144.0880	144.1019	-0.0139	-96.5	0.14	1	b1 [6|7][1-1]	C7H13NO2
146.0495	146.1176	-0.0681	-465.8	0.08	1	b1 +H2O [3|4][1-1]	C7H15NO2
146.0495	146.0964	-0.0469	-321.2	0.08	1	z2 -H2O -H2O [7|1][1-4][1|2][3-4]	C10H11N
146.0495	146.0964	-0.0469	-321.2	0.08	1	z2 -H2O -H2O [3|4][1-5][1|2][4-5]	C10H11N
156.0849	156.1019	-0.0170	-108.9	0.13	1	b1 +CO [3|4][1-1]	C8H13NO2
156.0849	156.1019	-0.0170	-108.9	0.13	1	c1 +CO -NH3 [3|4][1-1]	C8H13NO2
172.0856	172.0968	-0.0112	-65.2	0.19	1	b2 +H2O -NH3 [2|3][1-6][5|6][1-2]	C8H13NO3
172.0856	172.0968	-0.0112	-65.2	0.19	1	b1 +CO [6|7][1-1]	C8H13NO3
172.0856	172.0968	-0.0112	-65.2	0.19	1	b2 +H2O -NH3 [7|1][1-3][2|3][1-2]	C8H13NO3
172.0856	172.0968	-0.0112	-65.2	0.19	1	c1 +CO -NH3 [6|7][1-1]	C8H13NO3
185.0539	185.1285	-0.0746	-402.7	0.50	1	c1 +CO [4|5][1-1]	C9H16N2O2
197.1073	197.1648	-0.0575	-291.8	0.22	1	a2 -H2O [6|7][1-4][3|4][1-2]	C11H20N2O
197.1073	197.1648	-0.0575	-291.8	0.22	1	a2 -H2O [2|3][1-5][4|5][1-2]	C11H20N2O
197.1073	197.1285	-0.0212	-107.3	0.22	1	c2 -H2O -NH3 [6|7][1-2]	C10H16N2O2
197.1073	197.1285	-0.0212	-107.3	0.22	1	b2 -H2O [6|7][1-2]	C10H16N2O2
197.1073	197.0921	0.0152	77.3	0.22	1	x2 [2|3][1-6][1|2][5-6]	C9H12N2O3
197.1073	197.0921	0.0152	77.3	0.22	1	x2 [7|1][1-3][1|2][2-3]	C9H12N2O3
197.1073	197.0597	0.0476	241.5	0.22	1	z2 -H2O -NH3 [2|3][6-7]	C13H8O2
210.1445	210.1601	-0.0156	-74.2	0.22	1	c2 -H2O [7|1][1-5][4|5][1-2]	C11H19N3O
210.1445	210.1601	-0.0156	-74.2	0.22	1	c2 -H2O [4|5][1-4][3|4][1-2]	C11H19N3O
210.1445	210.1489	-0.0044	-20.7	0.22	1	b2 -NH3 [2|3][1-2]	C12H19NO2
210.1445	210.1489	-0.0044	-20.7	0.22	1	z2 [4|5][6-7]	C12H19NO2
210.1445	210.1125	0.0320	152.4	0.22	1	b2 +CO -NH3 [3|4][1-5][4|5][1-2]	C11H15NO3
210.1445	210.1125	0.0320	152.4	0.22	1	b2 +CO -NH3 [7|1][1-4][3|4][1-2]	C11H15NO3
215.1293	215.1754	-0.0461	-214.3	0.28	1	a2 [6|7][1-4][3|4][1-2]	C11H22N2O2
215.1293	215.1754	-0.0461	-214.3	0.28	1	a2 [2|3][1-5][4|5][1-2]	C11H22N2O2
215.1293	215.1430	-0.0137	-63.9	0.28	1	z2 -H2O -NH3 [5|6][6-7]	C15H18O
215.1293	215.1390	-0.0097	-45.2	0.28	1	c2 -NH3 [6|7][1-2]	C10H18N2O3
215.1293	215.1390	-0.0097	-45.2	0.28	1	b2 [6|7][1-2]	C10H18N2O3
215.1293	215.0703	0.0590	274.5	0.28	1	z2 -NH3 [2|3][6-7]	C13H10O3
217.0955	217.1547	-0.0592	-272.5	1.04	1	b2 +H2O [3|4][1-5][4|5][1-2]	C10H20N2O3
217.0955	217.1547	-0.0592	-272.5	1.04	1	b2 +H2O [7|1][1-4][3|4][1-2]	C10H20N2O3
217.0955	217.1335	-0.0380	-175.2	1.04	1	x2 -H2O -H2O [4|5][6-7]	C13H16N2O
227.1629	227.1754	-0.0125	-55.0	2.45	1	b2 [2|3][1-2]	C12H22N2O2
227.1629	227.1754	-0.0125	-55.0	2.45	1	c2 -NH3 [2|3][1-2]	C12H22N2O2
227.1629	227.1390	0.0239	105.1	2.45	1	b2 +CO [7|1][1-4][3|4][1-2]	C11H18N2O3
227.1629	227.1390	0.0239	105.1	2.45	1	c2 +CO -NH3 [7|1][1-4][3|4][1-2]	C11H18N2O3
227.1629	227.1390	0.0239	105.1	2.45	1	b2 +CO [3|4][1-5][4|5][1-2]	C11H18N2O3
227.1629	227.1390	0.0239	105.1	2.45	1	c2 +CO -NH3 [3|4][1-5][4|5][1-2]	C11H18N2O3
241.1203	241.1699	-0.0496	-205.8	0.55	1	a2 -H2O -H2O [1|2][1-3][2|3][1-2]	C16H20N2
241.1203	241.1699	-0.0496	-205.8	0.55	1	a2 -H2O -H2O [3|4][1-6][5|6][1-2]	C16H20N2
241.1203	241.1335	-0.0132	-54.9	0.55	1	a1 -H2O [5|6][1-1]	C15H16N2O
241.1203	241.1183	0.0020	8.4	0.55	1	x2 [1|2][6-7]	C11H16N2O4
243.1885	243.2067	-0.0182	-74.9	0.50	1	a2 [3|4][1-4][3|4][1-2]	C13H26N2O2
243.1885	243.2067	-0.0182	-74.9	0.50	1	a2 [6|7][1-5][4|5][1-2]	C13H26N2O2
243.1885	243.1703	0.0182	74.8	0.50	1	b2 [2|3][1-5][4|5][1-2]	C12H22N2O3
243.1885	243.1703	0.0182	74.8	0.50	1	c2 -NH3 [2|3][1-5][4|5][1-2]	C12H22N2O3
243.1885	243.1703	0.0182	74.8	0.50	1	b2 [6|7][1-4][3|4][1-2]	C12H22N2O3
243.1885	243.1703	0.0182	74.8	0.50	1	c2 -NH3 [6|7][1-4][3|4][1-2]	C12H22N2O3

Meas. m/z	Calc. m/z	δ (Da)	δ (ppm)	Rel. Int. (%)	z	Annotation	Formula
243.1885	243.1339	0.0546	224.4	0.50	1	b2 +CO [6|7][1-2]	C11H18N2O4
243.1885	243.1339	0.0546	224.4	0.50	1	c2 +CO -NH3 [6|7][1-2]	C11H18N2O4
243.1885	243.1016	0.0869	357.6	0.50	1	z2 -NH3 [3|4][6-7]	C15H14O3
245.1127	245.1860	-0.0733	-298.8	1.97	1	b2 +H2O [2|3][1-2]	C12H24N2O3
245.1127	245.1648	-0.0521	-212.7	1.97	1	z3 -H2O -H2O [2|3][1-6][2|3][4-6]	C15H20N2O
245.1127	245.1648	-0.0521	-212.7	1.97	1	z3 -H2O -H2O [7|1][1-4][1|2][2-4]	C15H20N2O
253.1422	253.1911	-0.0489	-193.0	1.15	1	a3 -NH3 [7|1][1-4][2|3][1-3]	C14H24N2O2
253.1422	253.1911	-0.0489	-193.0	1.15	1	a3 -NH3 [2|3][1-6][5|6][1-3]	C14H24N2O2
253.1422	253.1911	-0.0489	-193.0	1.15	1	b2 -H2O [6|7][1-5][4|5][1-2]	C14H24N2O2
253.1422	253.1911	-0.0489	-193.0	1.15	1	c2 -H2O -NH3 [3|4][1-4][3|4][1-2]	C14H24N2O2
253.1422	253.1911	-0.0489	-193.0	1.15	1	b2 -H2O [3|4][1-4][3|4][1-2]	C14H24N2O2
253.1422	253.1911	-0.0489	-193.0	1.15	1	c2 -H2O -NH3 [6|7][1-5][4|5][1-2]	C14H24N2O2
253.1422	253.1547	-0.0125	-49.3	1.15	1	x2 [4|5][6-7]	C13H20N2O3
253.1422	253.1547	-0.0125	-49.3	1.15	1	c2 +CO -H2O -NH3 [2|3][1-5][4|5][1-2]	C13H20N2O3
253.1422	253.1547	-0.0125	-49.3	1.15	1	b2 +CO -H2O [2|3][1-5][4|5][1-2]	C13H20N2O3
253.1422	253.1547	-0.0125	-49.3	1.15	1	c2 +CO -H2O -NH3 [6|7][1-4][3|4][1-2]	C13H20N2O3
253.1422	253.1547	-0.0125	-49.3	1.15	1	b2 +CO -H2O [6|7][1-4][3|4][1-2]	C13H20N2O3
253.1422	253.1223	0.0199	78.6	1.15	1	z2 -H2O -NH3 [1|2][1-3][1|2][2-3]	C17H16O2
253.1422	253.1223	0.0199	78.6	1.15	1	z2 -H2O -NH3 [3|4][1-6][1|2][5-6]	C17H16O2
253.1422	253.0859	0.0563	222.4	1.15	1	z1 -NH3 [6|7][7-7]	C16H12O3
269.1133	269.1860	-0.0727	-270.0	1.09	1	a3 -NH3 [2|3][1-6][4|5][1-3]	C14H24N2O3
269.1133	269.1860	-0.0727	-270.0	1.09	1	a3 -NH3 [6|7][1-4][3|4][1-3]	C14H24N2O3
269.1133	269.1648	-0.0515	-191.5	1.09	1	b2 -H2O -H2O [1|2][1-3][2|3][1-2]	C17H20N2O
269.1133	269.1648	-0.0515	-191.5	1.09	1	b2 -H2O -H2O [3|4][1-6][5|6][1-2]	C17H20N2O
269.1133	269.1496	-0.0363	-134.8	1.09	1	x2 [6|7][1-4][1|2][3-4]	C13H20N2O4
269.1133	269.1496	-0.0363	-134.8	1.09	1	x2 [2|3][1-5][1|2][4-5]	C13H20N2O4
269.1133	269.1285	-0.0152	-56.3	1.09	1	b1 -H2O [5|6][1-1]	C16H16N2O2
269.1133	269.1285	-0.0152	-56.3	1.09	1	c1 -H2O -NH3 [5|6][1-1]	C16H16N2O2
269.1133	269.1172	-0.0039	-14.6	1.09	1	z2 -H2O -NH3 [1|2][1-6][1|2][5-6]	C17H16O3
269.1133	269.1172	-0.0039	-14.6	1.09	1	z2 -H2O -NH3 [6|7][1-3][1|2][2-3]	C17H16O3
271.1805	271.2016	-0.0211	-77.9	1.26	1	c2 -NH3 [3|4][1-4][3|4][1-2]	C14H26N2O3
271.1805	271.2016	-0.0211	-77.9	1.26	1	b2 [3|4][1-4][3|4][1-2]	C14H26N2O3
271.1805	271.2016	-0.0211	-77.9	1.26	1	b2 [6|7][1-5][4|5][1-2]	C14H26N2O3
271.1805	271.2016	-0.0211	-77.9	1.26	1	c2 -NH3 [6|7][1-5][4|5][1-2]	C14H26N2O3
271.1805	271.1805	0.0000	0.0	1.26	1	a2 -H2O [1|2][1-4][3|4][1-2]	C17H22N2O
271.1805	271.1805	0.0000	0.0	1.26	1	a2 -H2O [4|5][1-5][4|5][1-2]	C17H22N2O
271.1805	271.1652	0.0153	56.3	1.26	1	c2 +CO -NH3 [6|7][1-4][3|4][1-2]	C13H22N2O4
271.1805	271.1652	0.0153	56.3	1.26	1	b2 +CO [6|7][1-4][3|4][1-2]	C13H22N2O4
271.1805	271.1652	0.0153	56.3	1.26	1	c2 +CO -NH3 [2|3][1-5][4|5][1-2]	C13H22N2O4
271.1805	271.1652	0.0153	56.3	1.26	1	b2 +CO [2|3][1-5][4|5][1-2]	C13H22N2O4
271.1805	271.1329	0.0476	175.7	1.26	1	z2 -NH3 [1|2][1-3][1|2][2-3]	C17H18O3
271.1805	271.1329	0.0476	175.7	1.26	1	z2 -NH3 [3|4][1-6][1|2][5-6]	C17H18O3
277.1375	277.1911	-0.0536	-193.2	0.44	1	a2 [1|2][1-3][2|3][1-2]	C16H24N2O2
277.1375	277.1911	-0.0536	-193.2	0.44	1	a2 [3|4][1-6][5|6][1-2]	C16H24N2O2
277.1375	277.1911	-0.0536	-193.2	0.44	1	c2 +CO -H2O -NH3 [3|4][1-2]	C16H24N2O2
277.1375	277.1911	-0.0536	-193.2	0.44	1	b2 +CO -H2O [3|4][1-2]	C16H24N2O2
277.1375	277.1547	-0.0172	-61.9	0.44	1	b2 [1|2][1-2]	C15H20N2O3
277.1375	277.1547	-0.0172	-61.9	0.44	1	c2 -NH3 [1|2][1-2]	C15H20N2O3
277.1375	277.1183	0.0192	69.3	0.44	1	b2 +CO [7|1][1-2]	C14H16N2O4
277.1375	277.1183	0.0192	69.3	0.44	1	c2 +CO -NH3 [7|1][1-2]	C14H16N2O4
277.1375	277.0972	0.0403	145.6	0.44	1	x1 -H2O -H2O [6|7][7-7]	C17H12N2O2
300.1569	300.2282	-0.0713	-237.4	0.07	1	c2 [6|7][1-6][5|6][1-2]	C15H29N3O3
300.1569	300.2282	-0.0713	-237.4	0.07	1	c2 [4|5][1-3][2|3][1-2]	C15H29N3O3
300.1569	300.1594	-0.0025	-8.4	0.07	1	z2 [1|2][1-4][1|2][3-4]	C18H21NO3

Meas. m/z	Calc. m/z	δ (Da)	δ (ppm)	Rel. Int. (%)	z	Annotation	Formula
300.1569	300.1594	-0.0025	-8.4	0.07	1	z2 [4|5][1-5][1|2][4-5]	C18H21NO3
300.1569	300.1594	-0.0025	-8.4	0.07	1	b2 -NH3 [1|2][1-4][3|4][1-2]	C18H21NO3
300.1569	300.1594	-0.0025	-8.4	0.07	1	b2 -NH3 [4|5][1-5][4|5][1-2]	C18H21NO3
301.1448	301.2122	-0.0674	-223.7	0.11	1	b2 +H2O [6|7][1-6][5|6][1-2]	C15H28N2O4
301.1448	301.2122	-0.0674	-223.7	0.11	1	b2 +H2O [4|5][1-3][2|3][1-2]	C15H28N2O4
301.1448	301.1911	-0.0463	-153.6	0.11	1	z3 -H2O -H2O [4|5][1-4][1|2][2-4]	C18H24N2O2
301.1448	301.1911	-0.0463	-153.6	0.11	1	z3 -H2O -H2O [6|7][1-6][2|3][4-6]	C18H24N2O2
311.1792	311.1965	-0.0173	-55.7	0.12	1	c2 +CO -NH3 [4|5][1-3][2|3][1-2]	C16H26N2O4
311.1792	311.1965	-0.0173	-55.7	0.12	1	b2 +CO [4|5][1-3][2|3][1-2]	C16H26N2O4
311.1792	311.1965	-0.0173	-55.7	0.12	1	b2 +CO [6|7][1-6][5|6][1-2]	C16H26N2O4
311.1792	311.1965	-0.0173	-55.7	0.12	1	c2 +CO -NH3 [6|7][1-6][5|6][1-2]	C16H26N2O4
311.1792	311.1390	0.0402	129.1	0.12	1	x2 -H2O -H2O [6|7][1-3][1|2][2-3]	C18H18N2O3
311.1792	311.1390	0.0402	129.1	0.12	1	x2 -H2O -H2O [1|2][1-6][1|2][5-6]	C18H18N2O3
316.1496	316.2231	-0.0735	-232.4	1.21	1	c2 +CO [6|7][1-5][4|5][1-2]	C15H29N3O4
316.1496	316.2231	-0.0735	-232.4	1.21	1	b3 +H2O [7|1][1-4][2|3][1-3]	C15H29N3O4
316.1496	316.2231	-0.0735	-232.4	1.21	1	b3 +H2O [2|3][1-6][5|6][1-3]	C15H29N3O4
316.1496	316.2231	-0.0735	-232.4	1.21	1	c2 +CO [3|4][1-4][3|4][1-2]	C15H29N3O4
316.1496	316.2020	-0.0524	-165.6	1.21	1	c2 -H2O [1|2][1-4][3|4][1-2]	C18H25N3O2
316.1496	316.2020	-0.0524	-165.6	1.21	1	c2 -H2O [4|5][1-5][4|5][1-2]	C18H25N3O2
316.1496	316.1543	-0.0047	-15.0	1.21	1	b2 +CO -NH3 [3|4][1-6][5|6][1-2]	C18H21NO4
316.1496	316.1543	-0.0047	-15.0	1.21	1	b2 +CO -NH3 [1|2][1-3][2|3][1-2]	C18H21NO4
340.1366	340.2020	-0.0654	-192.1	0.30	1	a2 -H2O [2|3][1-4][3|4][1-2]	C20H25N3O2
340.1366	340.2020	-0.0654	-192.1	0.30	1	b3 -H2O -H2O [7|1][1-4][3|4][1-3]	C20H25N3O2
340.1366	340.2020	-0.0654	-192.1	0.30	1	b3 -H2O -H2O [3|4][1-6][4|5][1-3]	C20H25N3O2
340.1366	340.2020	-0.0654	-192.1	0.30	1	a2 -H2O [5|6][1-5][4|5][1-2]	C20H25N3O2
340.1366	340.1867	-0.0501	-147.3	0.30	1	x3 [6|7][1-4][2|3][2-4]	C16H25N3O5
340.1366	340.1867	-0.0501	-147.3	0.30	1	x3 [2|3][1-6][1|2][4-6]	C16H25N3O5
340.1366	340.1656	-0.0290	-85.2	0.30	1	c2 -H2O -NH3 [7|1][1-6][5|6][1-2]	C19H21N3O3
340.1366	340.1656	-0.0290	-85.2	0.30	1	c2 -H2O -NH3 [5|6][1-3][2|3][1-2]	C19H21N3O3
340.1366	340.1656	-0.0290	-85.2	0.30	1	b2 -H2O [5|6][1-3][2|3][1-2]	C19H21N3O3
340.1366	340.1656	-0.0290	-85.2	0.30	1	b2 -H2O [7|1][1-6][5|6][1-2]	C19H21N3O3
340.1366	340.1543	-0.0177	-52.1	0.30	1	z3 -H2O -NH3 [2|3][5-7]	C20H21NO4
342.1581	342.2387	-0.0806	-235.6	1.49	1	b3 [3|4][1-5][3|4][1-3]	C17H31N3O4
342.1581	342.2387	-0.0806	-235.6	1.49	1	c3 -NH3 [3|4][1-5][3|4][1-3]	C17H31N3O4
342.1581	342.2387	-0.0806	-235.6	1.49	1	b3 [6|7][1-5][4|5][1-3]	C17H31N3O4
342.1581	342.2387	-0.0806	-235.6	1.49	1	c3 -NH3 [6|7][1-5][4|5][1-3]	C17H31N3O4
342.1581	342.2176	-0.0595	-173.9	1.49	1	a3 -H2O [7|1][1-5][4|5][1-3]	C20H27N3O2
342.1581	342.2176	-0.0595	-173.9	1.49	1	a3 -H2O [4|5][1-5][3|4][1-3]	C20H27N3O2
342.1581	342.2023	-0.0442	-129.3	1.49	1	c3 +CO -NH3 [6|7][1-4][3|4][1-3]	C16H27N3O5
342.1581	342.2023	-0.0442	-129.3	1.49	1	b3 +CO [6|7][1-4][3|4][1-3]	C16H27N3O5
342.1581	342.2023	-0.0442	-129.3	1.49	1	b3 +CO [2|3][1-6][4|5][1-3]	C16H27N3O5
342.1581	342.2023	-0.0442	-129.3	1.49	1	c3 +CO -NH3 [2|3][1-6][4|5][1-3]	C16H27N3O5
342.1581	342.1700	-0.0119	-34.7	1.49	1	z3 -NH3 [7|1][1-4][2|3][2-4]	C20H23NO4
342.1581	342.1700	-0.0119	-34.7	1.49	1	z3 -NH3 [3|4][1-6][1|2][4-6]	C20H23NO4
344.2011	344.1969	0.0042	12.3	0.27	1	x3 -H2O -H2O [6|7][1-6][2|3][4-6]	C19H25N3O3
344.2011	344.1969	0.0042	12.3	0.27	1	c2 +CO -H2O [4|5][1-5][4|5][1-2]	C19H25N3O3
344.2011	344.1969	0.0042	12.3	0.27	1	c2 +CO -H2O [1|2][1-4][3|4][1-2]	C19H25N3O3
344.2011	344.1969	0.0042	12.3	0.27	1	x3 -H2O -H2O [4|5][1-4][1|2][2-4]	C19H25N3O3
360.1660	360.2493	-0.0833	-231.2	0.69	1	b3 +H2O [3|4][1-5][3|4][1-3]	C17H33N3O5
360.1660	360.2493	-0.0833	-231.2	0.69	1	b3 +H2O [6|7][1-5][4|5][1-3]	C17H33N3O5
360.1660	360.2282	-0.0622	-172.6	0.69	1	a3 [7|1][1-5][4|5][1-3]	C20H29N3O3
360.1660	360.2282	-0.0622	-172.6	0.69	1	x3 -H2O -H2O [6|7][1-5][1|2][3-5]	C20H29N3O3
360.1660	360.2282	-0.0622	-172.6	0.69	1	x3 -H2O -H2O [2|3][1-5][2|3][3-5]	C20H29N3O3
360.1660	360.2282	-0.0622	-172.6	0.69	1	a3 [4|5][1-5][3|4][1-3]	C20H29N3O3

Meas. m/z	Calc. m/z	δ (Da)	δ (ppm)	Rel. Int. (%)	z	Annotation	Formula
365.1549	365.2547	-0.0998	-273.3	0.45	1	c3 +CO -H2O [3|4][1-5][4|5][1-3]	C19H32N4O3
365.1549	365.2547	-0.0998	-273.3	0.45	1	c3 +CO -H2O [7|1][1-5][3|4][1-3]	C19H32N4O3
365.1549	365.2183	-0.0634	-173.7	0.45	1	c3 [7|1][1-3]	C18H28N4O4
365.1549	365.2071	-0.0522	-142.9	0.45	1	b3 +CO -NH3 [6|7][1-6][5|6][1-3]	C19H28N2O5
365.1549	365.2071	-0.0522	-142.9	0.45	1	b3 +CO -NH3 [4|5][1-4][2|3][1-3]	C19H28N2O5
372.2034	372.2493	-0.0459	-123.3	2.20	1	b3 +H2O [4|5][1-4][2|3][1-3]	C18H33N3O5
372.2034	372.2493	-0.0459	-123.3	2.20	1	b3 +H2O [6|7][1-6][5|6][1-3]	C18H33N3O5
374.1671	374.2438	-0.0767	-205.0	0.75	1	x3 -H2O [5|6][5-7]	C21H31N3O3
374.1671	374.2438	-0.0767	-205.0	0.75	1	a3 -H2O [6|7][1-4][2|3][1-3]	C21H31N3O3
374.1671	374.2438	-0.0767	-205.0	0.75	1	a3 -H2O [1|2][1-6][5|6][1-3]	C21H31N3O3
374.1671	374.2074	-0.0403	-107.8	0.75	1	b3 -H2O [6|7][1-3]	C20H27N3O4
374.1671	374.2074	-0.0403	-107.8	0.75	1	c3 -H2O -NH3 [6|7][1-3]	C20H27N3O4
374.1671	374.1751	-0.0080	-21.3	0.75	1	z2 -H2O -NH3 [6|7][6-7]	C24H23NO3
374.1671	374.1710	-0.0039	-10.5	0.75	1	x3 [3|4][5-7]	C19H23N3O5
391.1704	391.2380	-0.0676	-172.8	1.41	1	z3 -H2O -H2O [3|4][1-6][2|3][4-6]	C25H30N2O2
391.1704	391.2380	-0.0676	-172.8	1.41	1	z3 -H2O -H2O [1|2][1-4][1|2][2-4]	C25H30N2O2
391.1704	391.2340	-0.0636	-162.5	1.41	1	c3 -H2O [6|7][1-3]	C20H30N4O4
391.1704	391.2016	-0.0312	-79.8	1.41	1	b2 -H2O -NH3 [4|5][1-2]	C24H26N2O3
391.1704	391.2016	-0.0312	-79.8	1.41	1	z2 -H2O [6|7][6-7]	C24H26N2O3
392.2032	392.2908	-0.0876	-223.2	1.33	1	a4 -NH3 [2|3][1-6][5|6][1-4]	C22H37N3O3
392.2032	392.2908	-0.0876	-223.2	1.33	1	b3 -H2O [3|4][1-4][3|4][1-3]	C22H37N3O3
392.2032	392.2908	-0.0876	-223.2	1.33	1	c3 -H2O -NH3 [3|4][1-4][3|4][1-3]	C22H37N3O3
392.2032	392.2908	-0.0876	-223.2	1.33	1	c3 -H2O -NH3 [6|7][1-6][4|5][1-3]	C22H37N3O3
392.2032	392.2908	-0.0876	-223.2	1.33	1	b3 -H2O [6|7][1-6][4|5][1-3]	C22H37N3O3
392.2032	392.2908	-0.0876	-223.2	1.33	1	a4 -NH3 [7|1][1-5][2|3][1-4]	C22H37N3O3
392.2032	392.2544	-0.0512	-130.5	1.33	1	x3 [5|6][5-7]	C21H33N3O4
392.2032	392.2544	-0.0512	-130.5	1.33	1	a3 [1|2][1-6][5|6][1-3]	C21H33N3O4
392.2032	392.2544	-0.0512	-130.5	1.33	1	a3 [6|7][1-4][2|3][1-3]	C21H33N3O4
392.2032	392.2220	-0.0188	-48.0	1.33	1	z3 -H2O -NH3 [1|2][1-4][1|2][2-4]	C25H29NO3
392.2032	392.2220	-0.0188	-48.0	1.33	1	z3 -H2O -NH3 [3|4][1-6][2|3][4-6]	C25H29NO3
392.2032	392.2180	-0.0148	-37.7	1.33	1	b3 [6|7][1-3]	C20H29N3O5
392.2032	392.2180	-0.0148	-37.7	1.33	1	c3 -NH3 [6|7][1-3]	C20H29N3O5
392.2032	392.1856	0.0176	44.8	1.33	1	z2 -NH3 [6|7][6-7]	C24H25NO4
404.2203	404.2544	-0.0341	-84.3	7.84	1	b3 [1|2][1-3]	C22H33N3O4
404.2203	404.2544	-0.0341	-84.3	7.84	1	c3 -NH3 [1|2][1-3]	C22H33N3O4
404.2203	404.2180	0.0023	5.7	7.84	1	c3 +CO -NH3 [3|4][1-6][4|5][1-3]	C21H29N3O5
404.2203	404.2180	0.0023	5.7	7.84	1	b2 +H2O [5|6][1-5][4|5][1-2]	C21H29N3O5
404.2203	404.2180	0.0023	5.7	7.84	1	b2 +H2O [2|3][1-4][3|4][1-2]	C21H29N3O5
404.2203	404.2180	0.0023	5.7	7.84	1	b3 +CO [3|4][1-6][4|5][1-3]	C21H29N3O5
404.2203	404.2180	0.0023	5.7	7.84	1	c3 +CO -NH3 [7|1][1-4][3|4][1-3]	C21H29N3O5
404.2203	404.2180	0.0023	5.7	7.84	1	b3 +CO [7|1][1-4][3|4][1-3]	C21H29N3O5
404.2203	404.1969	0.0234	58.0	7.84	1	x2 -H2O -H2O [3|4][1-3][1|2][2-3]	C24H25N3O3
404.2203	404.1969	0.0234	58.0	7.84	1	x2 -H2O -H2O [5|6][1-6][1|2][5-6]	C24H25N3O3
412.1888	412.2595	-0.0707	-171.4	10.85	1	a4 -H2O -NH3 [7|1][1-4]	C24H33N3O3
412.1888	412.2231	-0.0343	-83.2	10.85	1	b3 +CO -H2O -H2O [1|2][1-6][5|6][1-3]	C23H29N3O4
412.1888	412.2231	-0.0343	-83.2	10.85	1	b3 +CO -H2O -H2O [6|7][1-4][2|3][1-3]	C23H29N3O4
412.1888	412.2231	-0.0343	-83.2	10.85	1	c2 -H2O -NH3 [5|6][1-2]	C23H29N3O4
412.1888	412.2231	-0.0343	-83.2	10.85	1	b2 -H2O [5|6][1-2]	C23H29N3O4
412.1888	412.2231	-0.0343	-83.2	10.85	1	x3 -H2O [4|5][5-7]	C23H29N3O4
412.1888	412.1867	0.0021	5.1	10.85	1	x2 [5|6][1-5][1|2][4-5]	C22H25N3O5
412.1888	412.1867	0.0021	5.1	10.85	1	x2 [2|3][1-4][1|2][3-4]	C22H25N3O5
412.1888	412.1543	0.0345	83.6	10.85	1	z2 -H2O -NH3 [1|2][1-5][1|2][4-5]	C26H21NO4
412.1888	412.1543	0.0345	83.6	10.85	1	z2 -H2O -NH3 [5|6][1-4][1|2][3-4]	C26H21NO4
413.2166	413.3122	-0.0956	-231.4	0.68	1	a4 [6|7][1-5][3|4][1-4]	C21H40N4O4

Meas. m/z	Calc. m/z	δ (Da)	δ (ppm)	Rel. Int. (%)	z	Annotation	Formula
413.2166	413.3122	-0.0956	-231.4	0.68	1	a4 [2|3][1-6][4|5][1-4]	C21H40N4O4
413.2166	413.2547	-0.0381	-92.2	0.68	1	c3 +CO -H2O -H2O [1|2][1-3]	C23H32N4O3
413.2166	413.2547	-0.0381	-92.2	0.68	1	c2 -H2O [3|4][1-3][2|3][1-2]	C23H32N4O3
413.2166	413.2547	-0.0381	-92.2	0.68	1	c2 -H2O [5|6][1-6][5|6][1-2]	C23H32N4O3
413.2166	413.2183	-0.0017	-4.2	0.68	1	c2 +CO -H2O [5|6][1-5][4|5][1-2]	C22H28N4O4
413.2166	413.2183	-0.0017	-4.2	0.68	1	c2 +CO -H2O [2|3][1-4][3|4][1-2]	C22H28N4O4
413.2166	413.2071	0.0095	23.0	0.68	1	b3 +CO -H2O -NH3 [6|7][1-4][2|3][1-3]	C23H28N2O5
413.2166	413.2071	0.0095	23.0	0.68	1	b2 -NH3 [5|6][1-2]	C23H28N2O5
413.2166	413.2071	0.0095	23.0	0.68	1	x3 -NH3 [4|5][5-7]	C23H28N2O5
413.2166	413.2071	0.0095	23.0	0.68	1	z2 [7|1][6-7]	C23H28N2O5
413.2166	413.2071	0.0095	23.0	0.68	1	b3 +CO -H2O -NH3 [1|2][1-6][5|6][1-3]	C23H28N2O5
441.2097	441.3071	-0.0974	-220.8	5.88	1	c4 -NH3 [2|3][1-6][4|5][1-4]	C22H40N4O5
441.2097	441.3071	-0.0974	-220.8	5.88	1	c4 -NH3 [6|7][1-5][3|4][1-4]	C22H40N4O5
441.2097	441.3071	-0.0974	-220.8	5.88	1	b4 [6|7][1-5][3|4][1-4]	C22H40N4O5
441.2097	441.3071	-0.0974	-220.8	5.88	1	b4 [2|3][1-6][4|5][1-4]	C22H40N4O5
441.2097	441.2860	-0.0763	-172.9	5.88	1	a4 -H2O [4|5][1-6][3|4][1-4]	C25H36N4O3
441.2097	441.2860	-0.0763	-172.9	5.88	1	a4 -H2O [7|1][1-5][4|5][1-4]	C25H36N4O3
441.2097	441.2496	-0.0399	-90.5	5.88	1	c2 +CO -H2O [3|4][1-3][2|3][1-2]	C24H32N4O4
441.2097	441.2496	-0.0399	-90.5	5.88	1	c2 +CO -H2O [5|6][1-6][5|6][1-2]	C24H32N4O4
441.2097	441.2384	-0.0287	-65.0	5.88	1	z4 -NH3 [4|5][4-7]	C25H32N2O5
441.2097	441.2020	0.0077	17.4	5.88	1	b2 +CO -NH3 [5|6][1-2]	C24H28N2O6
443.2270	443.3017	-0.0747	-168.4	9.01	1	c3 -H2O [1|2][1-4][2|3][1-3]	C25H38N4O3
443.2270	443.3017	-0.0747	-168.4	9.01	1	c3 -H2O [3|4][1-6][5|6][1-3]	C25H38N4O3
443.2270	443.2653	-0.0383	-86.4	9.01	1	c3 +CO -H2O [4|5][1-6][4|5][1-3]	C24H34N4O4
443.2270	443.2653	-0.0383	-86.4	9.01	1	c2 [4|5][1-2]	C24H34N4O4
443.2270	443.2653	-0.0383	-86.4	9.01	1	c3 +CO -H2O [1|2][1-4][3|4][1-3]	C24H34N4O4
445.2429	445.3173	-0.0744	-167.1	0.66	1	b4 -H2O -H2O [6|7][1-6][4|5][1-4]	C25H40N4O3
445.2429	445.3173	-0.0744	-167.1	0.66	1	b4 -H2O -H2O [3|4][1-5][3|4][1-4]	C25H40N4O3
445.2429	445.2809	-0.0380	-85.4	0.66	1	a4 -H2O [6|7][1-4]	C24H36N4O4
445.2429	445.2809	-0.0380	-85.4	0.66	1	x4 -H2O [2|3][1-6][3|4][3-6]	C24H36N4O4
445.2429	445.2809	-0.0380	-85.4	0.66	1	x4 -H2O [7|1][1-5][1|2][2-5]	C24H36N4O4
445.2429	445.2697	-0.0268	-60.2	0.66	1	b3 +H2O -NH3 [3|4][1-6][5|6][1-3]	C25H36N2O5
445.2429	445.2697	-0.0268	-60.2	0.66	1	b3 +H2O -NH3 [1|2][1-4][2|3][1-3]	C25H36N2O5
445.2429	445.2234	0.0195	43.8	0.66	1	c2 -H2O -H2O [1|2][1-5][4|5][1-2]	C26H28N4O3
445.2429	445.2234	0.0195	43.8	0.66	1	c2 -H2O -H2O [5|6][1-4][3|4][1-2]	C26H28N4O3
445.2429	445.2122	0.0307	69.0	0.66	1	z3 -H2O -NH3 [7|1][1-6][1|2][4-6]	C27H28N2O4
445.2429	445.2122	0.0307	69.0	0.66	1	z3 -H2O -NH3 [4|5][1-4][2|3][2-4]	C27H28N2O4
459.2248	459.3177	-0.0929	-202.3	4.45	1	b4 +H2O [2|3][1-6][4|5][1-4]	C22H42N4O6
459.2248	459.3177	-0.0929	-202.3	4.45	1	b4 +H2O [6|7][1-5][3|4][1-4]	C22H42N4O6
459.2248	459.2966	-0.0718	-156.3	4.45	1	a4 [7|1][1-5][4|5][1-4]	C25H38N4O4
459.2248	459.2966	-0.0718	-156.3	4.45	1	a4 [4|5][1-6][3|4][1-4]	C25H38N4O4
459.2248	459.2602	-0.0354	-77.1	4.45	1	c2 +CO [3|4][1-3][2|3][1-2]	C24H34N4O5
459.2248	459.2602	-0.0354	-77.1	4.45	1	c2 +CO [5|6][1-6][5|6][1-2]	C24H34N4O5
473.2350	473.3122	-0.0772	-163.2	5.11	1	a4 -H2O [6|7][1-5][4|5][1-4]	C26H40N4O4
473.2350	473.3122	-0.0772	-163.2	5.11	1	a4 -H2O [3|4][1-6][3|4][1-4]	C26H40N4O4
473.2350	473.3122	-0.0772	-163.2	5.11	1	b4 +CO -H2O -H2O [6|7][1-6][4|5][1-4]	C26H40N4O4
473.2350	473.3122	-0.0772	-163.2	5.11	1	b4 +CO -H2O -H2O [3|4][1-5][3|4][1-4]	C26H40N4O4
473.2350	473.2758	-0.0408	-86.3	5.11	1	a3 [5|6][1-3]	C25H36N4O5
473.2350	473.2758	-0.0408	-86.3	5.11	1	b4 -H2O [6|7][1-4]	C25H36N4O5
473.2350	473.2758	-0.0408	-86.3	5.11	1	c4 -H2O -NH3 [6|7][1-4]	C25H36N4O5
473.2350	473.2435	-0.0085	-17.9	5.11	1	z3 -H2O -NH3 [4|5][1-6][2|3][4-6]	C29H32N2O4
473.2350	473.2435	-0.0085	-17.9	5.11	1	z3 -H2O -NH3 [2|3][1-4][1|2][2-4]	C29H32N2O4
473.2350	473.2183	0.0167	35.2	5.11	1	c2 +CO -H2O -H2O [1|2][1-5][4|5][1-2]	C27H28N4O4
473.2350	473.2183	0.0167	35.2	5.11	1	c2 +CO -H2O -H2O [5|6][1-4][3|4][1-2]	C27H28N4O4

Meas. m/z	Calc. m/z	δ (Da)	δ (ppm)	Rel. Int. (%)	z	Annotation	Formula
473.2350	473.1707	0.0643	135.9	5.11	1	x2 -NH3 [1|2][1-5][1|2][4-5]	C27H24N2O6
473.2350	473.1707	0.0643	135.9	5.11	1	x2 -NH3 [5|6][1-4][1|2][3-4]	C27H24N2O6
475.2524	475.3166	-0.0642	-135.2	66.75	1	z4 -NH3 [2|3][1-5][3|4][2-5]	C27H42N2O5
475.2524	475.3166	-0.0642	-135.2	66.75	1	z4 -NH3 [6|7][1-6][1|2][3-6]	C27H42N2O5
475.2524	475.2915	-0.0391	-82.3	66.75	1	b4 [7|1][1-4]	C25H38N4O5
475.2524	475.2915	-0.0391	-82.3	66.75	1	c4 -NH3 [7|1][1-4]	C25H38N4O5
475.2524	475.2551	-0.0027	-5.7	66.75	1	c2 +CO [5|6][1-2]	C24H34N4O6
475.2524	475.1864	0.0660	139.0	66.75	1	b2 +CO -NH3 [5|6][1-4][3|4][1-2]	C27H26N2O6
475.2524	475.1864	0.0660	139.0	66.75	1	b2 +CO -NH3 [1|2][1-5][4|5][1-2]	C27H26N2O6
491.2513	491.3228	-0.0715	-145.5	10.28	1	b4 +CO -H2O [3|4][1-5][3|4][1-4]	C26H42N4O5
491.2513	491.3228	-0.0715	-145.5	10.28	1	c4 +CO -H2O -NH3 [3|4][1-5][3|4][1-4]	C26H42N4O5
491.2513	491.3228	-0.0715	-145.5	10.28	1	b4 +CO -H2O [6|7][1-6][4|5][1-4]	C26H42N4O5
491.2513	491.3228	-0.0715	-145.5	10.28	1	c4 +CO -H2O -NH3 [6|7][1-6][4|5][1-4]	C26H42N4O5
491.2513	491.3228	-0.0715	-145.5	10.28	1	a4 [3|4][1-6][3|4][1-4]	C26H42N4O5
491.2513	491.3228	-0.0715	-145.5	10.28	1	a4 [6|7][1-5][4|5][1-4]	C26H42N4O5
491.2513	491.2904	-0.0391	-79.7	10.28	1	z4 -H2O -NH3 [5|6][4-7]	C30H38N2O4
491.2513	491.2864	-0.0351	-71.5	10.28	1	b4 [6|7][1-4]	C25H38N4O6
491.2513	491.2864	-0.0351	-71.5	10.28	1	c4 -NH3 [6|7][1-4]	C25H38N4O6
491.2513	491.2540	-0.0027	-5.6	10.28	1	z3 -NH3 [2|3][1-4][1|2][2-4]	C29H34N2O5
491.2513	491.2540	-0.0027	-5.6	10.28	1	z3 -NH3 [4|5][1-6][2|3][4-6]	C29H34N2O5
491.2513	491.2289	0.0224	45.6	10.28	1	c2 +CO -H2O [1|2][1-5][4|5][1-2]	C27H30N4O5
491.2513	491.2289	0.0224	45.6	10.28	1	c2 +CO -H2O [5|6][1-4][3|4][1-2]	C27H30N4O5
491.2513	491.2289	0.0224	45.6	10.28	1	x3 -H2O -H2O [1|2][5-7]	C27H30N4O5
519.3141	519.3541	-0.0400	-77.0	1.35	1	c4 +CO -H2O -NH3 [6|7][1-6][3|4][1-4]	C28H46N4O5
519.3141	519.3541	-0.0400	-77.0	1.35	1	b4 +CO -H2O [2|3][1-5][4|5][1-4]	C28H46N4O5
519.3141	519.3541	-0.0400	-77.0	1.35	1	c4 +CO -H2O -NH3 [2|3][1-5][4|5][1-4]	C28H46N4O5
519.3141	519.3541	-0.0400	-77.0	1.35	1	b4 +CO -H2O [6|7][1-6][3|4][1-4]	C28H46N4O5
519.3141	519.3541	-0.0400	-77.0	1.35	1	a4 [1|2][1-6][5|6][1-4]	C28H46N4O5
519.3141	519.3541	-0.0400	-77.0	1.35	1	a4 [6|7][1-5][2|3][1-4]	C28H46N4O5
519.3141	519.3177	-0.0036	-7.0	1.35	1	c4 -NH3 [3|4][1-6][3|4][1-4]	C27H42N4O6
519.3141	519.3177	-0.0036	-7.0	1.35	1	c4 -NH3 [6|7][1-5][4|5][1-4]	C27H42N4O6
519.3141	519.3177	-0.0036	-7.0	1.35	1	b4 [6|7][1-5][4|5][1-4]	C27H42N4O6
519.3141	519.3177	-0.0036	-7.0	1.35	1	b4 [3|4][1-6][3|4][1-4]	C27H42N4O6
519.3141	519.2853	0.0288	55.4	1.35	1	z3 -NH3 [6|7][5-7]	C31H38N2O5
519.3141	519.2813	0.0328	63.1	1.35	1	c4 +CO -NH3 [6|7][1-4]	C26H38N4O7
519.3141	519.2813	0.0328	63.1	1.35	1	b4 +CO [6|7][1-4]	C26H38N4O7
519.3141	519.2813	0.0328	63.1	1.35	1	b3 +H2O [5|6][1-3]	C26H38N4O7
519.3141	519.2602	0.0539	103.8	1.35	1	x3 -H2O -H2O [2|3][1-5][1|2][3-5]	C29H34N4O5
519.3141	519.2602	0.0539	103.8	1.35	1	x3 -H2O -H2O [5|6][1-5][2|3][3-5]	C29H34N4O5
535.2914	535.3854	-0.0940	-175.6	13.70	1	a5 -NH3 [2|3][1-6][4|5][1-5]	C29H50N4O5
535.2914	535.3854	-0.0940	-175.6	13.70	1	a5 -NH3 [6|7][1-6][3|4][1-5]	C29H50N4O5
535.2914	535.3490	-0.0576	-107.6	13.70	1	x4 [2|3][1-5][3|4][2-5]	C28H46N4O6
535.2914	535.3490	-0.0576	-107.6	13.70	1	x4 [6|7][1-6][1|2][3-6]	C28H46N4O6
535.2914	535.3279	-0.0365	-68.1	13.70	1	b3 -H2O [3|4][1-3]	C31H42N4O4
535.2914	535.3279	-0.0365	-68.1	13.70	1	c3 -H2O -NH3 [3|4][1-3]	C31H42N4O4
535.2914	535.3279	-0.0365	-68.1	13.70	1	b4 +CO -H2O -H2O [1|2][1-4]	C31H42N4O4
535.2914	535.2915	-0.0001	-0.2	13.70	1	a3 [5|6][1-5][3|4][1-3]	C30H38N4O5
535.2914	535.2915	-0.0001	-0.2	13.70	1	a3 [1|2][1-5][4|5][1-3]	C30H38N4O5
535.2914	535.2915	-0.0001	-0.2	13.70	1	b3 +CO -H2O [4|5][1-6][5|6][1-3]	C30H38N4O5
535.2914	535.2915	-0.0001	-0.2	13.70	1	c3 +CO -H2O -NH3 [4|5][1-6][5|6][1-3]	C30H38N4O5
535.2914	535.2915	-0.0001	-0.2	13.70	1	b3 +CO -H2O [2|3][1-4][2|3][1-3]	C30H38N4O5
535.2914	535.2915	-0.0001	-0.2	13.70	1	c3 +CO -H2O -NH3 [2|3][1-4][2|3][1-3]	C30H38N4O5
535.2914	535.2803	0.0111	20.8	13.70	1	z3 -NH3 [7|1][5-7]	C31H38N2O6
535.2914	535.2551	0.0363	67.8	13.70	1	b3 [7|1][1-6][5|6][1-3]	C29H34N4O6

Meas. m/z	Calc. m/z	δ (Da)	δ (ppm)	Rel. Int. (%)	z	Annotation	Formula
535.2914	535.2551	0.0363	67.8	13.70	1	b3 [5|6][1-4][2|3][1-3]	C29H34N4O6
535.2914	535.2551	0.0363	67.8	13.70	1	c3 -NH3 [7|1][1-6][5|6][1-3]	C29H34N4O6
535.2914	535.2551	0.0363	67.8	13.70	1	c3 -NH3 [5|6][1-4][2|3][1-3]	C29H34N4O6
543.3369	543.3541	-0.0172	-31.6	0.26	1	c4 -NH3 [1|2][1-4]	C30H46N4O5
543.3369	543.3541	-0.0172	-31.6	0.26	1	b4 [1|2][1-4]	C30H46N4O5
543.3369	543.3177	0.0192	35.3	0.26	1	b3 +H2O [2|3][1-4][2|3][1-3]	C29H42N4O6
543.3369	543.3177	0.0192	35.3	0.26	1	c4 +CO -NH3 [7|1][1-5][3|4][1-4]	C29H42N4O6
543.3369	543.3177	0.0192	35.3	0.26	1	c4 +CO -NH3 [3|4][1-6][4|5][1-4]	C29H42N4O6
543.3369	543.3177	0.0192	35.3	0.26	1	b4 +CO [3|4][1-6][4|5][1-4]	C29H42N4O6
543.3369	543.3177	0.0192	35.3	0.26	1	b4 +CO [7|1][1-5][3|4][1-4]	C29H42N4O6
543.3369	543.3177	0.0192	35.3	0.26	1	b3 +H2O [4|5][1-6][5|6][1-3]	C29H42N4O6
543.3369	543.2966	0.0403	74.2	0.26	1	a3 -H2O -H2O [5|6][1-4][3|4][1-3]	C32H38N4O4
543.3369	543.2966	0.0403	74.2	0.26	1	a3 -H2O -H2O [1|2][1-6][4|5][1-3]	C32H38N4O4
543.3369	543.2966	0.0403	74.2	0.26	1	x3 -H2O -H2O [6|7][5-7]	C32H38N4O4
558.3762	558.3286	0.0476	85.2	0.16	1	c3 +CO [5|6][1-6][4|5][1-3]	C29H43N5O6
558.3762	558.3286	0.0476	85.2	0.16	1	c4 +CO -H2O [6|7][1-6][5|6][1-4]	C29H43N5O6
558.3762	558.3286	0.0476	85.2	0.16	1	c4 +CO -H2O [4|5][1-5][2|3][1-4]	C29H43N5O6
558.3762	558.3286	0.0476	85.2	0.16	1	c3 +CO [2|3][1-4][3|4][1-3]	C29H43N5O6
558.3762	558.3174	0.0588	105.4	0.16	1	b4 +CO -NH3 [1|2][1-6][5|6][1-4]	C30H43N3O7
558.3762	558.3174	0.0588	105.4	0.16	1	b3 +H2O -NH3 [3|4][1-4][2|3][1-3]	C30H43N3O7
558.3762	558.3174	0.0588	105.4	0.16	1	b3 +H2O -NH3 [5|6][1-6][5|6][1-3]	C30H43N3O7
558.3762	558.3174	0.0588	105.4	0.16	1	b4 +CO -NH3 [6|7][1-5][2|3][1-4]	C30H43N3O7
558.3762	558.2962	0.0800	143.2	0.16	1	a3 -NH3 [1|2][1-5][3|4][1-3]	C33H39N3O5
558.3762	558.2962	0.0800	143.2	0.16	1	a3 -NH3 [4|5][1-5][4|5][1-3]	C33H39N3O5
582.3175	582.3650	-0.0475	-81.6	2.77	1	a4 -H2O [3|4][1-5][2|3][1-4]	C32H47N5O5
582.3175	582.3650	-0.0475	-81.6	2.77	1	a4 -H2O [5|6][1-6][5|6][1-4]	C32H47N5O5
582.3175	582.3650	-0.0475	-81.6	2.77	1	b5 -H2O -H2O [6|7][1-5]	C32H47N5O5
582.3175	582.3286	-0.0111	-19.1	2.77	1	c4 -H2O -NH3 [5|6][1-5][4|5][1-4]	C31H43N5O6
582.3175	582.3286	-0.0111	-19.1	2.77	1	b4 -H2O [5|6][1-5][4|5][1-4]	C31H43N5O6
582.3175	582.3286	-0.0111	-19.1	2.77	1	c4 -H2O -NH3 [2|3][1-6][3|4][1-4]	C31H43N5O6
582.3175	582.3286	-0.0111	-19.1	2.77	1	b4 -H2O [2|3][1-6][3|4][1-4]	C31H43N5O6
582.3175	582.2599	0.0576	99.0	2.77	1	z4 -H2O -NH3 [5|6][1-5][1|2][2-5]	C34H35N3O6
582.3175	582.2599	0.0576	99.0	2.77	1	z4 -H2O -NH3 [7|1][1-6][3|4][3-6]	C34H35N3O6
586.3221	586.3963	-0.0742	-126.5	9.26	1	a5 [7|1][1-5]	C32H51N5O5
586.3221	586.3599	-0.0378	-64.5	9.26	1	c3 [4|5][1-3]	C31H47N5O6
586.3221	586.2912	0.0309	52.8	9.26	1	b3 -NH3 [4|5][1-5][4|5][1-3]	C34H39N3O6
586.3221	586.2912	0.0309	52.8	9.26	1	z3 [4|5][1-5][2|3][3-5]	C34H39N3O6
586.3221	586.2912	0.0309	52.8	9.26	1	z3 [1|2][1-5][1|2][3-5]	C34H39N3O6
586.3221	586.2912	0.0309	52.8	9.26	1	b3 -NH3 [1|2][1-5][3|4][1-3]	C34H39N3O6
590.3541	590.3912	-0.0371	-62.9	1.77	1	a5 [6|7][1-5]	C31H51N5O6
590.3541	590.3912	-0.0371	-62.9	1.77	1	b5 +CO -H2O [2|3][1-6][4|5][1-5]	C31H51N5O6
590.3541	590.3912	-0.0371	-62.9	1.77	1	c5 +CO -H2O -NH3 [2|3][1-6][4|5][1-5]	C31H51N5O6
590.3541	590.3912	-0.0371	-62.9	1.77	1	c5 +CO -H2O -NH3 [6|7][1-6][3|4][1-5]	C31H51N5O6
590.3541	590.3912	-0.0371	-62.9	1.77	1	b5 +CO -H2O [6|7][1-6][3|4][1-5]	C31H51N5O6
590.3541	590.3225	0.0316	53.6	1.77	1	z4 -NH3 [7|1][1-6][1|2][3-6]	C34H43N3O6
590.3541	590.3225	0.0316	53.6	1.77	1	z4 -NH3 [3|4][1-5][3|4][2-5]	C34H43N3O6
590.3541	590.2973	0.0568	96.2	1.77	1	c3 +CO -H2O [5|6][1-5][3|4][1-3]	C32H39N5O6
590.3541	590.2973	0.0568	96.2	1.77	1	c3 +CO -H2O [1|2][1-5][4|5][1-3]	C32H39N5O6
590.3541	590.2973	0.0568	96.2	1.77	1	x4 -H2O -H2O [2|3][1-6][1|2][3-6]	C32H39N5O6
590.3541	590.2973	0.0568	96.2	1.77	1	x4 -H2O -H2O [5|6][1-5][3|4][2-5]	C32H39N5O6
590.3541	590.2861	0.0680	115.2	1.77	1	b3 -NH3 [1|2][1-6][4|5][1-3]	C33H39N3O7
590.3541	590.2861	0.0680	115.2	1.77	1	z3 [1|2][1-6][1|2][4-6]	C33H39N3O7
590.3541	590.2861	0.0680	115.2	1.77	1	z3 [5|6][1-4][2|3][2-4]	C33H39N3O7
590.3541	590.2861	0.0680	115.2	1.77	1	b3 -NH3 [5|6][1-4][3|4][1-3]	C33H39N3O7

Meas. m/z	Calc. m/z	δ (Da)	δ (ppm)	Rel. Int. (%)	z	Annotation	Formula
614.3453	614.3912	-0.0459	-74.7	2.51	1	b5 [7|1][1-5]	C33H51N5O6
614.3453	614.3912	-0.0459	-74.7	2.51	1	c5 -NH3 [7|1][1-5]	C33H51N5O6
614.3453	614.3548	-0.0095	-15.5	2.51	1	c3 +CO [4|5][1-3]	C32H47N5O7
614.3453	614.3337	0.0116	18.9	2.51	1	a4 -H2O -H2O [5|6][1-4]	C35H43N5O5
614.3453	614.3337	0.0116	18.9	2.51	1	x4 -H2O -H2O [7|1][1-6][1|2][3-6]	C35H43N5O5
614.3453	614.3337	0.0116	18.9	2.51	1	x4 -H2O -H2O [3|4][1-5][3|4][2-5]	C35H43N5O5
614.3453	614.2861	0.0592	96.4	2.51	1	b3 +CO -NH3 [4|5][1-5][4|5][1-3]	C35H39N3O7
614.3453	614.2861	0.0592	96.4	2.51	1	b3 +CO -NH3 [1|2][1-5][3|4][1-3]	C35H39N3O7
615.2949	615.3752	-0.0803	-130.5	0.94	1	b5 +H2O -NH3 [7|1][1-5]	C33H50N4O7
615.2949	615.3541	-0.0592	-96.2	0.94	1	z4 -H2O -H2O [2|3][1-5][1|2][2-5]	C36H46N4O5
615.2949	615.3541	-0.0592	-96.2	0.94	1	z4 -H2O -H2O [4|5][1-6][3|4][3-6]	C36H46N4O5
615.2949	615.3289	-0.0340	-55.3	0.94	1	c4 -H2O -H2O [7|1][1-6][5|6][1-4]	C34H42N6O5
615.2949	615.3289	-0.0340	-55.3	0.94	1	c4 -H2O -H2O [5|6][1-5][2|3][1-4]	C34H42N6O5
615.2949	615.3177	-0.0228	-37.1	0.94	1	a4 -H2O -NH3 [5|6][1-4]	C35H42N4O6
615.2949	615.3177	-0.0228	-37.1	0.94	1	x4 -H2O -NH3 [7|1][1-6][1|2][3-6]	C35H42N4O6
615.2949	615.3177	-0.0228	-37.1	0.94	1	x4 -H2O -NH3 [3|4][1-5][3|4][2-5]	C35H42N4O6
615.2949	615.2813	0.0136	22.1	0.94	1	x3 -H2O [1|2][1-6][1|2][4-6]	C34H38N4O7
615.2949	615.2813	0.0136	22.1	0.94	1	x3 -H2O [5|6][1-4][2|3][2-4]	C34H38N4O7
618.3370	618.3861	-0.0491	-79.4	100.00	1	b5 [6|7][1-5]	C32H51N5O7
618.3370	618.3861	-0.0491	-79.4	100.00	1	c5 -NH3 [6|7][1-5]	C32H51N5O7
618.3370	618.3538	-0.0168	-27.1	100.00	1	z4 -NH3 [6|7][4-7]	C36H47N3O6
618.3370	618.3497	-0.0127	-20.6	100.00	1	b4 +H2O [2|3][1-6][3|4][1-4]	C31H47N5O8
618.3370	618.3497	-0.0127	-20.6	100.00	1	b4 +H2O [5|6][1-5][4|5][1-4]	C31H47N5O8
618.3370	618.3286	0.0084	13.6	100.00	1	x4 -H2O -H2O [5|6][1-6][3|4][3-6]	C34H43N5O6
618.3370	618.3286	0.0084	13.6	100.00	1	x4 -H2O -H2O [3|4][1-5][1|2][2-5]	C34H43N5O6
618.3370	618.2810	0.0560	90.6	100.00	1	b3 +CO -NH3 [1|2][1-6][4|5][1-3]	C34H39N3O8
618.3370	618.2810	0.0560	90.6	100.00	1	b3 +CO -NH3 [5|6][1-4][3|4][1-3]	C34H39N3O8
634.3503	634.3963	-0.0460	-72.5	1.39	1	c4 -H2O -NH3 [2|3][1-4]	C36H51N5O5
634.3503	634.3963	-0.0460	-72.5	1.39	1	b4 -H2O [2|3][1-4]	C36H51N5O5
634.3503	634.3851	-0.0348	-54.8	1.39	1	z5 -H2O -NH3 [6|7][1-6][1|2][2-6]	C37H51N3O6
634.3503	634.3851	-0.0348	-54.8	1.39	1	z5 -H2O -NH3 [1|2][1-6][4|5][2-6]	C37H51N3O6
634.3503	634.3599	-0.0096	-15.2	1.39	1	c4 +CO -H2O -NH3 [7|1][1-6][3|4][1-4]	C35H47N5O6
634.3503	634.3599	-0.0096	-15.2	1.39	1	c4 +CO -H2O -NH3 [3|4][1-5][4|5][1-4]	C35H47N5O6
634.3503	634.3599	-0.0096	-15.2	1.39	1	b4 +CO -H2O [3|4][1-5][4|5][1-4]	C35H47N5O6
634.3503	634.3599	-0.0096	-15.2	1.39	1	b4 +CO -H2O [7|1][1-6][3|4][1-4]	C35H47N5O6
634.3503	634.3487	0.0016	2.6	1.39	1	z4 -NH3 [4|5][1-6][3|4][3-6]	C36H47N3O7
634.3503	634.3487	0.0016	2.6	1.39	1	z4 -NH3 [2|3][1-5][1|2][2-5]	C36H47N3O7
634.3503	634.3235	0.0268	42.2	1.39	1	b4 [5|6][1-5][2|3][1-4]	C34H43N5O7
634.3503	634.3235	0.0268	42.2	1.39	1	b4 [7|1][1-6][5|6][1-4]	C34H43N5O7
634.3503	634.3235	0.0268	42.2	1.39	1	c3 +CO -H2O [5|6][1-4][3|4][1-3]	C34H43N5O7
634.3503	634.3235	0.0268	42.2	1.39	1	c3 +CO -H2O [1|2][1-6][4|5][1-3]	C34H43N5O7
634.3503	634.3235	0.0268	42.2	1.39	1	c4 -NH3 [5|6][1-5][2|3][1-4]	C34H43N5O7
634.3503	634.3235	0.0268	42.2	1.39	1	c4 -NH3 [7|1][1-6][5|6][1-4]	C34H43N5O7
644.3157	644.3806	-0.0649	-100.8	0.24	1	a4 -H2O [1|2][1-5][4|5][1-4]	C37H49N5O5
644.3157	644.3806	-0.0649	-100.8	0.24	1	b4 +CO -H2O -H2O [2|3][1-4]	C37H49N5O5
644.3157	644.3806	-0.0649	-100.8	0.24	1	a4 -H2O [5|6][1-6][3|4][1-4]	C37H49N5O5
644.3157	644.3694	-0.0537	-83.4	0.24	1	z4 -H2O -NH3 [7|1][4-7]	C38H49N3O6
644.3157	644.3654	-0.0497	-77.1	0.24	1	x5 [4|5][3-7]	C33H49N5O8
644.3157	644.3079	0.0078	12.1	0.24	1	c4 +CO -H2O -NH3 [7|1][1-6][5|6][1-4]	C35H41N5O7
644.3157	644.3079	0.0078	12.1	0.24	1	b4 +CO -H2O [5|6][1-5][2|3][1-4]	C35H41N5O7
644.3157	644.3079	0.0078	12.1	0.24	1	c4 +CO -H2O -NH3 [5|6][1-5][2|3][1-4]	C35H41N5O7
644.3157	644.3079	0.0078	12.1	0.24	1	b4 +CO -H2O [7|1][1-6][5|6][1-4]	C35H41N5O7
644.3157	644.2966	0.0191	29.6	0.24	1	z4 -NH3 [2|3][4-7]	C36H41N3O8
660.3004	660.3756	-0.0752	-113.8	0.22	1	b4 +CO -H2O -H2O [2|3][1-5][2|3][1-4]	C37H49N5O6

Meas. m/z	Calc. m/z	δ (Da)	δ (ppm)	Rel. Int. (%)	z	Annotation	Formula
660.3004	660.3756	-0.0752	-113.8	0.22	1	x4 -H2O [6|7][4-7]	C37H49N5O6
660.3004	660.3756	-0.0752	-113.8	0.22	1	b4 +CO -H2O -H2O [4|5][1-6][5|6][1-4]	C37H49N5O6
660.3004	660.3756	-0.0752	-113.8	0.22	1	a4 -H2O [1|2][1-6][4|5][1-4]	C37H49N5O6
660.3004	660.3756	-0.0752	-113.8	0.22	1	a4 -H2O [5|6][1-5][3|4][1-4]	C37H49N5O6
660.3004	660.3392	-0.0388	-58.7	0.22	1	c4 -H2O -NH3 [5|6][1-4]	C36H45N5O7
660.3004	660.3392	-0.0388	-58.7	0.22	1	b4 -H2O [5|6][1-4]	C36H45N5O7
660.3004	660.3028	-0.0024	-3.6	0.22	1	x4 [7|1][1-6][3|4][3-6]	C35H41N5O8
660.3004	660.3028	-0.0024	-3.6	0.22	1	x4 [5|6][1-5][1|2][2-5]	C35H41N5O8
672.3347	672.3756	-0.0409	-60.8	0.08	1	b4 -H2O [1|2][1-5][4|5][1-4]	C38H49N5O6
672.3347	672.3756	-0.0409	-60.8	0.08	1	b4 -H2O [5|6][1-6][3|4][1-4]	C38H49N5O6
672.3347	672.3756	-0.0409	-60.8	0.08	1	c4 -H2O -NH3 [1|2][1-5][4|5][1-4]	C38H49N5O6
672.3347	672.3756	-0.0409	-60.8	0.08	1	c4 -H2O -NH3 [5|6][1-6][3|4][1-4]	C38H49N5O6
672.3347	672.3279	0.0068	10.1	0.08	1	z4 -NH3 [5|6][1-5][2|3][2-5]	C38H45N3O8
672.3347	672.3279	0.0068	10.1	0.08	1	z4 -NH3 [1|2][1-6][2|3][3-6]	C38H45N3O8
678.3547	678.4225	-0.0678	-100.0	1.97	1	b4 -H2O [3|4][1-4]	C38H55N5O6
678.3547	678.4225	-0.0678	-100.0	1.97	1	c4 -H2O -NH3 [3|4][1-4]	C38H55N5O6
678.3547	678.4225	-0.0678	-100.0	1.97	1	a5 -NH3 [7|1][1-6][2|3][1-5]	C38H55N5O6
678.3547	678.4225	-0.0678	-100.0	1.97	1	a5 -NH3 [2|3][1-6][5|6][1-5]	C38H55N5O6
678.3547	678.4225	-0.0678	-100.0	1.97	1	b5 +CO -H2O -H2O [1|2][1-6][5|6][1-5]	C38H55N5O6
678.3547	678.4225	-0.0678	-100.0	1.97	1	b5 +CO -H2O -H2O [6|7][1-6][2|3][1-5]	C38H55N5O6
678.3547	678.3861	-0.0314	-46.3	1.97	1	x4 [6|7][4-7]	C37H51N5O7
678.3547	678.3861	-0.0314	-46.3	1.97	1	a4 [1|2][1-6][4|5][1-4]	C37H51N5O7
678.3547	678.3861	-0.0314	-46.3	1.97	1	a4 [5|6][1-5][3|4][1-4]	C37H51N5O7
678.3547	678.3861	-0.0314	-46.3	1.97	1	b4 +CO -H2O [4|5][1-6][5|6][1-4]	C37H51N5O7
678.3547	678.3861	-0.0314	-46.3	1.97	1	c4 +CO -H2O -NH3 [2|3][1-5][2|3][1-4]	C37H51N5O7
678.3547	678.3861	-0.0314	-46.3	1.97	1	b4 +CO -H2O [2|3][1-5][2|3][1-4]	C37H51N5O7
678.3547	678.3861	-0.0314	-46.3	1.97	1	c4 +CO -H2O -NH3 [4|5][1-6][5|6][1-4]	C37H51N5O7
678.3547	678.3538	0.0009	1.4	1.97	1	z4 -H2O -NH3 [1|2][1-5][1|2][2-5]	C41H47N3O6
678.3547	678.3538	0.0009	1.4	1.97	1	z4 -H2O -NH3 [3|4][1-6][3|4][3-6]	C41H47N3O6
678.3547	678.3497	0.0050	7.3	1.97	1	b4 [5|6][1-4]	C36H47N5O8
678.3547	678.3497	0.0050	7.3	1.97	1	c4 -NH3 [5|6][1-4]	C36H47N5O8
710.3524	710.4123	-0.0599	-84.4	0.14	1	b5 -NH3 [5|6][1-6][4|5][1-5]	C38H55N5O8
710.3524	710.4123	-0.0599	-84.4	0.14	1	z5 [5|6][1-6][3|4][2-6]	C38H55N5O8
710.3524	710.4123	-0.0599	-84.4	0.14	1	b5 -NH3 [2|3][1-6][3|4][1-5]	C38H55N5O8
710.3524	710.4123	-0.0599	-84.4	0.14	1	z5 [2|3][1-6][2|3][2-6]	C38H55N5O8
710.3524	710.3912	-0.0388	-54.6	0.14	1	a5 -H2O -NH3 [4|5][1-6][3|4][1-5]	C41H51N5O6
710.3524	710.3912	-0.0388	-54.6	0.14	1	b4 -H2O -H2O [1|2][1-6][3|4][1-4]	C41H51N5O6
710.3524	710.3912	-0.0388	-54.6	0.14	1	a5 -H2O -NH3 [7|1][1-6][4|5][1-5]	C41H51N5O6
710.3524	710.3912	-0.0388	-54.6	0.14	1	b4 -H2O -H2O [4|5][1-5][4|5][1-4]	C41H51N5O6
710.3524	710.3548	-0.0024	-3.4	0.14	1	x4 -H2O [4|5][1-6][2|3][3-6]	C40H47N5O7
710.3524	710.3548	-0.0024	-3.4	0.14	1	x4 -H2O [1|2][1-5][2|3][2-5]	C40H47N5O7
711.3384	711.4228	-0.0844	-118.7	0.15	1	c4 -H2O -H2O [1|2][1-5][2|3][1-4]	C41H54N6O5
711.3384	711.4228	-0.0844	-118.7	0.15	1	c4 -H2O -H2O [3|4][1-6][5|6][1-4]	C41H54N6O5
711.3384	711.3865	-0.0481	-67.6	0.15	1	c4 +CO -H2O -H2O [4|5][1-6][4|5][1-4]	C40H50N6O6
711.3384	711.3865	-0.0481	-67.6	0.15	1	c4 +CO -H2O -H2O [1|2][1-5][3|4][1-4]	C40H50N6O6
711.3384	711.3752	-0.0368	-51.8	0.15	1	z4 -H2O [1|2][1-6][1|2][3-6]	C41H50N4O7
711.3384	711.3752	-0.0368	-51.8	0.15	1	z4 -H2O [4|5][1-5][3|4][2-5]	C41H50N4O7
711.3384	711.3752	-0.0368	-51.8	0.15	1	b4 -H2O -NH3 [1|2][1-6][3|4][1-4]	C41H50N4O7
711.3384	711.3752	-0.0368	-51.8	0.15	1	b4 -H2O -NH3 [4|5][1-5][4|5][1-4]	C41H50N4O7
711.3384	711.3388	-0.0004	-0.6	0.15	1	x4 -NH3 [1|2][1-5][2|3][2-5]	C40H46N4O8
711.3384	711.3388	-0.0004	-0.6	0.15	1	x4 -NH3 [4|5][1-6][2|3][3-6]	C40H46N4O8
725.4340	725.4021	0.0319	44.0	0.25	1	b5 -H2O -H2O [5|6][1-6][2|3][1-5]	C41H52N6O6
725.4340	725.4021	0.0319	44.0	0.25	1	b5 -H2O -H2O [7|1][1-6][5|6][1-5]	C41H52N6O6
725.4340	725.3545	0.0795	109.6	0.25	1	z5 -H2O -NH3 [3|4][3-7]	C41H48N4O8

Meas. m/z	Calc. m/z	δ (Da)	δ (ppm)	Rel. Int. (%)	z	Annotation	Formula
727.3339	727.4178	-0.0839	-115.3	0.18	1	c4 -H2O -H2O [1|2][1-6][3|4][1-4]	C41H54N6O6
727.3339	727.4178	-0.0839	-115.3	0.18	1	a5 -H2O [7|1][1-6][4|5][1-5]	C41H54N6O6
727.3339	727.4178	-0.0839	-115.3	0.18	1	c4 -H2O -H2O [4|5][1-5][4|5][1-4]	C41H54N6O6
727.3339	727.4178	-0.0839	-115.3	0.18	1	a5 -H2O [4|5][1-6][3|4][1-5]	C41H54N6O6
727.3339	727.3701	-0.0362	-49.8	0.18	1	z5 -NH3 [7|1][1-6][4|5][2-6]	C41H50N4O8
727.3339	727.3701	-0.0362	-49.8	0.18	1	z5 -NH3 [5|6][1-6][1|2][2-6]	C41H50N4O8
743.3357	743.4127	-0.0770	-103.5	0.94	1	b5 -H2O [7|1][1-6][5|6][1-5]	C41H54N6O7
743.3357	743.4127	-0.0770	-103.5	0.94	1	c5 -H2O -NH3 [7|1][1-6][5|6][1-5]	C41H54N6O7
743.3357	743.4127	-0.0770	-103.5	0.94	1	b5 -H2O [5|6][1-6][2|3][1-5]	C41H54N6O7
743.3357	743.4127	-0.0770	-103.5	0.94	1	c5 -H2O -NH3 [5|6][1-6][2|3][1-5]	C41H54N6O7
743.3357	743.3651	-0.0294	-39.5	0.94	1	z5 -NH3 [3|4][3-7]	C41H50N4O9
749.4336	749.4960	-0.0624	-83.3	0.32	1	a5 -H2O [2|3][1-5]	C42H64N6O6
749.4336	749.4596	-0.0260	-34.7	0.32	1	b5 -H2O [3|4][1-5]	C41H60N6O7
749.4336	749.4596	-0.0260	-34.7	0.32	1	c5 -H2O -NH3 [3|4][1-5]	C41H60N6O7
749.4336	749.4596	-0.0260	-34.7	0.32	1	b6 +CO -H2O -H2O [6|7][1-6]	C41H60N6O7
749.4336	749.4232	0.0104	13.8	0.32	1	b5 +CO -H2O [4|5][1-6][5|6][1-5]	C40H56N6O8
749.4336	749.4232	0.0104	13.8	0.32	1	c5 +CO -H2O -NH3 [2|3][1-6][2|3][1-5]	C40H56N6O8
749.4336	749.4232	0.0104	13.8	0.32	1	x5 [2|3][1-6][4|5][2-6]	C40H56N6O8
749.4336	749.4232	0.0104	13.8	0.32	1	a5 [5|6][1-5]	C40H56N6O8
749.4336	749.4232	0.0104	13.8	0.32	1	x5 [7|1][1-6][1|2][2-6]	C40H56N6O8
749.4336	749.4232	0.0104	13.8	0.32	1	c5 +CO -H2O -NH3 [4|5][1-6][5|6][1-5]	C40H56N6O8
749.4336	749.4232	0.0104	13.8	0.32	1	b5 +CO -H2O [2|3][1-6][2|3][1-5]	C40H56N6O8
749.4336	749.3909	0.0427	57.0	0.32	1	z5 -H2O -NH3 [3|4][1-6][3|4][2-6]	C44H52N4O7
749.4336	749.3909	0.0427	57.0	0.32	1	z5 -H2O -NH3 [7|1][1-6][2|3][2-6]	C44H52N4O7
757.4178	757.4858	-0.0680	-89.8	5.55	1	b6 [6|7][1-6]	C40H64N6O8
757.4178	757.4858	-0.0680	-89.8	5.55	1	c6 -NH3 [6|7][1-6]	C40H64N6O8
757.4178	757.4495	-0.0317	-41.8	5.55	1	b5 +H2O [4|5][1-6][5|6][1-5]	C39H60N6O9
757.4178	757.4495	-0.0317	-41.8	5.55	1	b5 +H2O [2|3][1-6][2|3][1-5]	C39H60N6O9
757.4178	757.4283	-0.0105	-13.9	5.55	1	x5 -H2O -H2O [1|2][3-7]	C42H56N6O7
757.4178	757.4283	-0.0105	-13.9	5.55	1	c4 +CO -H2O [1|2][1-5][2|3][1-4]	C42H56N6O7
757.4178	757.4283	-0.0105	-13.9	5.55	1	c4 +CO -H2O [3|4][1-6][5|6][1-4]	C42H56N6O7
757.4178	757.3807	0.0371	49.0	5.55	1	b4 +CO -NH3 [1|2][1-6][3|4][1-4]	C42H52N4O9
757.4178	757.3807	0.0371	49.0	5.55	1	b4 +CO -NH3 [4|5][1-5][4|5][1-4]	C42H52N4O9
757.5160	757.4858	0.0302	39.8	0.82	1	c6 -NH3 [6|7][1-6]	C40H64N6O8
757.5160	757.4858	0.0302	39.8	0.82	1	b6 [6|7][1-6]	C40H64N6O8
757.5160	757.4495	0.0665	87.9	0.82	1	b5 +H2O [2|3][1-6][2|3][1-5]	C39H60N6O9
757.5160	757.4495	0.0665	87.9	0.82	1	b5 +H2O [4|5][1-6][5|6][1-5]	C39H60N6O9
757.5160	757.4283	0.0877	115.8	0.82	1	x5 -H2O -H2O [1|2][3-7]	C42H56N6O7
757.5160	757.4283	0.0877	115.8	0.82	1	c4 +CO -H2O [1|2][1-5][2|3][1-4]	C42H56N6O7
757.5160	757.4283	0.0877	115.8	0.82	1	c4 +CO -H2O [3|4][1-6][5|6][1-4]	C42H56N6O7
759.3435	759.4076	-0.0641	-84.4	0.38	1	c5 -H2O -NH3 [5|6][1-5]	C41H54N6O8
759.3435	759.4076	-0.0641	-84.4	0.38	1	b5 -H2O [5|6][1-5]	C41H54N6O8
777.3919	777.4909	-0.0990	-127.4	0.40	1	c5 -H2O -NH3 [2|3][1-5]	C43H64N6O7
777.3919	777.4909	-0.0990	-127.4	0.40	1	b5 -H2O [2|3][1-5]	C43H64N6O7
777.3919	777.4545	-0.0626	-80.6	0.40	1	a5 [3|4][1-6][2|3][1-5]	C42H60N6O8
777.3919	777.4545	-0.0626	-80.6	0.40	1	c5 +CO -H2O -NH3 [3|4][1-5]	C42H60N6O8
777.3919	777.4545	-0.0626	-80.6	0.40	1	b5 +CO -H2O [3|4][1-5]	C42H60N6O8
777.3919	777.4545	-0.0626	-80.6	0.40	1	a5 [5|6][1-6][5|6][1-5]	C42H60N6O8
777.3919	777.4222	-0.0303	-38.9	0.40	1	z5 -H2O -NH3 [6|7][3-7]	C46H56N4O7
777.3919	777.4182	-0.0263	-33.8	0.40	1	b5 [5|6][1-5]	C41H56N6O9
777.3919	777.4182	-0.0263	-33.8	0.40	1	c5 -NH3 [5|6][1-5]	C41H56N6O9
782.4379	782.4600	-0.0221	-28.2	0.25	1	c5 -H2O -H2O [7|1][1-6][3|4][1-5]	C44H59N7O6
782.4379	782.4600	-0.0221	-28.2	0.25	1	c5 -H2O -H2O [3|4][1-6][4|5][1-5]	C44H59N7O6
782.4379	782.4487	-0.0108	-13.8	0.25	1	a5 -H2O -NH3 [1|2][1-6][3|4][1-5]	C45H59N5O7

Meas. m/z	Calc. m/z	δ (Da)	δ (ppm)	Rel. Int. (%)	z	Annotation	Formula
782.4379	782.4487	-0.0108	-13.8	0.25	1	a5 -H2O -NH3 [4|5][1-6][4|5][1-5]	C45H59N5O7
782.4379	782.4236	0.0143	18.3	0.25	1	c5 +CO -H2O -H2O [7|1][1-6][4|5][1-5]	C43H55N7O7
782.4379	782.4236	0.0143	18.3	0.25	1	c5 +CO -H2O -H2O [4|5][1-6][3|4][1-5]	C43H55N7O7
782.4379	782.4123	0.0256	32.7	0.25	1	z5 -H2O [2|3][3-7]	C44H55N5O8
782.4379	782.4123	0.0256	32.7	0.25	1	b5 -H2O -NH3 [4|5][1-5]	C44H55N5O8
782.4379	782.3760	0.0619	79.2	0.25	1	x5 -NH3 [7|1][1-6][3|4][2-6]	C43H51N5O9
782.4379	782.3760	0.0619	79.2	0.25	1	x5 -NH3 [4|5][1-6][2|3][2-6]	C43H51N5O9
796.4203	796.4855	-0.0652	-81.9	0.21	1	b5 +H2O -NH3 [2|3][1-5]	C43H65N5O9
796.4203	796.3916	0.0287	36.0	0.21	1	x5 -H2O -NH3 [3|4][1-6][1|2][2-6]	C44H53N5O9
796.4203	796.3916	0.0287	36.0	0.21	1	x5 -H2O -NH3 [5|6][1-6][4|5][2-6]	C44H53N5O9
811.3926	811.4753	-0.0827	-101.9	1.11	1	c5 -H2O -NH3 [1|2][1-5]	C46H62N6O7
811.3926	811.4753	-0.0827	-101.9	1.11	1	b5 -H2O [1|2][1-5]	C46H62N6O7
811.3926	811.4389	-0.0463	-57.0	1.11	1	c5 +CO -H2O -NH3 [7|1][1-6][3|4][1-5]	C45H58N6O8
811.3926	811.4389	-0.0463	-57.0	1.11	1	b5 +CO -H2O [3|4][1-6][4|5][1-5]	C45H58N6O8
811.3926	811.4389	-0.0463	-57.0	1.11	1	b5 +CO -H2O [7|1][1-6][3|4][1-5]	C45H58N6O8
811.3926	811.4389	-0.0463	-57.0	1.11	1	c5 +CO -H2O -NH3 [3|4][1-6][4|5][1-5]	C45H58N6O8
811.3926	811.4277	-0.0351	-43.2	1.11	1	z5 -NH3 [1|2][1-6][2|3][2-6]	C46H58N4O9
811.3926	811.4277	-0.0351	-43.2	1.11	1	z5 -NH3 [4|5][1-6][3|4][2-6]	C46H58N4O9
811.5396	811.4753	0.0643	79.3	0.09	1	c5 -H2O -NH3 [1|2][1-5]	C46H62N6O7
811.5396	811.4753	0.0643	79.3	0.09	1	b5 -H2O [1|2][1-5]	C46H62N6O7
814.4139	814.4862	-0.0723	-88.7	1.08	1	c5 -H2O -H2O [1|2][1-6][4|5][1-5]	C45H63N7O7
814.4139	814.4862	-0.0723	-88.7	1.08	1	c5 -H2O -H2O [5|6][1-6][3|4][1-5]	C45H63N7O7
814.4139	814.4749	-0.0610	-74.9	1.08	1	z6 -H2O -NH3 [1|2][2-7]	C46H63N5O8
814.4139	814.4498	-0.0359	-44.1	1.08	1	c5 +CO -H2O -H2O [3|4][1-6][2|3][1-5]	C44H59N7O8
814.4139	814.4498	-0.0359	-44.1	1.08	1	c5 +CO -H2O -H2O [5|6][1-6][5|6][1-5]	C44H59N7O8
814.4139	814.4022	0.0117	14.4	1.08	1	x5 -NH3 [3|4][1-6][1|2][2-6]	C44H55N5O10
814.4139	814.4022	0.0117	14.4	1.08	1	x5 -NH3 [5|6][1-6][4|5][2-6]	C44H55N5O10
814.5666	814.4862	0.0804	98.7	0.17	1	c5 -H2O -H2O [5|6][1-6][3|4][1-5]	C45H63N7O7
814.5666	814.4862	0.0804	98.7	0.17	1	c5 -H2O -H2O [1|2][1-6][4|5][1-5]	C45H63N7O7
814.5666	814.4749	0.0917	112.5	0.17	1	z6 -H2O -NH3 [1|2][2-7]	C46H63N5O8
850.4156	850.5073	-0.0917	-107.8	7.03	1	c5 [1|2][1-6][4|5][1-5]	C45H67N7O9
850.4156	850.5073	-0.0917	-107.8	7.03	1	c5 [5|6][1-6][3|4][1-5]	C45H67N7O9
850.4156	850.4709	-0.0553	-65.0	7.03	1	c5 +CO [3|4][1-6][2|3][1-5]	C44H63N7O10
850.4156	850.4709	-0.0553	-65.0	7.03	1	c5 +CO [5|6][1-6][5|6][1-5]	C44H63N7O10
850.5634	850.5073	0.0561	66.0	0.56	1	c5 [1|2][1-6][4|5][1-5]	C45H67N7O9
850.5634	850.5073	0.0561	66.0	0.56	1	c5 [5|6][1-6][3|4][1-5]	C45H67N7O9
850.5634	850.4709	0.0925	108.7	0.56	1	c5 +CO [3|4][1-6][2|3][1-5]	C44H63N7O10
850.5634	850.4709	0.0925	108.7	0.56	1	c5 +CO [5|6][1-6][5|6][1-5]	C44H63N7O10
882.4355	882.5124	-0.0769	-87.1	15.53	1	c6 -H2O -NH3 [7|1][1-6]	C49H67N7O8
882.4355	882.5124	-0.0769	-87.1	15.53	1	b6 -H2O [7|1][1-6]	C49H67N7O8
882.4355	882.5124	-0.0769	-87.1	15.53	1	c5 +CO -H2O -H2O [3|4][1-6][5|6][1-5]	C49H67N7O8
882.4355	882.5124	-0.0769	-87.1	15.53	1	c5 +CO -H2O -H2O [1|2][1-6][2|3][1-5]	C49H67N7O8
882.4355	882.4648	-0.0293	-33.2	15.53	1	z6 -NH3 [3|4][2-7]	C49H63N5O10
882.4355	882.4648	-0.0293	-33.2	15.53	1	x5 -NH3 [1|2][1-6][1|2][2-6]	C49H63N5O10
882.4355	882.4648	-0.0293	-33.2	15.53	1	x5 -NH3 [3|4][1-6][4|5][2-6]	C49H63N5O10
882.6028	882.5124	0.0904	102.4	0.86	1	b6 -H2O [7|1][1-6]	C49H67N7O8
882.6028	882.5124	0.0904	102.4	0.86	1	c6 -H2O -NH3 [7|1][1-6]	C49H67N7O8
882.6028	882.5124	0.0904	102.4	0.86	1	c5 +CO -H2O -H2O [1|2][1-6][2|3][1-5]	C49H67N7O8
882.6028	882.5124	0.0904	102.4	0.86	1	c5 +CO -H2O -H2O [3|4][1-6][5|6][1-5]	C49H67N7O8
886.4272	886.5073	-0.0801	-90.4	1.30	1	b6 -H2O [5|6][1-6]	C48H67N7O9
886.4272	886.5073	-0.0801	-90.4	1.30	1	c6 -H2O -NH3 [5|6][1-6]	C48H67N7O9
886.5474	886.5073	0.0401	45.2	0.21	1	b6 -H2O [5|6][1-6]	C48H67N7O9
886.5474	886.5073	0.0401	45.2	0.21	1	c6 -H2O -NH3 [5|6][1-6]	C48H67N7O9
914.4369	914.5022	-0.0653	-71.4	0.08	1	b6 +CO -H2O [5|6][1-6]	C49H67N7O10

Meas. m/z	Calc. m/z	δ (Da)	δ (ppm)	Rel. Int. (%)	z	Annotation	Formula
914.4369	914.5022	-0.0653	-71.4	0.08	1	c6 +CO -H2O -NH3 [5|6][1-6]	C49H67N7O10
926.4511	926.5386	-0.0875	-94.4	0.14	1	b6 -H2O [3|4][1-6]	C51H71N7O9
926.4511	926.5386	-0.0875	-94.4	0.14	1	c6 -H2O -NH3 [3|4][1-6]	C51H71N7O9
926.4511	926.5022	-0.0511	-55.2	0.14	1	x6 [6|7][2-7]	C50H67N7O10
926.4511	926.5022	-0.0511	-55.2	0.14	1	c6 +CO -H2O -NH3 [4|5][1-6]	C50H67N7O10
926.4511	926.5022	-0.0511	-55.2	0.14	1	b6 +CO -H2O [4|5][1-6]	C50H67N7O10
931.4460	931.5288	-0.0828	-88.9	0.03	1	c6 +CO -H2O [5|6][1-6]	C49H70N8O10
980.4521	980.5492	-0.0971	-99.0	0.06	1	x6 -H2O [7|1][2-7]	C54H73N7O10
980.5668	980.5492	0.0176	18.0	0.06	1	x6 -H2O [7|1][2-7]	C54H73N7O10
981.4958	981.5808	-0.0850	-86.6	0.06	1	c6 +CO -H2O -H2O [1|2][1-6]	C54H76N8O9
981.4958	981.5332	-0.0374	-38.1	0.06	1	x6 -NH3 [7|1][2-7]	C54H72N6O11
981.6310	981.5808	0.0502	51.1	0.13	1	c6 +CO -H2O -H2O [1|2][1-6]	C54H76N8O9
981.6310	981.5332	0.0978	99.7	0.13	1	x6 -NH3 [7|1][2-7]	C54H72N6O11

Generated by mMass • Open Source Mass Spectrometry Tool • www.mmass.org

mMass Report: *Seglitide_IT_filtered_ms3*

**Date**	Thu Sep 15 17:29:47 2011	**Scan Number**	
**Operator**		**Retention Time**	
**Contact**	http://bix.ucsd.edu/nrp/	**MS Level**	
**Institution**		**Precursor m/z**	
**Instrument**		**Polarity**	unknown
		**Spectrum Points**	0
		**Peak List**	53

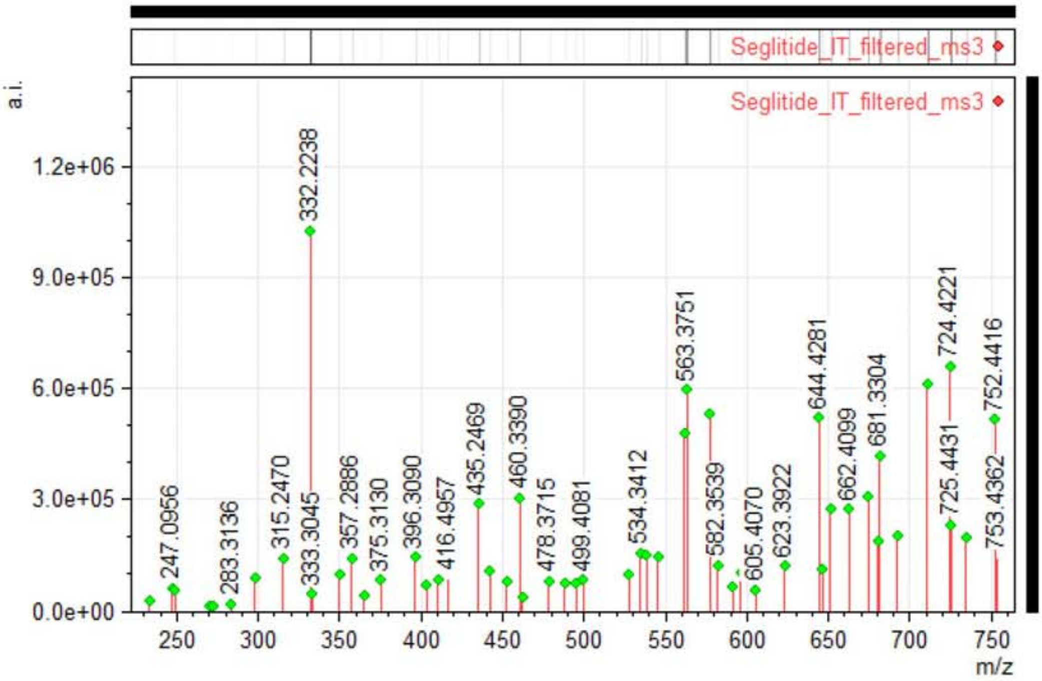

Sequence - *Seglitide*

Accession	Length	Mo. Mass	Av. Mass	Coverage	Matched Int.
	6 (Cyclic)	808.4272	808.9668	100.0 %	97.8 %

NMe-Ala | Tyr | Trp | Lys | Val | Phe

Meas. m/z	Calc. m/z	δ (Da)	δ (ppm)	Rel. Int. (%)	z	Annotation	Formula
233.1176	233.1285	-0.0109	-46.6	2.60	1	c2 -NH3 [5|6][1-2]	C13H16N2O2
233.1176	233.1285	-0.0109	-46.6	2.60	1	b2 [5|6][1-2]	C13H16N2O2
247.0956	247.1441	-0.0485	-196.2	6.11	1	a2 -NH3 [1|2][1-3][2|3][1-2]	C14H18N2O2
247.0956	247.1441	-0.0485	-196.2	6.11	1	a2 -NH3 [3|4][1-5][4|5][1-2]	C14H18N2O2
247.0956	247.1441	-0.0485	-196.2	6.11	1	b2 [4|5][1-2]	C14H18N2O2
247.0956	247.1441	-0.0485	-196.2	6.11	1	c2 -NH3 [4|5][1-2]	C14H18N2O2
249.0830	249.1234	-0.0404	-162.1	5.69	1	c2 -NH3 [6|1][1-2]	C13H16N2O3
249.0830	249.1234	-0.0404	-162.1	5.69	1	b2 [6|1][1-2]	C13H16N2O3
270.2289	270.1601	0.0688	254.8	1.35	1	a2 -NH3 [2|3][1-2]	C16H19N3O
270.2289	270.1125	0.1164	431.1	1.35	1	b2 +CO -NH3 -NH3 [3|4][1-3][2|3][1-2]	C16H15NO3
270.2289	270.1125	0.1164	431.1	1.35	1	b2 +CO -NH3 -NH3 [5|6][1-5][4|5][1-2]	C16H15NO3
272.2496	272.1394	0.1102	405.0	1.21	1	c2 -NH3 [2|3][1-5][4|5][1-2]	C15H17N3O2

Meas. m/z	Calc. m/z	δ (Da)	δ (ppm)	Rel. Int. (%)	z	Annotation	Formula
272.2496	272.1394	0.1102	405.0	1.21	1	b2 [6|1][1-3][2|3][1-2]	C15H17N3O2
272.2496	272.1394	0.1102	405.0	1.21	1	c2 -NH3 [6|1][1-3][2|3][1-2]	C15H17N3O2
272.2496	272.1394	0.1102	405.0	1.21	1	b2 [2|3][1-5][4|5][1-2]	C15H17N3O2
272.2496	272.0917	0.1578	580.1	1.21	1	x2 -NH3 [1|2][1-4][1|2][3-4]	C15H13NO4
272.2496	272.0917	0.1578	580.1	1.21	1	x2 -NH3 [4|5][1-4][1|2][3-4]	C15H13NO4
283.3136	283.1441	0.1695	598.6	1.95	1	a2 [5|6][1-3][2|3][1-2]	C17H18N2O2
283.3136	283.1441	0.1695	598.6	1.95	1	a2 [1|2][1-5][4|5][1-2]	C17H18N2O2
298.2034	298.1914	0.0120	40.4	8.74	1	a3 -H2O -NH3 [5|6][1-5][4|5][1-3]	C18H23N3O
298.2034	298.1914	0.0120	40.4	8.74	1	a3 -H2O -NH3 [3|4][1-4][2|3][1-3]	C18H23N3O
298.2034	298.1550	0.0484	162.4	8.74	1	b2 -NH3 [2|3][1-2]	C17H19N3O2
298.2034	298.1550	0.0484	162.4	8.74	1	c2 -NH3 -NH3 [2|3][1-2]	C17H19N3O2
298.2034	298.1550	0.0484	162.4	8.74	1	z2 [4|5][5-6]	C17H19N3O2
298.2034	298.1438	0.0597	200.1	8.74	1	z3 -NH3 [1|2][4-6]	C18H19NO3
298.2034	298.1186	0.0848	284.5	8.74	1	x2 [2|3][1-5][1|2][4-5]	C16H15N3O3
298.2034	298.1186	0.0848	284.5	8.74	1	x2 [6|1][1-3][1|2][2-3]	C16H15N3O3
298.2034	298.0863	0.1172	393.1	8.74	1	z2 -H2O -NH3 [3|4][5-6]	C20H11NO2
315.2470	315.2179	0.0290	92.1	14.00	1	a3 -H2O [5|6][1-5][4|5][1-3]	C18H26N4O
315.2470	315.2179	0.0290	92.1	14.00	1	a3 -H2O [3|4][1-4][2|3][1-3]	C18H26N4O
315.2470	315.1816	0.0654	207.6	14.00	1	c2 -NH3 [2|3][1-2]	C17H22N4O2
315.2470	315.1816	0.0654	207.6	14.00	1	b2 [2|3][1-2]	C17H22N4O2
315.2470	315.1703	0.0767	243.2	14.00	1	a3 -NH3 -NH3 [6|1][1-4][3|4][1-3]	C18H22N2O3
315.2470	315.1703	0.0767	243.2	14.00	1	b3 -NH3 [4|5][1-3]	C18H22N2O3
315.2470	315.1703	0.0767	243.2	14.00	1	a3 -NH3 -NH3 [3|4][1-5][3|4][1-3]	C18H22N2O3
315.2470	315.1703	0.0767	243.2	14.00	1	z3 [1|2][4-6]	C18H22N2O3
315.2470	315.1128	0.1342	425.8	14.00	1	b2 -H2O -NH3 [1|2][1-2]	C20H14N2O2
315.2470	315.1128	0.1342	425.8	14.00	1	z2 -H2O [3|4][5-6]	C20H14N2O2
332.2238	332.2081	0.0157	47.4	100.00	1	c2 [2|3][1-2]	C17H25N5O2
332.2238	332.1969	0.0270	81.2	100.00	1	a3 -NH3 [6|1][1-4][3|4][1-3]	C18H25N3O3
332.2238	332.1969	0.0270	81.2	100.00	1	c3 -NH3 [4|5][1-3]	C18H25N3O3
332.2238	332.1969	0.0270	81.2	100.00	1	b3 [4|5][1-3]	C18H25N3O3
332.2238	332.1969	0.0270	81.2	100.00	1	a3 -NH3 [3|4][1-5][3|4][1-3]	C18H25N3O3
332.2238	332.1394	0.0845	254.4	100.00	1	c2 -H2O -NH3 [1|2][1-2]	C20H17N3O2
332.2238	332.1394	0.0845	254.4	100.00	1	b2 -H2O [1|2][1-2]	C20H17N3O2
333.3045	333.2285	0.0760	228.2	4.40	1	a3 [5|6][1-5][4|5][1-3]	C18H28N4O2
333.3045	333.2285	0.0760	228.2	4.40	1	a3 [3|4][1-4][2|3][1-3]	C18H28N4O2
333.3045	333.1921	0.1124	337.4	4.40	1	b2 +H2O [2|3][1-2]	C17H24N4O3
333.3045	333.1809	0.1237	371.1	4.40	1	b3 +H2O -NH3 [4|5][1-3]	C18H24N2O4
333.3045	333.1710	0.1335	400.8	4.40	1	c2 -H2O [5|6][1-4][3|4][1-2]	C20H20N4O
333.3045	333.1710	0.1335	400.8	4.40	1	c2 -H2O [2|3][1-4][3|4][1-2]	C20H20N4O
333.3045	333.1598	0.1448	434.6	4.40	1	a3 -H2O -NH3 [5|6][1-3]	C21H20N2O2
333.3045	333.1234	0.1812	543.8	4.40	1	b2 -NH3 [1|2][1-2]	C20H16N2O3
333.3045	333.1234	0.1812	543.8	4.40	1	z2 [3|4][5-6]	C20H16N2O3
350.1610	350.2074	-0.0465	-132.6	9.55	1	b3 +H2O [4|5][1-3]	C18H27N3O4
350.1610	350.1863	-0.0253	-72.3	9.55	1	a3 -H2O [5|6][1-3]	C21H23N3O2
350.1610	350.1499	0.0111	31.6	9.55	1	c2 -NH3 [1|2][1-2]	C20H19N3O3
350.1610	350.1499	0.0111	31.6	9.55	1	b2 [1|2][1-2]	C20H19N3O3
357.2886	357.2285	0.0601	168.3	13.91	1	c3 -H2O -NH3 [3|4][1-3]	C20H28N4O2
357.2886	357.2285	0.0601	168.3	13.91	1	b3 -H2O [3|4][1-3]	C20H28N4O2
357.2886	357.1809	0.1078	301.7	13.91	1	b3 -NH3 -NH3 [3|4][1-5][4|5][1-3]	C20H24N2O4
357.2886	357.1809	0.1078	301.7	13.91	1	b3 -NH3 -NH3 [1|2][1-4][2|3][1-3]	C20H24N2O4
357.2886	357.1809	0.1078	301.7	13.91	1	z3 -NH3 [3|4][1-5][2|3][3-5]	C20H24N2O4
357.2886	357.1809	0.1078	301.7	13.91	1	z3 -NH3 [1|2][1-4][1|2][2-4]	C20H24N2O4
357.2886	357.1445	0.1441	403.6	13.91	1	x3 -NH3 [6|1][1-5][2|3][3-5]	C19H20N2O5
357.2886	357.1445	0.1441	403.6	13.91	1	x3 -NH3 [4|5][1-4][1|2][2-4]	C19H20N2O5

Meas. m/z	Calc. m/z	δ (Da)	δ (ppm)	Rel. Int. (%)	z	Annotation	Formula
365.2102	365.2183	-0.0081	-22.2	4.29	1	c3 [6|1][1-5][4|5][1-3]	C18H28N4O4
365.2102	365.2183	-0.0081	-22.2	4.29	1	c3 [4|5][1-4][2|3][1-3]	C18H28N4O4
365.2102	365.1972	0.0130	35.7	4.29	1	b3 -H2O -NH3 [2|3][1-5][4|5][1-3]	C21H24N4O2
365.2102	365.1972	0.0130	35.7	4.29	1	z3 -H2O [2|3][1-5][2|3][3-5]	C21H24N4O2
365.2102	365.1972	0.0130	35.7	4.29	1	b3 -H2O -NH3 [6|1][1-4][2|3][1-3]	C21H24N4O2
365.2102	365.1972	0.0130	35.7	4.29	1	z3 -H2O [6|1][1-4][1|2][2-4]	C21H24N4O2
365.2102	365.1860	0.0243	66.5	4.29	1	a3 -NH3 [4|5][1-4][3|4][1-3]	C22H24N2O3
365.2102	365.1860	0.0243	66.5	4.29	1	a3 -NH3 [1|2][1-5][3|4][1-3]	C22H24N2O3
375.3130	375.2391	0.0739	196.9	8.13	1	b3 [3|4][1-3]	C20H30N4O3
375.3130	375.2391	0.0739	196.9	8.13	1	c3 -NH3 [3|4][1-3]	C20H30N4O3
375.3130	375.2027	0.1103	293.9	8.13	1	c3 +CO -H2O [6|1][1-5][4|5][1-3]	C19H26N4O4
375.3130	375.2027	0.1103	293.9	8.13	1	c3 +CO -H2O [4|5][1-4][2|3][1-3]	C19H26N4O4
375.3130	375.1914	0.1215	323.9	8.13	1	b3 +H2O -NH3 -NH3 [3|4][1-5][4|5][1-3]	C20H26N2O5
375.3130	375.1914	0.1215	323.9	8.13	1	b3 +H2O -NH3 -NH3 [1|2][1-4][2|3][1-3]	C20H26N2O5
375.3130	375.1703	0.1426	380.2	8.13	1	z3 -H2O [1|2][1-5][1|2][3-5]	C23H22N2O3
375.3130	375.1703	0.1426	380.2	8.13	1	b3 -H2O -NH3 [4|5][1-4][3|4][1-3]	C23H22N2O3
375.3130	375.1703	0.1426	380.2	8.13	1	z3 -H2O [4|5][1-4][2|3][2-4]	C23H22N2O3
375.3130	375.1703	0.1426	380.2	8.13	1	b3 -H2O -NH3 [1|2][1-5][3|4][1-3]	C23H22N2O3
396.3090	396.2394	0.0696	175.5	14.23	1	b3 -H2O [2|3][1-3]	C22H29N5O2
396.3090	396.2394	0.0696	175.5	14.23	1	c3 -H2O -NH3 [2|3][1-3]	C22H29N5O2
396.3090	396.1918	0.1172	295.8	14.23	1	c3 -NH3 [5|6][1-3]	C22H25N3O4
396.3090	396.1918	0.1172	295.8	14.23	1	b3 [5|6][1-3]	C22H25N3O4
403.2673	403.2340	0.0334	82.7	7.08	1	c3 +CO -NH3 [3|4][1-3]	C21H30N4O4
403.2673	403.2340	0.0334	82.7	7.08	1	b3 +CO [3|4][1-3]	C21H30N4O4
403.2673	403.2129	0.0545	135.1	7.08	1	a3 -H2O [1|2][1-4][3|4][1-3]	C24H26N4O2
403.2673	403.2129	0.0545	135.1	7.08	1	a3 -H2O [4|5][1-5][3|4][1-3]	C24H26N4O2
403.2673	403.1976	0.0697	173.0	7.08	1	x3 [6|1][1-4][2|3][2-4]	C20H26N4O5
403.2673	403.1976	0.0697	173.0	7.08	1	x3 [3|4][1-5][1|2][3-5]	C20H26N4O5
403.2673	403.1652	0.1021	253.2	7.08	1	b3 +CO -H2O -NH3 [4|5][1-4][3|4][1-3]	C24H22N2O4
403.2673	403.1652	0.1021	253.2	7.08	1	b3 +CO -H2O -NH3 [1|2][1-5][3|4][1-3]	C24H22N2O4
410.2630	410.2187	0.0443	108.0	8.52	1	c3 +CO -H2O -NH3 [6|1][1-4][2|3][1-3]	C22H27N5O3
410.2630	410.2187	0.0443	108.0	8.52	1	b3 +CO -H2O [6|1][1-4][2|3][1-3]	C22H27N5O3
410.2630	410.2187	0.0443	108.0	8.52	1	b3 +CO -H2O [2|3][1-5][4|5][1-3]	C22H27N5O3
410.2630	410.2187	0.0443	108.0	8.52	1	c3 +CO -H2O -NH3 [2|3][1-5][4|5][1-3]	C22H27N5O3
410.2630	410.2074	0.0555	135.4	8.52	1	c3 -NH3 [4|5][1-4][3|4][1-3]	C23H27N3O4
410.2630	410.2074	0.0555	135.4	8.52	1	c3 -NH3 [1|2][1-5][3|4][1-3]	C23H27N3O4
410.2630	410.2074	0.0555	135.4	8.52	1	b3 [4|5][1-4][3|4][1-3]	C23H27N3O4
410.2630	410.2074	0.0555	135.4	8.52	1	b3 [1|2][1-5][3|4][1-3]	C23H27N3O4
410.2630	410.1863	0.0767	186.9	8.52	1	z3 -H2O -NH3 [2|3][1-4][2|3][2-4]	C26H23N3O2
410.2630	410.1863	0.0767	186.9	8.52	1	z3 -H2O -NH3 [5|6][1-5][1|2][3-5]	C26H23N3O2
410.2630	410.1499	0.1131	275.6	8.52	1	x3 -H2O -NH3 [2|3][1-5][1|2][3-5]	C25H19N3O3
410.2630	410.1499	0.1131	275.6	8.52	1	x3 -H2O -NH3 [5|6][1-4][2|3][2-4]	C25H19N3O3
435.2469	435.2027	0.0443	101.7	28.16	1	b3 [6|1][1-3]	C24H26N4O4
435.2469	435.2027	0.0443	101.7	28.16	1	c3 -NH3 [6|1][1-3]	C24H26N4O4
442.3918	442.2813	0.1106	250.0	10.71	1	c4 -H2O -NH3 [3|4][1-4]	C24H35N5O3
442.3918	442.2813	0.1106	250.0	10.71	1	b4 -H2O [3|4][1-4]	C24H35N5O3
442.3918	442.2449	0.1470	332.3	10.71	1	c3 +CO -NH3 [2|3][1-3]	C23H31N5O4
442.3918	442.2449	0.1470	332.3	10.71	1	b3 +CO [2|3][1-3]	C23H31N5O4
442.3918	442.2336	0.1582	357.7	10.71	1	z4 -NH3 [6|1][1-5][2|3][2-5]	C24H31N3O5
442.3918	442.2336	0.1582	357.7	10.71	1	b4 -NH3 -NH3 [6|1][1-5][3|4][1-4]	C24H31N3O5
442.3918	442.2336	0.1582	357.7	10.71	1	z4 -NH3 [3|4][1-5][2|3][2-5]	C24H31N3O5
442.3918	442.2336	0.1582	357.7	10.71	1	b4 -NH3 -NH3 [3|4][1-5][3|4][1-4]	C24H31N3O5
452.2784	452.2292	0.0492	108.7	7.88	1	c3 [6|1][1-3]	C24H29N5O4
452.2784	452.2180	0.0604	133.5	7.88	1	x4 -NH3 -NH3 [1|2][3-6]	C25H29N3O5

Meas. m/z	Calc. m/z	δ (Da)	δ (ppm)	Rel. Int. (%)	z	Annotation	Formula
452.2784	452.1969	0.0815	180.3	7.88	1	a3 -NH3 [5|6][1-4][2|3][1-3]	C28H25N3O3
452.2784	452.1969	0.0815	180.3	7.88	1	a3 -NH3 [1|2][1-5][4|5][1-3]	C28H25N3O3
460.3390	460.2918	0.0472	102.5	29.73	1	c4 -NH3 [3|4][1-4]	C24H37N5O4
460.3390	460.2918	0.0472	102.5	29.73	1	b4 [3|4][1-4]	C24H37N5O4
460.3390	460.2442	0.0948	205.9	29.73	1	b4 +H2O -NH3 -NH3 [6|1][1-5][3|4][1-4]	C24H33N3O6
460.3390	460.2442	0.0948	205.9	29.73	1	b4 +H2O -NH3 -NH3 [3|4][1-5][3|4][1-4]	C24H33N3O6
460.3390	460.2343	0.1047	227.4	29.73	1	c3 -H2O -NH3 [1|2][1-3]	C26H29N5O3
460.3390	460.2343	0.1047	227.4	29.73	1	c3 +CO -H2O [4|5][1-5][4|5][1-3]	C26H29N5O3
460.3390	460.2343	0.1047	227.4	29.73	1	c3 +CO -H2O [2|3][1-4][2|3][1-3]	C26H29N5O3
460.3390	460.2343	0.1047	227.4	29.73	1	b3 -H2O [1|2][1-3]	C26H29N5O3
460.3390	460.2231	0.1159	251.8	29.73	1	z4 -H2O [2|3][3-6]	C27H29N3O4
460.3390	460.2231	0.1159	251.8	29.73	1	b4 -H2O -NH3 [4|5][1-4]	C27H29N3O4
460.3390	460.1867	0.1523	330.9	29.73	1	b3 +CO -NH3 [1|2][1-4][3|4][1-3]	C26H25N3O5
460.3390	460.1867	0.1523	330.9	29.73	1	b3 +CO -NH3 [4|5][1-5][3|4][1-3]	C26H25N3O5
462.3264	462.2500	0.0764	165.3	3.63	1	c3 -NH3 [5|6][1-5][3|4][1-3]	C26H31N5O3
462.3264	462.2500	0.0764	165.3	3.63	1	c3 -NH3 [2|3][1-4][3|4][1-3]	C26H31N5O3
462.3264	462.2500	0.0764	165.3	3.63	1	b3 [2|3][1-4][3|4][1-3]	C26H31N5O3
462.3264	462.2500	0.0764	165.3	3.63	1	b3 [5|6][1-5][3|4][1-3]	C26H31N5O3
462.3264	462.2387	0.0876	189.6	3.63	1	a4 -NH3 -NH3 [5|6][1-5][4|5][1-4]	C27H31N3O4
462.3264	462.2387	0.0876	189.6	3.63	1	a4 -NH3 -NH3 [3|4][1-5][2|3][1-4]	C27H31N3O4
462.3264	462.2136	0.1128	244.0	3.63	1	c3 +CO -H2O [6|1][1-3]	C25H27N5O4
462.3264	462.2023	0.1240	268.3	3.63	1	b3 +H2O -NH3 -NH3 [1|2][1-3]	C26H27N3O5
462.3264	462.1812	0.1451	314.0	3.63	1	z3 -H2O [5|6][1-4][1|2][2-4]	C29H23N3O3
462.3264	462.1812	0.1451	314.0	3.63	1	b3 -H2O -NH3 [5|6][1-4][2|3][1-3]	C29H23N3O3
462.3264	462.1812	0.1451	314.0	3.63	1	z3 -H2O [1|2][1-5][2|3][3-5]	C29H23N3O3
462.3264	462.1812	0.1451	314.0	3.63	1	b3 -H2O -NH3 [1|2][1-5][4|5][1-3]	C29H23N3O3
478.3715	478.3024	0.0691	144.4	8.00	1	b4 +H2O [3|4][1-4]	C24H39N5O5
478.3715	478.2813	0.0902	188.6	8.00	1	a4 -H2O [3|4][1-5][2|3][1-4]	C27H35N5O3
478.3715	478.2813	0.0902	188.6	8.00	1	a4 -H2O [5|6][1-5][4|5][1-4]	C27H35N5O3
478.3715	478.2449	0.1266	264.7	8.00	1	b3 [1|2][1-3]	C26H31N5O4
478.3715	478.2449	0.1266	264.7	8.00	1	c3 +CO [4|5][1-5][4|5][1-3]	C26H31N5O4
478.3715	478.2449	0.1266	264.7	8.00	1	c3 +CO [2|3][1-4][2|3][1-3]	C26H31N5O4
478.3715	478.2449	0.1266	264.7	8.00	1	c3 -NH3 [1|2][1-3]	C26H31N5O4
478.3715	478.2336	0.1378	288.2	8.00	1	z4 [2|3][3-6]	C27H31N3O5
478.3715	478.2336	0.1378	288.2	8.00	1	b4 -NH3 [4|5][1-4]	C27H31N3O5
488.3333	488.2867	0.0465	95.2	7.34	1	b4 +CO [3|4][1-4]	C25H37N5O5
488.3333	488.2867	0.0465	95.2	7.34	1	c4 +CO -NH3 [3|4][1-4]	C25H37N5O5
488.3333	488.2656	0.0676	138.5	7.34	1	a4 -H2O [6|1][1-5][4|5][1-4]	C28H33N5O3
488.3333	488.2656	0.0676	138.5	7.34	1	a4 -H2O [4|5][1-5][2|3][1-4]	C28H33N5O3
488.3333	488.2292	0.1040	213.1	7.34	1	b3 +CO -H2O [1|2][1-3]	C27H29N5O4
488.3333	488.2292	0.1040	213.1	7.34	1	x3 [5|6][1-5][1|2][3-5]	C27H29N5O4
488.3333	488.2292	0.1040	213.1	7.34	1	x3 [2|3][1-4][2|3][2-4]	C27H29N5O4
488.3333	488.2292	0.1040	213.1	7.34	1	c3 +CO -H2O -NH3 [1|2][1-3]	C27H29N5O4
488.3333	488.2180	0.1153	236.1	7.34	1	b4 +CO -H2O -NH3 [4|5][1-4]	C28H29N3O5
488.3333	488.1605	0.1728	353.9	7.34	1	x3 -H2O -NH3 [5|6][1-4][1|2][2-4]	C30H21N3O4
488.3333	488.1605	0.1728	353.9	7.34	1	x3 -H2O -NH3 [1|2][1-5][2|3][3-5]	C30H21N3O4
495.2527	495.2714	-0.0187	-37.8	7.38	1	c3 [1|2][1-3]	C26H34N6O4
495.2527	495.2602	-0.0075	-15.1	7.38	1	b4 [4|5][1-4]	C27H34N4O5
495.2527	495.2602	-0.0075	-15.1	7.38	1	c4 -NH3 [4|5][1-4]	C27H34N4O5
495.2527	495.2391	0.0136	27.6	7.38	1	z4 -H2O -NH3 [5|6][1-5][2|3][2-5]	C30H30N4O3
495.2527	495.2391	0.0136	27.6	7.38	1	z4 -H2O -NH3 [2|3][1-5][2|3][2-5]	C30H30N4O3
499.4081	499.3027	0.1054	211.1	8.09	1	b4 [2|3][1-5][4|5][1-4]	C26H38N6O4
499.4081	499.3027	0.1054	211.1	8.09	1	b4 [6|1][1-5][2|3][1-4]	C26H38N6O4
499.4081	499.3027	0.1054	211.1	8.09	1	c4 -NH3 [6|1][1-5][2|3][1-4]	C26H38N6O4

Meas. m/z	Calc. m/z	δ (Da)	δ (ppm)	Rel. Int. (%)	z	Annotation	Formula
499.4081	499.3027	0.1054	211.1	8.09	1	c4 -NH3 [2|3][1-5][4|5][1-4]	C26H38N6O4
499.4081	499.2704	0.1377	275.9	8.09	1	a4 -NH3 -NH3 [2|3][1-4]	C30H34N4O3
499.4081	499.2340	0.1741	348.8	8.09	1	z4 -H2O [6|1][1-5][3|4][2-5]	C29H30N4O4
499.4081	499.2340	0.1741	348.8	8.09	1	b4 -H2O -NH3 [6|1][1-5][4|5][1-4]	C29H30N4O4
499.4081	499.2340	0.1741	348.8	8.09	1	z4 -H2O [4|5][1-5][1|2][2-5]	C29H30N4O4
499.4081	499.2340	0.1741	348.8	8.09	1	b4 -H2O -NH3 [4|5][1-5][2|3][1-4]	C29H30N4O4
527.3840	527.2976	0.0863	163.7	9.83	1	c4 +CO -NH3 [2|3][1-5][4|5][1-4]	C27H38N6O5
527.3840	527.2976	0.0863	163.7	9.83	1	c4 +CO -NH3 [6|1][1-5][2|3][1-4]	C27H38N6O5
527.3840	527.2976	0.0863	163.7	9.83	1	b4 +CO [2|3][1-5][4|5][1-4]	C27H38N6O5
527.3840	527.2976	0.0863	163.7	9.83	1	b4 +CO [6|1][1-5][2|3][1-4]	C27H38N6O5
527.3840	527.2653	0.1187	225.1	9.83	1	b4 -NH3 -NH3 [2|3][1-4]	C31H34N4O4
527.3840	527.2653	0.1187	225.1	9.83	1	z4 -NH3 [6|1][3-6]	C31H34N4O4
527.3840	527.2289	0.1551	294.1	9.83	1	b4 +CO -H2O -NH3 [4|5][1-5][2|3][1-4]	C30H30N4O5
527.3840	527.2289	0.1551	294.1	9.83	1	x4 -NH3 [4|5][1-5][3|4][2-5]	C30H30N4O5
527.3840	527.2289	0.1551	294.1	9.83	1	b4 +CO -H2O -NH3 [6|1][1-5][4|5][1-4]	C30H30N4O5
527.3840	527.2289	0.1551	294.1	9.83	1	x4 -NH3 [2|3][1-5][1|2][2-5]	C30H30N4O5
534.3412	534.2711	0.0702	131.3	15.41	1	c4 +CO -H2O -NH3 [5|6][1-5][4|5][1-4]	C29H35N5O5
534.3412	534.2711	0.0702	131.3	15.41	1	b4 +CO -H2O [5|6][1-5][4|5][1-4]	C29H35N5O5
534.3412	534.2711	0.0702	131.3	15.41	1	b4 +CO -H2O [3|4][1-5][2|3][1-4]	C29H35N5O5
534.3412	534.2711	0.0702	131.3	15.41	1	c4 -NH3 [6|1][1-5][4|5][1-4]	C29H35N5O5
534.3412	534.2711	0.0702	131.3	15.41	1	c4 -NH3 [4|5][1-5][2|3][1-4]	C29H35N5O5
534.3412	534.2711	0.0702	131.3	15.41	1	b4 [6|1][1-5][4|5][1-4]	C29H35N5O5
534.3412	534.2711	0.0702	131.3	15.41	1	c4 +CO -H2O -NH3 [3|4][1-5][2|3][1-4]	C29H35N5O5
534.3412	534.2711	0.0702	131.3	15.41	1	b4 [4|5][1-5][2|3][1-4]	C29H35N5O5
538.3838	538.3024	0.0814	151.2	14.77	1	c4 -NH3 [3|4][1-5][4|5][1-4]	C29H39N5O5
538.3838	538.3024	0.0814	151.2	14.77	1	c4 -NH3 [1|2][1-5][2|3][1-4]	C29H39N5O5
538.3838	538.3024	0.0814	151.2	14.77	1	b4 [1|2][1-5][2|3][1-4]	C29H39N5O5
538.3838	538.3024	0.0814	151.2	14.77	1	b4 [3|4][1-5][4|5][1-4]	C29H39N5O5
538.3838	538.2449	0.1389	258.1	14.77	1	x4 -H2O -NH3 [5|6][1-5][2|3][2-5]	C31H31N5O4
538.3838	538.2449	0.1389	258.1	14.77	1	x4 -H2O -NH3 [2|3][1-5][2|3][2-5]	C31H31N5O4
545.3820	545.2871	0.0949	174.0	14.22	1	c4 +CO -H2O [4|5][1-5][4|5][1-4]	C30H36N6O4
545.3820	545.2871	0.0949	174.0	14.22	1	b4 -H2O [6|1][1-4]	C30H36N6O4
545.3820	545.2871	0.0949	174.0	14.22	1	c4 +CO -H2O [2|3][1-5][2|3][1-4]	C30H36N6O4
545.3820	545.2871	0.0949	174.0	14.22	1	c4 -H2O -NH3 [6|1][1-4]	C30H36N6O4
545.3820	545.2758	0.1061	194.6	14.22	1	b4 +H2O -NH3 -NH3 [2|3][1-4]	C31H36N4O5
545.3820	545.2395	0.1425	261.3	14.22	1	b4 +CO -NH3 [4|5][1-5][2|3][1-4]	C30H32N4O6
545.3820	545.2395	0.1425	261.3	14.22	1	b4 +CO -NH3 [6|1][1-5][4|5][1-4]	C30H32N4O6
561.4036	561.3184	0.0852	151.8	46.73	1	b4 [2|3][1-4]	C31H40N6O4
561.4036	561.3184	0.0852	151.8	46.73	1	c4 -NH3 [2|3][1-4]	C31H40N6O4
561.4036	561.3071	0.0964	171.8	46.73	1	a5 -NH3 -NH3 [3|4][1-5]	C32H40N4O5
561.4036	561.2820	0.1216	216.6	46.73	1	c4 +CO -H2O [6|1][1-5][4|5][1-4]	C30H36N6O5
561.4036	561.2820	0.1216	216.6	46.73	1	c4 +CO -H2O [4|5][1-5][2|3][1-4]	C30H36N6O5
561.4036	561.2708	0.1328	236.6	46.73	1	b4 +H2O -NH3 -NH3 [1|2][1-4]	C31H36N4O6
561.4036	561.2496	0.1539	274.3	46.73	1	b4 -H2O -NH3 [1|2][1-5][3|4][1-4]	C34H32N4O4
561.4036	561.2496	0.1539	274.3	46.73	1	z4 -H2O [1|2][1-5][2|3][2-5]	C34H32N4O4
561.4036	561.2496	0.1539	274.3	46.73	1	z4 -H2O [4|5][1-5][2|3][2-5]	C34H32N4O4
561.4036	561.2496	0.1539	274.3	46.73	1	b4 -H2O -NH3 [4|5][1-5][3|4][1-4]	C34H32N4O4
563.3751	563.2976	0.0775	137.5	58.19	1	c4 +CO [4|5][1-5][4|5][1-4]	C30H38N6O5
563.3751	563.2976	0.0775	137.5	58.19	1	c4 -NH3 [6|1][1-4]	C30H38N6O5
563.3751	563.2976	0.0775	137.5	58.19	1	b4 [6|1][1-4]	C30H38N6O5
563.3751	563.2976	0.0775	137.5	58.19	1	c4 +CO [2|3][1-5][2|3][1-4]	C30H38N6O5
563.3751	563.2653	0.1098	195.0	58.19	1	a4 -NH3 -NH3 [1|2][1-5][4|5][1-4]	C34H34N4O4
563.3751	563.2653	0.1098	195.0	58.19	1	a4 -NH3 -NH3 [5|6][1-5][2|3][1-4]	C34H34N4O4
577.3938	577.3497	0.0441	76.4	51.56	1	a5 -H2O [3|4][1-5]	C32H44N6O4

Meas. m/z	Calc. m/z	δ (Da)	δ (ppm)	Rel. Int. (%)	z	Annotation	Formula
577.3938	577.3133	0.0805	139.4	51.56	1	b4 [1|2][1-4]	C31H40N6O5
577.3938	577.3133	0.0805	139.4	51.56	1	c4 -NH3 [1|2][1-4]	C31H40N6O5
582.3539	582.2711	0.0828	142.2	12.19	1	c4 -NH3 [5|6][1-4]	C33H35N5O5
582.3539	582.2711	0.0828	142.2	12.19	1	b4 [5|6][1-4]	C33H35N5O5
591.3881	591.2926	0.0956	161.6	6.52	1	c4 +CO -NH3 [6|1][1-4]	C31H38N6O6
591.3881	591.2926	0.0956	161.6	6.52	1	b4 +CO [6|1][1-4]	C31H38N6O6
591.3881	591.2602	0.1279	216.4	6.52	1	z4 -NH3 [5|6][1-5][1|2][2-5]	C35H34N4O5
591.3881	591.2602	0.1279	216.4	6.52	1	b4 -NH3 -NH3 [5|6][1-5][2|3][1-4]	C35H34N4O5
591.3881	591.2602	0.1279	216.4	6.52	1	b4 -NH3 -NH3 [1|2][1-5][4|5][1-4]	C35H34N4O5
591.3881	591.2602	0.1279	216.4	6.52	1	z4 -NH3 [1|2][1-5][3|4][2-5]	C35H34N4O5
591.3881	591.2238	0.1643	277.9	6.52	1	x4 -NH3 [3|4][3-6]	C34H30N4O6
596.3901	596.2867	0.1034	173.4	10.06	1	b4 [4|5][1-5][3|4][1-4]	C34H37N5O5
596.3901	596.2867	0.1034	173.4	10.06	1	b4 [1|2][1-5][3|4][1-4]	C34H37N5O5
596.3901	596.2867	0.1034	173.4	10.06	1	c4 -NH3 [1|2][1-5][3|4][1-4]	C34H37N5O5
596.3901	596.2867	0.1034	173.4	10.06	1	c4 -NH3 [4|5][1-5][3|4][1-4]	C34H37N5O5
605.4070	605.3446	0.0624	103.1	5.58	1	c5 -H2O -NH3 [3|4][1-5]	C33H44N6O5
605.4070	605.3446	0.0624	103.1	5.58	1	b5 -H2O [3|4][1-5]	C33H44N6O5
605.4070	605.3082	0.0988	163.2	5.58	1	b4 +CO [1|2][1-4]	C32H40N6O6
605.4070	605.3082	0.0988	163.2	5.58	1	c4 +CO -NH3 [1|2][1-4]	C32H40N6O6
605.4070	605.2395	0.1675	276.8	5.58	1	x4 -NH3 [1|2][1-5][2|3][2-5]	C35H32N4O6
605.4070	605.2395	0.1675	276.8	5.58	1	x4 -NH3 [4|5][1-5][2|3][2-5]	C35H32N4O6
623.3922	623.3552	0.0370	59.3	11.87	1	b5 [3|4][1-5]	C33H46N6O6
623.3922	623.3552	0.0370	59.3	11.87	1	c5 -NH3 [3|4][1-5]	C33H46N6O6
623.3922	623.2976	0.0945	151.6	11.87	1	c4 +CO -H2O [1|2][1-5][3|4][1-4]	C35H38N6O5
623.3922	623.2976	0.0945	151.6	11.87	1	c4 +CO -H2O [4|5][1-5][3|4][1-4]	C35H38N6O5
644.4281	644.3555	0.0726	112.7	50.63	1	b5 -H2O [6|1][1-5]	C35H45N7O5
644.4281	644.3555	0.0726	112.7	50.63	1	c5 -H2O -NH3 [6|1][1-5]	C35H45N7O5
646.4065	646.3711	0.0353	54.7	11.16	1	c5 -NH3 [2|3][1-5]	C35H47N7O5
646.4065	646.3711	0.0353	54.7	11.16	1	b5 [2|3][1-5]	C35H47N7O5
646.4065	646.3235	0.0830	128.4	11.16	1	b5 +H2O -NH3 -NH3 [6|1][1-5]	C35H43N5O7
646.4065	646.3024	0.1041	161.1	11.16	1	b5 -H2O -NH3 [4|5][1-5]	C38H39N5O5
646.4065	646.3024	0.1041	161.1	11.16	1	z5 -H2O [3|4][2-6]	C38H39N5O5
651.4030	651.3501	0.0529	81.3	26.81	1	b5 +CO [3|4][1-5]	C34H46N6O7
651.4030	651.3501	0.0529	81.3	26.81	1	c5 +CO -NH3 [3|4][1-5]	C34H46N6O7
651.4030	651.2926	0.1105	169.6	26.81	1	x4 [1|2][1-5][3|4][2-5]	C36H38N6O6
651.4030	651.2926	0.1105	169.6	26.81	1	x4 [5|6][1-5][1|2][2-5]	C36H38N6O6
662.4099	662.3661	0.0439	66.2	26.60	1	c5 -NH3 [6|1][1-5]	C35H47N7O6
662.4099	662.3661	0.0439	66.2	26.60	1	b5 [6|1][1-5]	C35H47N7O6
662.4099	662.3337	0.0762	115.1	26.60	1	a5 -NH3 -NH3 [1|2][1-5]	C39H43N5O5
674.4370	674.3661	0.0709	105.1	30.23	1	b5 +CO [2|3][1-5]	C36H47N7O6
674.4370	674.3661	0.0709	105.1	30.23	1	c5 +CO -NH3 [2|3][1-5]	C36H47N7O6
674.4370	674.2973	0.1396	207.1	30.23	1	b5 +CO -H2O -NH3 [4|5][1-5]	C39H39N5O6
680.4169	680.3766	0.0402	59.2	18.29	1	b5 +H2O [6|1][1-5]	C35H49N7O7
680.4169	680.3555	0.0614	90.2	18.29	1	c5 -H2O [4|5][1-5]	C38H45N7O5
681.3304	681.3395	-0.0091	-13.3	40.69	1	b5 [4|5][1-5]	C38H44N6O6
681.3304	681.3395	-0.0091	-13.3	40.69	1	c5 -NH3 [4|5][1-5]	C38H44N6O6
692.3960	692.3555	0.0405	58.5	20.08	1	c5 -H2O -NH3 [5|6][1-5]	C39H45N7O5
692.3960	692.3555	0.0405	58.5	20.08	1	b5 -H2O [5|6][1-5]	C39H45N7O5
692.3960	692.3079	0.0881	127.3	20.08	1	b5 +CO -NH3 [4|5][1-5]	C39H41N5O7
710.3961	710.3661	0.0300	42.2	59.58	1	b5 [5|6][1-5]	C39H47N7O6
710.3961	710.3661	0.0300	42.2	59.58	1	c5 -NH3 [5|6][1-5]	C39H47N7O6
724.4221	724.3817	0.0404	55.8	64.23	1	b5 [1|2][1-5]	C40H49N7O6
724.4221	724.3817	0.0404	55.8	64.23	1	c5 -NH3 [1|2][1-5]	C40H49N7O6
725.4431	725.3657	0.0774	106.7	22.71	1	b5 +H2O -NH3 [1|2][1-5]	C40H48N6O7

Meas. m/z	Calc. m/z	δ (Da)	δ (ppm)	Rel. Int. (%)	z	Annotation	Formula
734.3809	734.3661	0.0148	20.2	19.28	1	b5 +CO -H2O [1|2][1-5]	C41H47N7O6
734.3809	734.3661	0.0148	20.2	19.28	1	c5 +CO -H2O -NH3 [1|2][1-5]	C41H47N7O6
752.4416	752.3766	0.0650	86.4	50.17	1	c5 +CO -NH3 [1|2][1-5]	C41H49N7O7
752.4416	752.3766	0.0650	86.4	50.17	1	b5 +CO [1|2][1-5]	C41H49N7O7

Generated by mMass • Open Source Mass Spectrometry Tool • www.mmass.org

mMass Report: *Microginin FR1*

**Date**	Sun Aug 14 16:16:37 2011	**Scan Number**	0
**Operator**	Timo Niedermeyer	**Retention Time**	660.1
**Contact**	timo.niedermeyer@cyano-biotech.com	**MS Level**	2
**Institution**	Cyano Biotech GmbH	**Precursor m/z**	728.95
**Instrument**	Shimadzu LCMS-IT-TOF ESI IT-TOF	**Polarity**	positive
		**Spectrum Points**	1260
		**Peak List**	14

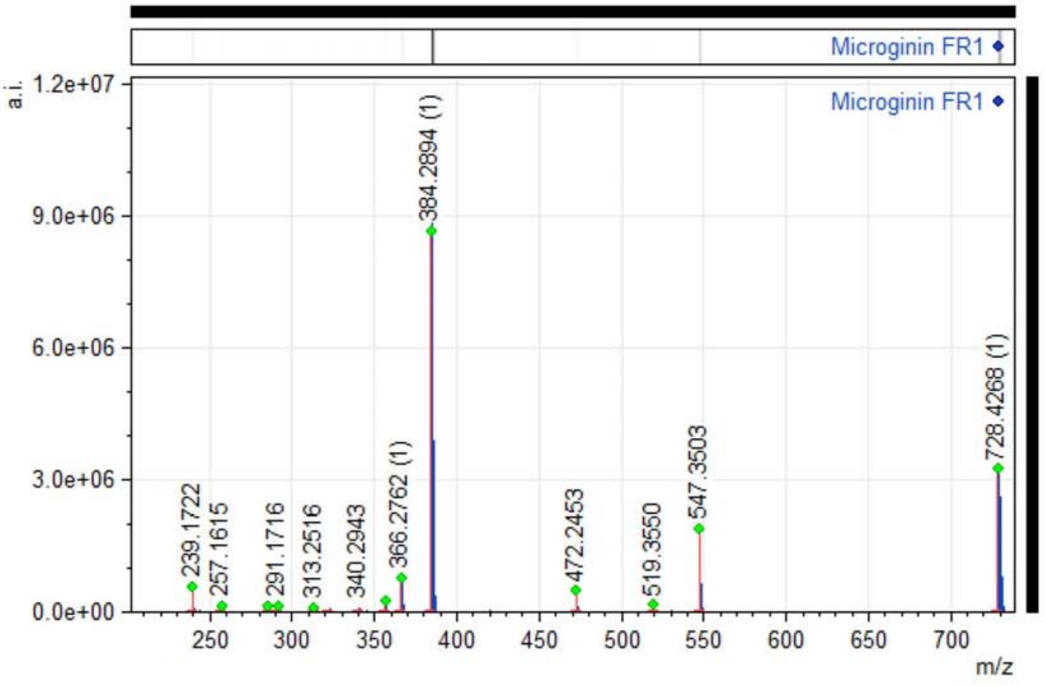

Sequence - *Microginin FR-1*

Accession	Length	Mo. Mass	Av. Mass	Coverage	Matched Int.
	5	727.4156	727.8889	100.0 %	99.1 %

Ahda | Ala | NMe-Leu | Tyr | Tyr

Meas. m/z	Calc. m/z	δ (Da)	δ (ppm)	Rel. Int. (%)	z	Annotation	Formula
239.1722	239.1754	-0.0032	-13.5	6.56	1	b2 -H2O [1-2]	C13H22N2O2
239.1722	239.1754	-0.0032	-13.5	6.56	1	c2 -H2O -NH3 [1-2]	C13H22N2O2
257.1615	257.1496	0.0119	46.2	1.53	1	c3 -C8H17N -NH3 [1-3]	C12H20N2O4
257.1615	257.1496	0.0119	46.2	1.53	1	b3 -C8H17N [1-3]	C12H20N2O4
285.2539	285.2537	0.0003	0.9	1.27	1	a2 [1-3][2|3][1-2]	C16H32N2O2
291.1716	291.1703	0.0013	4.4	1.42	1	c2 -NH3 [1-5][2|3][1-2]	C16H22N2O3
291.1716	291.1703	0.0013	4.4	1.42	1	b2 [1-5][2|3][1-2]	C16H22N2O3
313.2516	313.2486	0.0030	9.5	1.15	1	b2 [1-3][2|3][1-2]	C17H32N2O3
313.2516	313.2486	0.0030	9.5	1.15	1	c2 -NH3 [1-3][2|3][1-2]	C17H32N2O3
356.2935	356.2908	0.0028	7.7	2.56	1	a3 [1-3]	C19H37N3O3
366.2762	366.2751	0.0010	2.8	8.79	1	b3 -H2O [1-3]	C20H35N3O3
366.2762	366.2751	0.0010	2.8	8.79	1	c3 -H2O -NH3 [1-3]	C20H35N3O3

Meas. m/z	Calc. m/z	δ (Da)	δ (ppm)	Rel. Int. (%)	z	Annotation	Formula
384.2894	384.2857	0.0037	9.6	100.00	1	b3 [1-3]	C20H37N3O4
384.2894	384.2857	0.0037	9.6	100.00	1	c3 -NH3 [1-3]	C20H37N3O4
472.2453	472.2442	0.0011	2.4	5.40	1	b3 +H2O [1-5][2|3][1-3]	C25H33N3O6
519.3550	519.3541	0.0009	1.8	1.80	1	a4 [1-4]	C28H46N4O5
519.3550	519.3541	0.0009	1.8	1.80	1	a4 [1-5][4|5][1-4]	C28H46N4O5
547.3503	547.3490	0.0013	2.3	21.71	1	b4 [1-5][4|5][1-4]	C29H46N4O6
547.3503	547.3490	0.0013	2.3	21.71	1	c4 -NH3 [1-4]	C29H46N4O6
547.3503	547.3490	0.0013	2.3	21.71	1	c4 -NH3 [1-5][4|5][1-4]	C29H46N4O6
547.3503	547.3490	0.0013	2.3	21.71	1	b4 [1-4]	C29H46N4O6
728.4268	728.4229	0.0039	5.3	37.34	1	M [1-5]	C38H57N5O9

Generated by mMass • Open Source Mass Spectrometry Tool • www.mmass.org
