# Supplementary material for: mMass as a Software Tool for the Annotation of Cyclic Peptide Tandem Mass Spectra
Source: PLoS One. 2012 Sep 13;7(9):e44913. doi: 10.1371/journal.pone.0044913 (PMC3441486; doi:10.1371/journal.pone.0044913)
Supplement: Example S3 — Workflow for the annotation of mass spectra. (PDF) [file pone.0044913.s005.pdf]

Workflow for the annotation of mass spectra

- Start mMass and open data file
- Pick peaks either automatically or by hand
- Open sequence Editor (Sequence → New…)
- Chose "Custom" from the Sequence Type dropdown menu; if the peptide is cyclic, tick the respective checkbox.

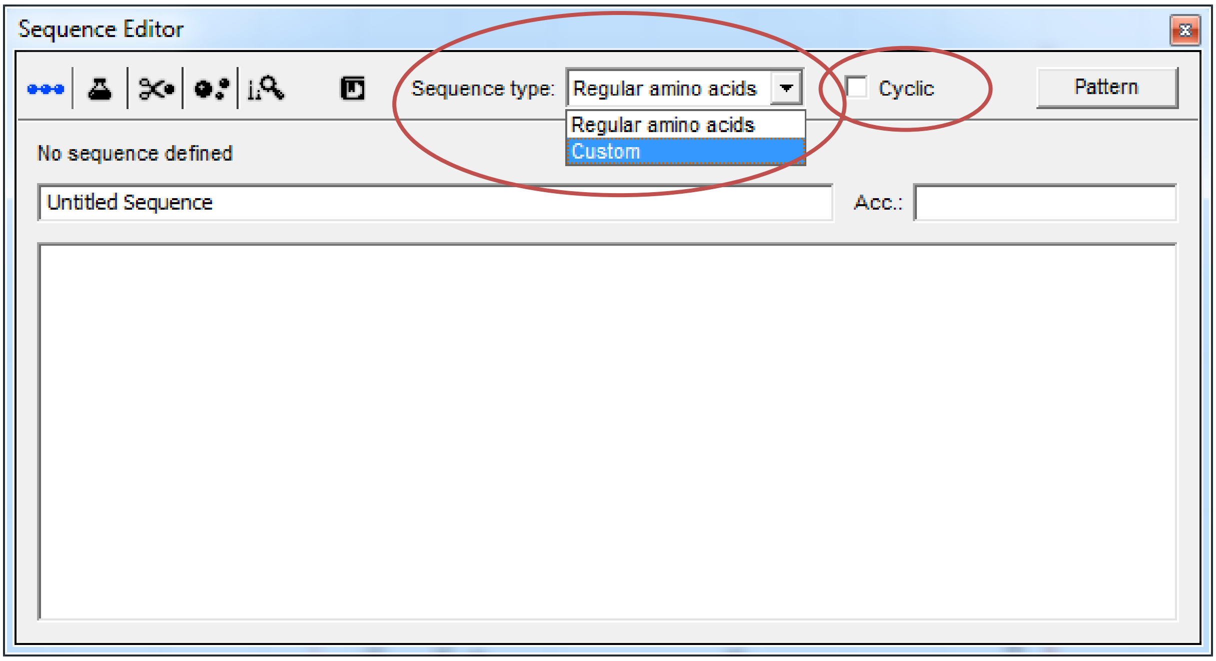

- Enter the monomers the peptide is composed of. The peptide length is limited to 20 monomers. The mass of the peptide and the numbers of monomers it is composed of are updated on-the-fly.

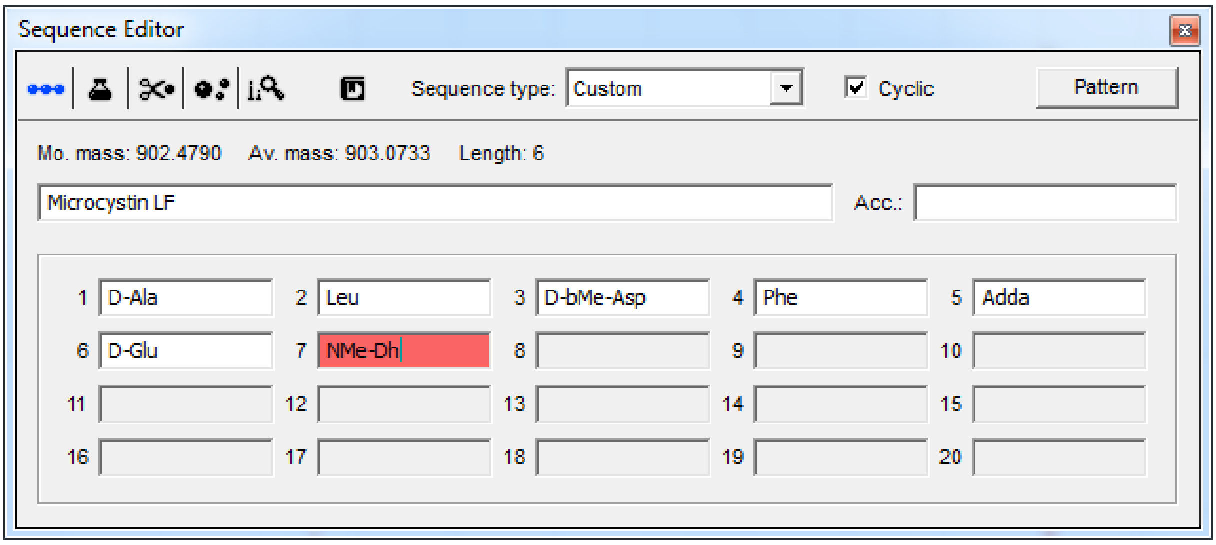

If an entered monomer abbreviation is not present in the monomer library, the cell is shaded red as shown above.

If the user does not remember the abbreviation of a monomer, a searchable monomer list can be opened to look up the respective abbreviations:

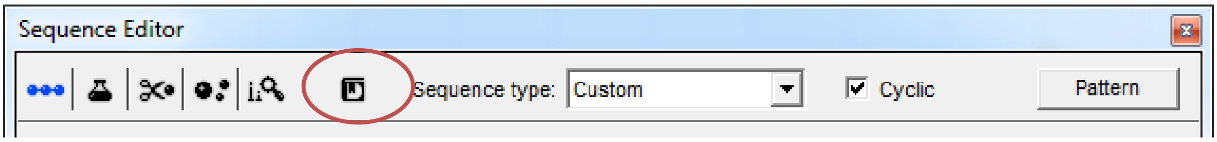

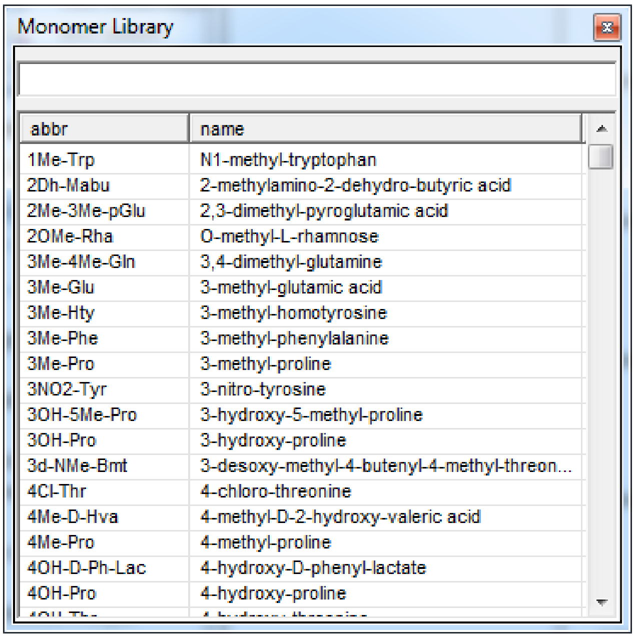

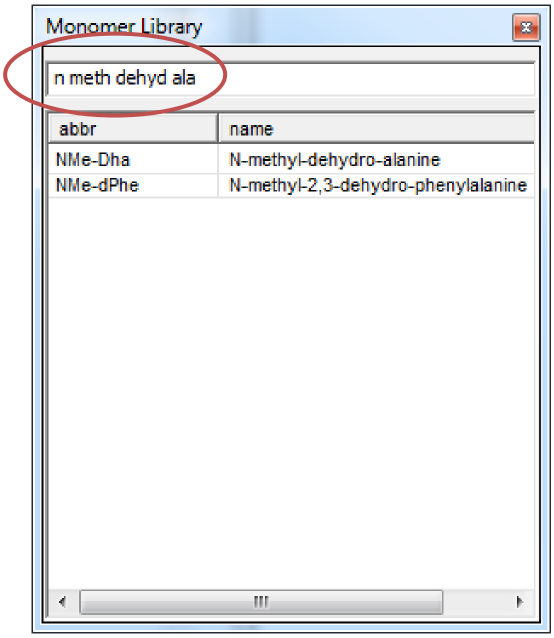

Monomers can also conveniently be dragged and dropped from this list into the Sequence Editor.

- When the sequence is composed (Microcystin LF in this example), choose the Peptide Fragmentation tool in the Sequence editor toolbar:

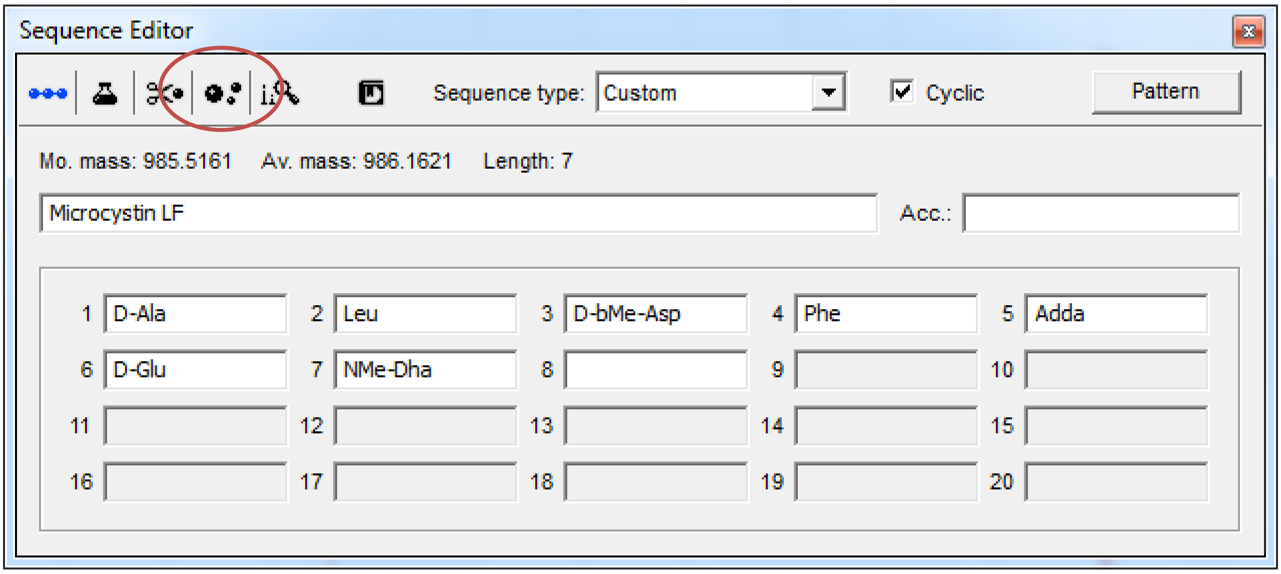

- Choose the fragments you wish to calculate (in this example only M and b-ions and fragments with neutral losses) and if the masses that are calculated shall be monoisotopic or average masses. Click on the button “Fragment”.

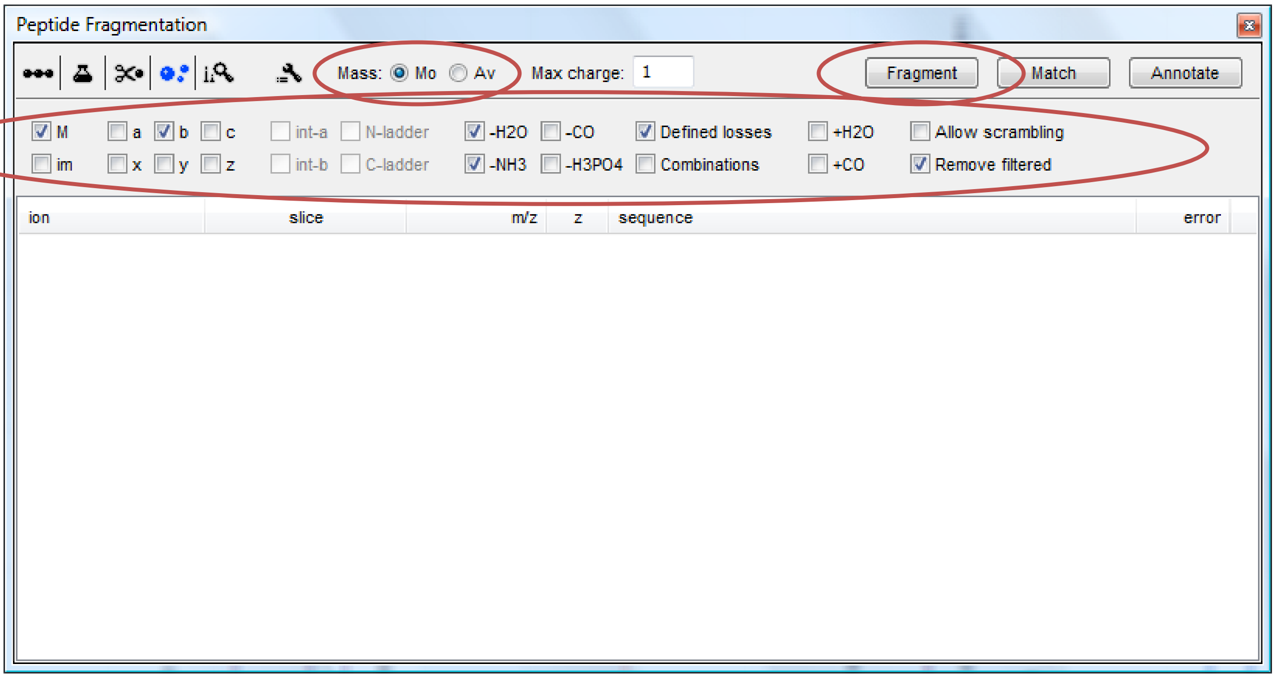

- All theoretical fragments complying with the chosen fragmentation pathways are calculated and displayed as a list.

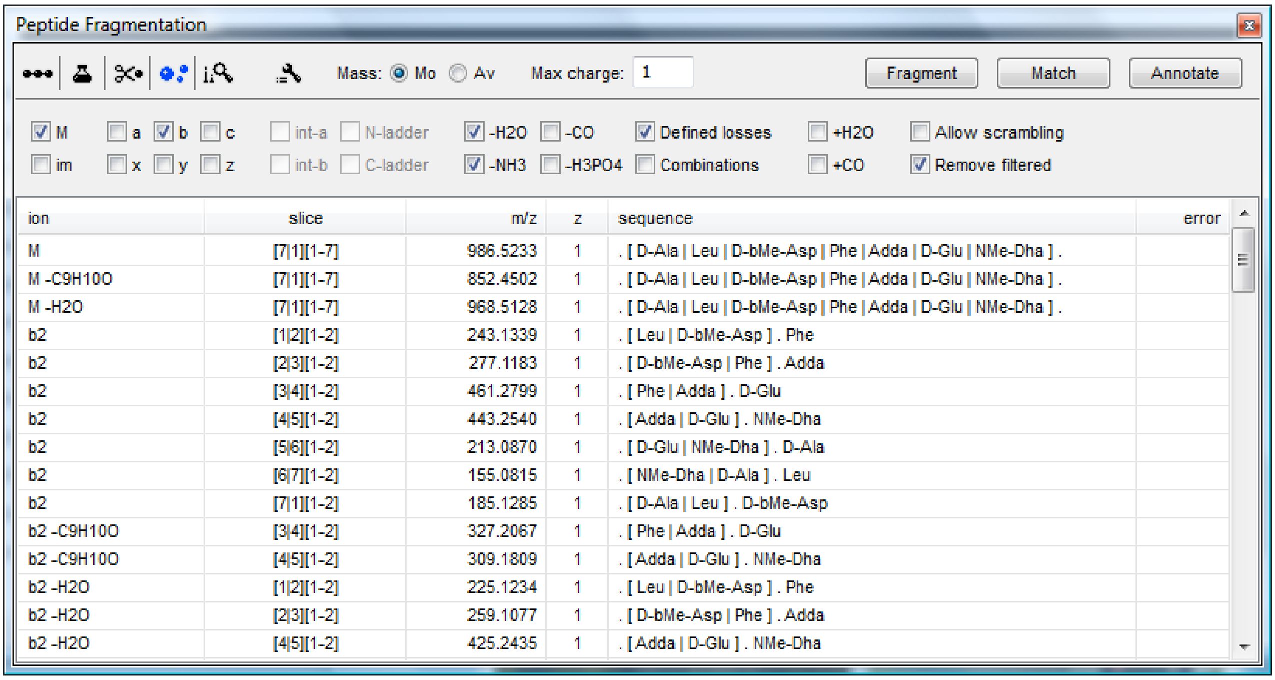

ion	slice	m/z	z	sequence	error
M	[7|1][1-7]	986.5233	1	. [D-Ala | Leu | D-bMe-Asp | Phe | Adda | D-Glu | NMe-Dha] .	
M -C9H10O	[7|1][1-7]	852.4502	1	. [D-Ala | Leu | D-bMe-Asp | Phe | Adda | D-Glu | NMe-Dha] .	
M -H2O	[7|1][1-7]	968.5128	1	. [D-Ala | Leu | D-bMe-Asp | Phe | Adda | D-Glu | NMe-Dha] .	
b2	[1|2][1-2]	243.1339	1	. [Leu | D-bMe-Asp] . Phe	
b2	[2|3][1-2]	277.1183	1	. [D-bMe-Asp | Phe] . Adda	
b2	[3|4][1-2]	461.2799	1	. [Phe | Adda] . D-Glu	
b2	[4|5][1-2]	443.2540	1	. [Adda | D-Glu] . NMe-Dha	
b2	[5|6][1-2]	213.0870	1	. [D-Glu | NMe-Dha] . D-Ala	
b2	[6|7][1-2]	155.0815	1	. [NMe-Dha | D-Ala] . Leu	
b2	[7|1][1-2]	185.1285	1	. [D-Ala | Leu] . D-bMe-Asp	
b2 -C9H10O	[3|4][1-2]	327.2067	1	. [Phe | Adda] . D-Glu	
b2 -C9H10O	[4|5][1-2]	309.1809	1	. [Adda | D-Glu] . NMe-Dha	
b2 -H2O	[1|2][1-2]	225.1234	1	. [Leu | D-bMe-Asp] . Phe	
b2 -H2O	[2|3][1-2]	259.1077	1	. [D-bMe-Asp | Phe] . Adda	
b2 -H2O	[4|5][1-2]	425.2435	1	. [Adda | D-Glu] . NMe-Dha	

In this list, the type of the ion (M, a-ion, b-ion, …), the slice of the cyclic peptide that is the basis for the respective fragment ion (for the nomenclature see the paper), the calculated m/z for the ion, its charge, and the complete sequence of the fragment are displayed.

- Click on the button “Match”.

- In the resulting window, indicate the peak tolerance and click the button “Match”. This results in a first overview on the experimental peaks that can be matched with calculated theoretical fragments.

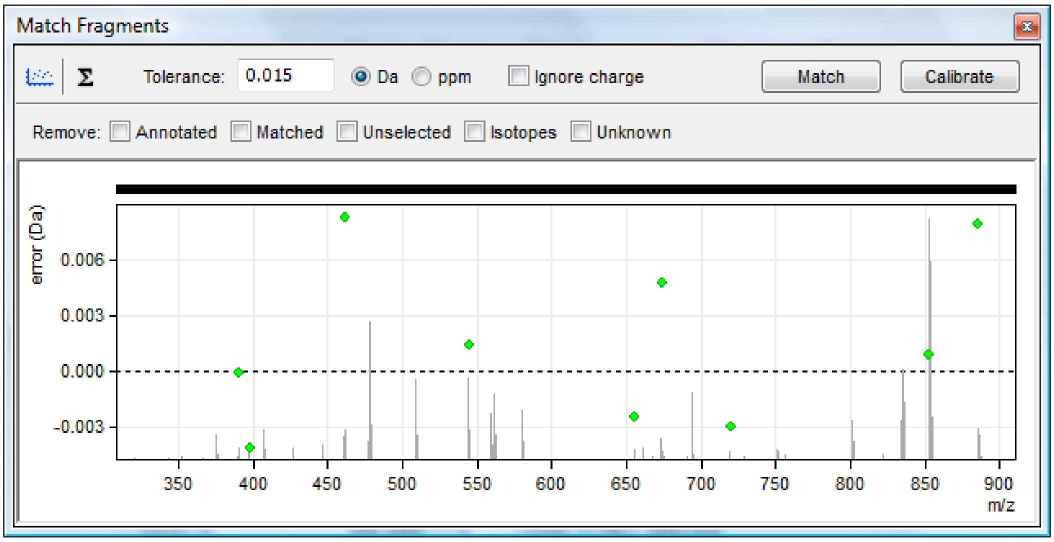

- Return to the Peptide Fragmentation window. Note that all matched ions are now typeset in bold and green. Also, the error between the experimental m/z and the calculated m/z is now given in the list.

  It is possible to only show matched peaks be right-clicking in the list and choosing the option “Show Matched Only”.

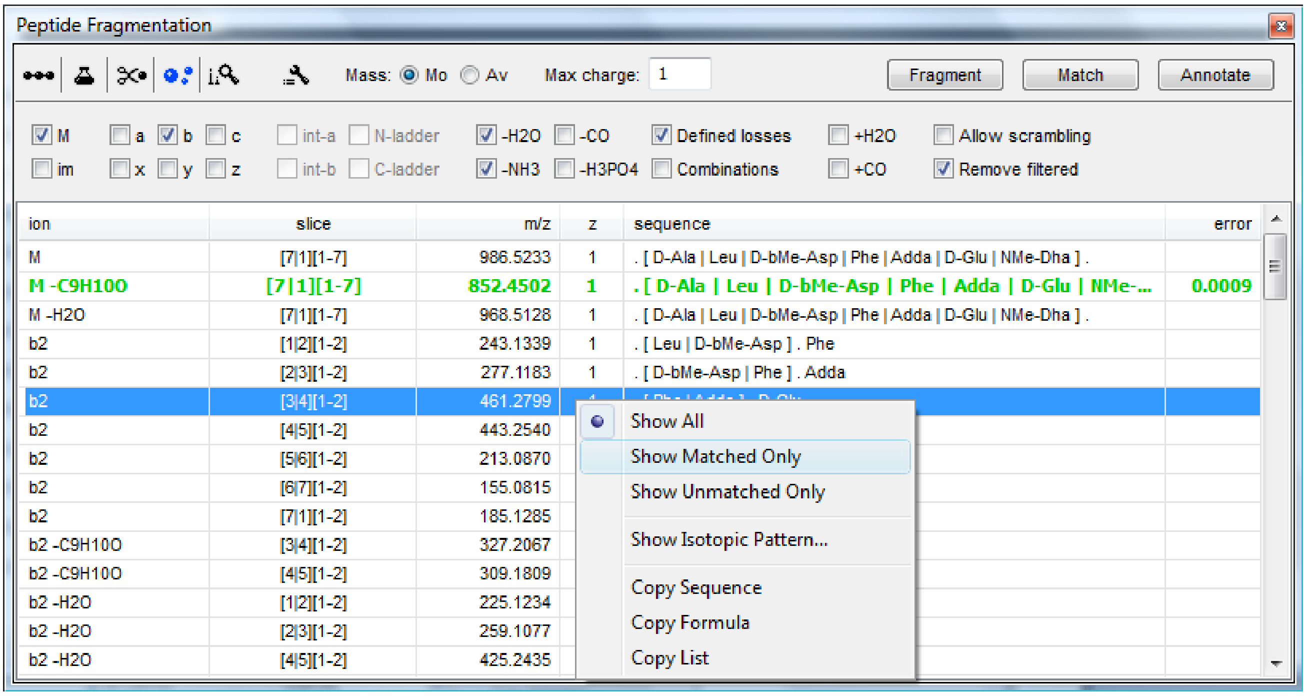

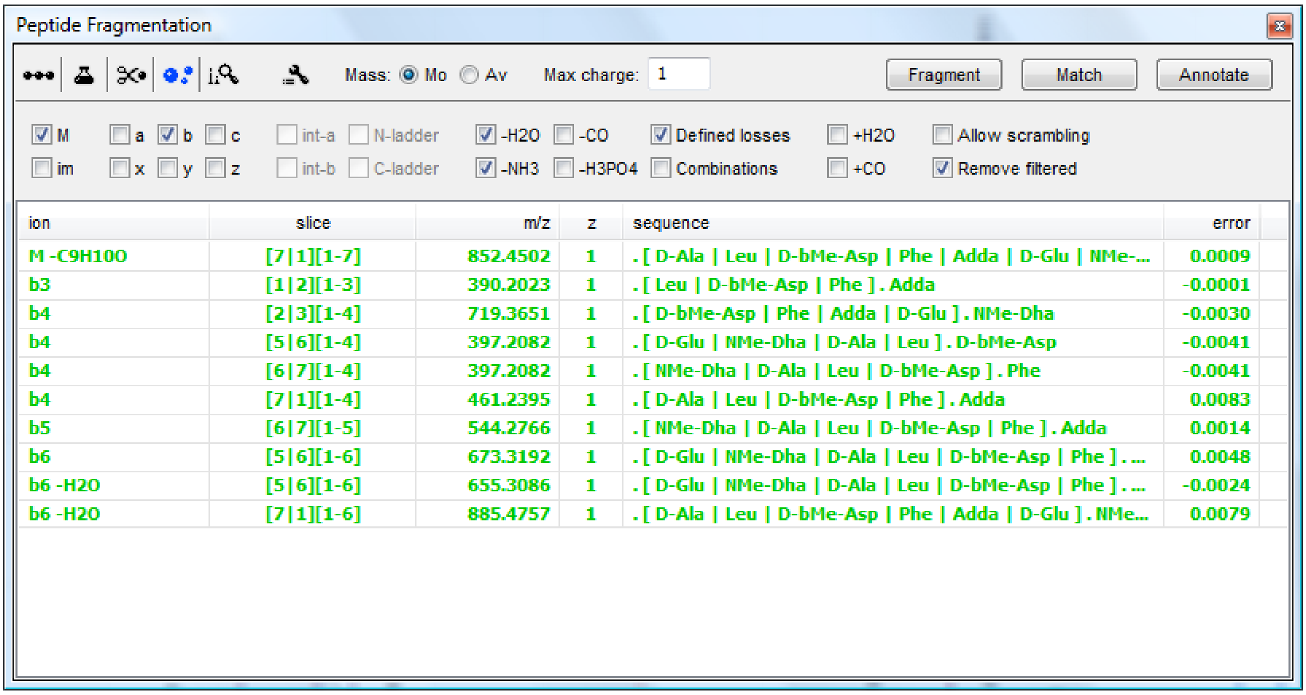

- Click on the button "Annotate".
- In the Documents Panel in the main window you will now find a list of all annotated peaks below your sequence:

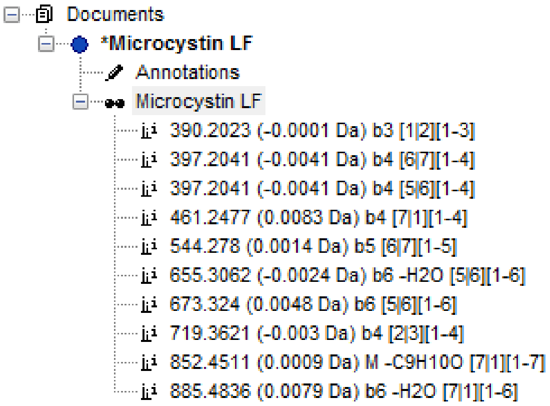

- After selection of a match by clicking on it, a match can be edited or deleted by right-clicking on the match and selecting the appropriate action. Deletion of matches might be necessary if matches are theoretically possible only but are not plausible from an experimental point of view.
  The software – as all software – can only help the analyst to identify possibilities. It is the responsibility of the analyst to judge if possibilities indicated by mMass indeed do make sense under the experimental circumstances used!

- In the Main Window, choose View → Notations → Show Notations and also choose with which information peaks should be annotated.

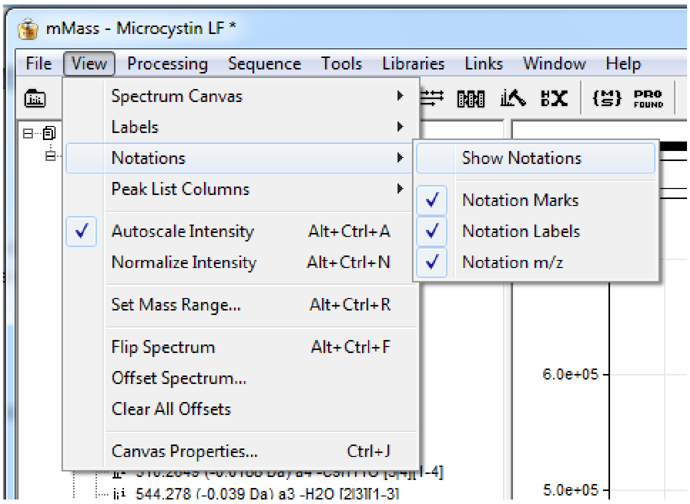

- Enjoy your annotated spectrum! You can also create a report using the build-in report generator.

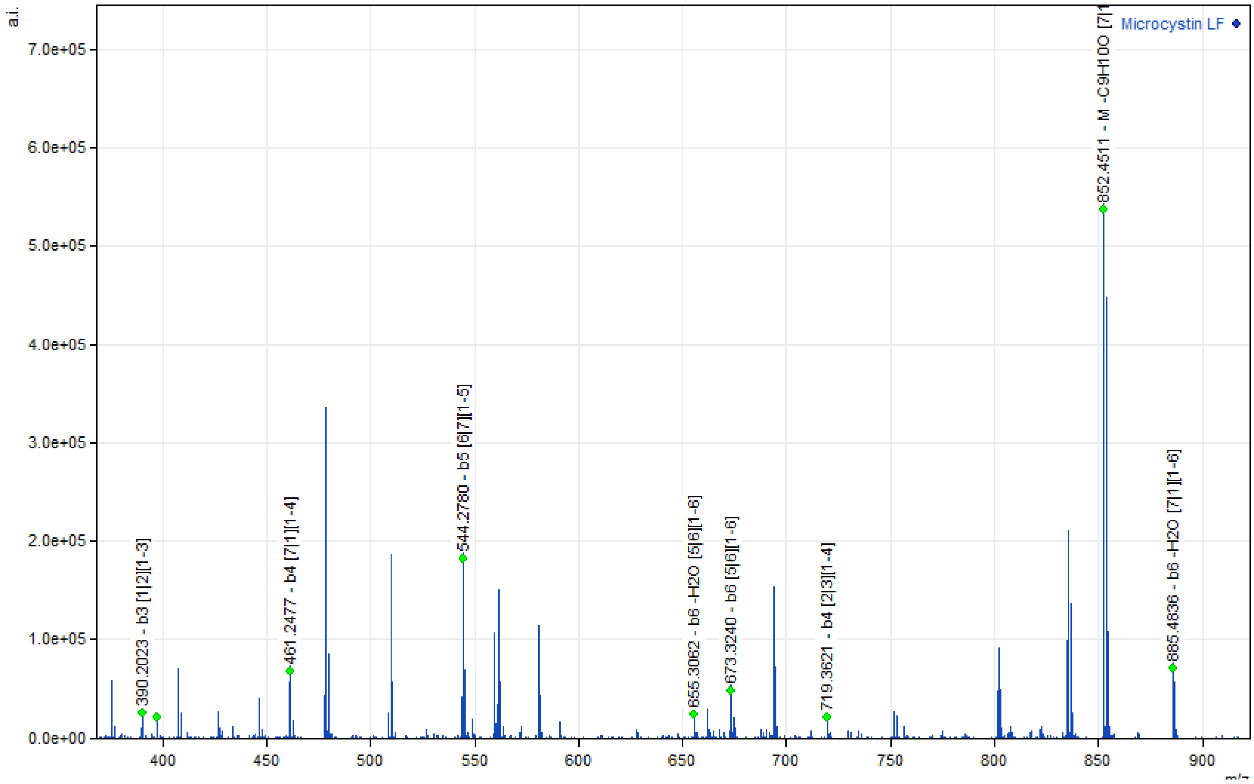

Please consult the mMass manual for further detailed descriptions of the capabilites of mMass. The manual is included in all mMass distributions and can also be downloaded separately from the mMass website at http://www.mmass.org/
